# π-extended [12]cycloparaphenylenes: from a hexaphenylbenzene cyclohexamer to its unexpected *C*_2_-symmetric congener†
†Electronic supplementary information (ESI) available: Experimental protocols, crystallographic data for **3** and **5**, ^1^H NMR, ^13^C NMR, 2D NMR, MALDI-TOF MS, as well as computational details. CCDC 1033267 1033268. For ESI and crystallographic data in CIF or other electronic format see DOI: 10.1039/c5sc02547h



**DOI:** 10.1039/c5sc02547h

**Published:** 2015-09-08

**Authors:** Florian E. Golling, Silvio Osella, Martin Quernheim, Manfred Wagner, David Beljonne, Klaus Müllen

**Affiliations:** a Max Planck Institute for Polymer Research , Ackermannweg 10 , 55128 Mainz , Germany . Email: muellen@mpip-mainz.mpg.de; b Graduate School Materials Science in Mainz , Staudinger Weg 9 , 55128 Mainz , Germany; c Chimie des Matériaux Nouveaux & Centre d'Innovation et de Recherche en Matériaux Polymères , Université de Mons-UMONS/Materia Nova , Place du Parc 20 , 7000 Mons , Belgium

## Abstract

The synthesis of π-extended [12]cycloparaphenylene (CPP) derivatives from a kinked triangular macrocycle is presented. Depending on the reaction conditions for reductive aromatization, either a hexaphenylbenzene cyclohexamer or its *C*_2_-symmetric congener was obtained. Their structures were confirmed by NMR spectroscopy or X-ray crystallographic analysis. With the support of DFT calculations, a mechanistic explanation for the unexpected formation of the oval shaped bis(cyclohexadiene)-bridged *C*_2_-symmetric macrocycle is provided. The here employed congested hexaphenylbenzene mode of connectivity in conjunction with a non-strained precursor improves oxidative cyclodehydrogenation toward the formation of ultrashort carbon nanotubes (CNT)s. Thus, this strategy can pave the way for new conceptual approaches of a solution-based bottom-up synthesis of CNTs.

## Abstract 2

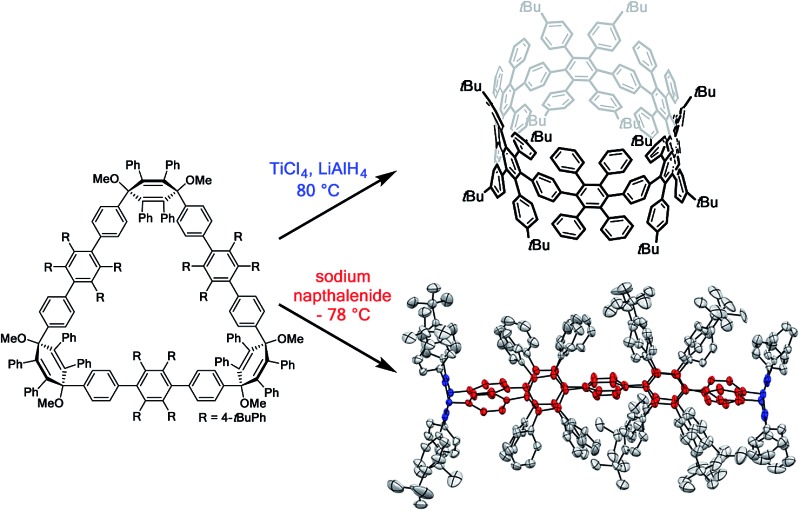
Based on a π-extended [12]CPP, two different precursors for the bottom-up synthesis of CNTs were synthesized. The congested hexaphenylbenzene mode of connectivity of the two macrocycles reveals an improved oxidative cyclodehydrogenation over previous reported strategies.

## Introduction

Carbon nanotubes (CNT)s have gained a great deal of attention over the last decades due to their attractive physical and electronic properties.^1^ Special emphasis has been put on the controlled synthesis of CNTs, since their electronic features sensitively depend on size and shape (edge structure, chirality).^2^ Cycloparaphenylenes (CPP)s are envisioned as precursors for a concise bottom-up synthesis of carbon nanobelts, *i.e.*, ultrashort CNTs.^3,4^ On surfaces, CPPs,^5^ besides other templates,^6^ have been successfully used for the growth of diameter-defined CNTs, whereas the advancement in solution-based approaches continues to be limited.^7^

**Fig. 1:**
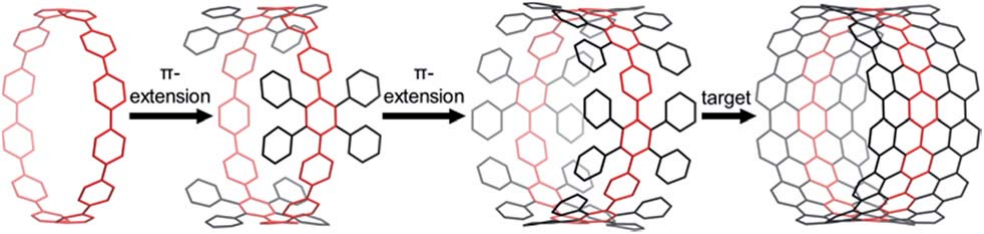
Schematic modes of π-extension: CPP (left), insufficiently π-extended CPP (second left), fully π-extended CPP to give a nanobelt precursor (second right), and CNT (right).

To reach this goal, several π-extended CPPs^8^ and structurally closely related derivatives^9^ have been synthesized over the last years. However, a necessary degree of axial π-extension of these structures, from a CPP to a nanobelt precursor (Fig. 1), was not accomplished to enable a solution-based bottom-up synthesis. In contrast, [9]cyclo-1,4-naphthylene^10^ and a [10]CPP dimer,^11^ are sufficiently π-extended and can thus be regarded as carbon nanobelt precursors, but dehydrogenation of the products was not reported.

**Fig. 2:**
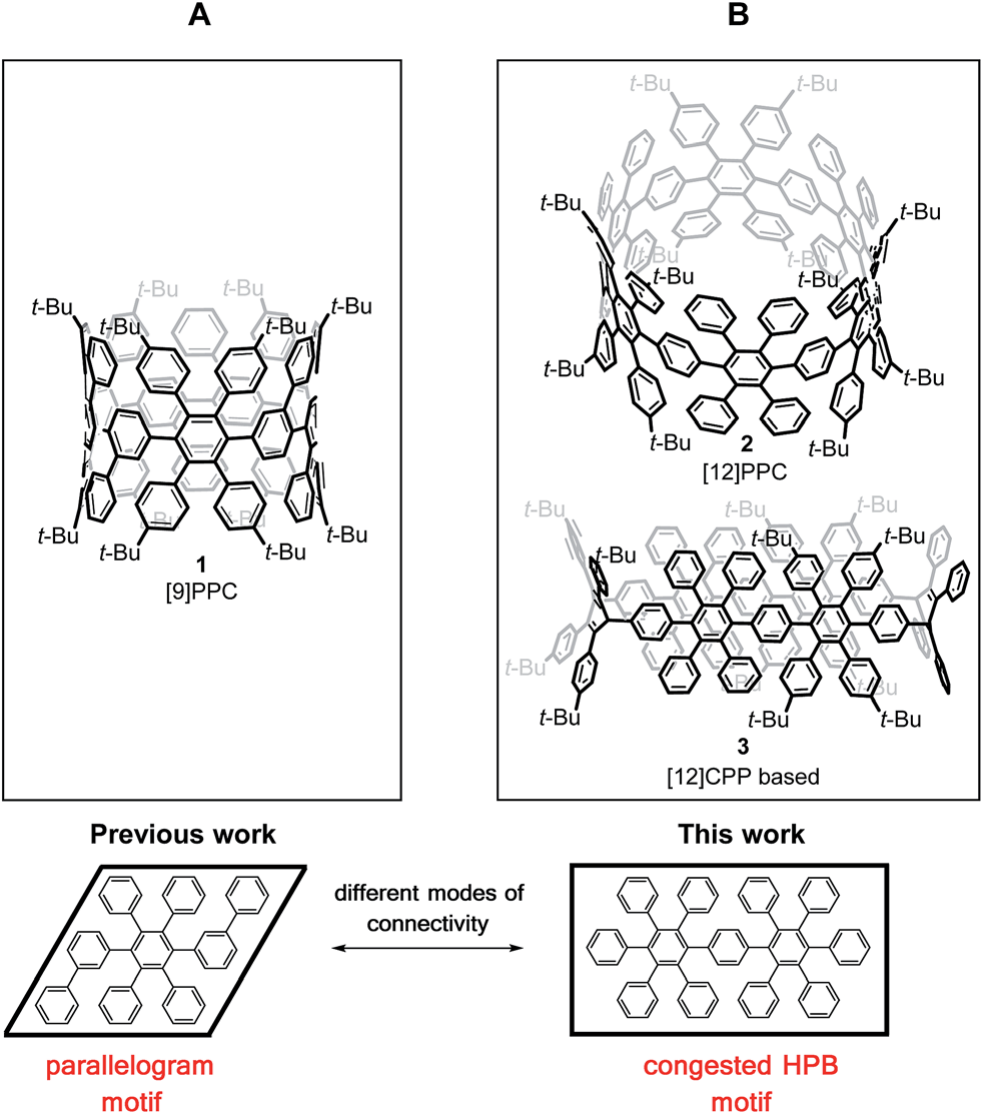
Two different modes of connectivity. Previous work (left): CNT synthesis *via* the parallelogram motif A (left) using [9]PPC 1 and [15]PPC (not shown); in this work (right): CNTs were approached *via* the congested HPB motif B.

Previously, we attempted a π-extension of CPPs using hexaphenylbenzene (HPB) as the general building block, which can be converted to hexa-*peri*-hexabenzocoronene (HBC) *via* oxidative cyclodehydrogenation. This concept has been widely employed for the bottom-up synthesis of 1D- and 2D-structures, such as graphene nanoribbons (GNR)s^12^ and nanographenes.^13^ To obtain π-lengthened CPPs (3D), HPB was embedded into a macrocycle to give a cyclic hexaphenylbenzene trimer ([3]CHPB) and its corresponding larger homologues.^14^ A further addition of phenyl rings afforded polyphenylene cylinders (PPC)s, such as **1**, following the parallelogram motif (frame A, Fig. 2).^15^ A complete cyclodehydrogenation could not be achieved. Thus, the macrocycles have been redesigned with a different connectivity, which is entirely based on the congested HPB motif (frame B, Fig. 2), where one bridging-phenylene moiety is shared by two hexaphenylbenzenes. This structural motif leads to a lower degree of rotational freedom of the attached phenyl groups, which is favorable for the desired cyclodehydrogenation.

Herein, we present the synthesis of PPCs that entirely consist of congested hexaphenylbenzene moieties. A congested cyclic hexaphenylbenzene hexamer ([6]CCHPB) **2** and its oval-shaped *C*_2_-symmetric congener **3** were synthesized *via* Suzuki coupling and subsequent reductive aromatization. The oxidative cyclodehydrogenation of both structures was investigated and a hitherto unprecedented degree of cyclodehydrogenation for PPC systems was achieved.

## Results and discussion

**Scheme 1:**
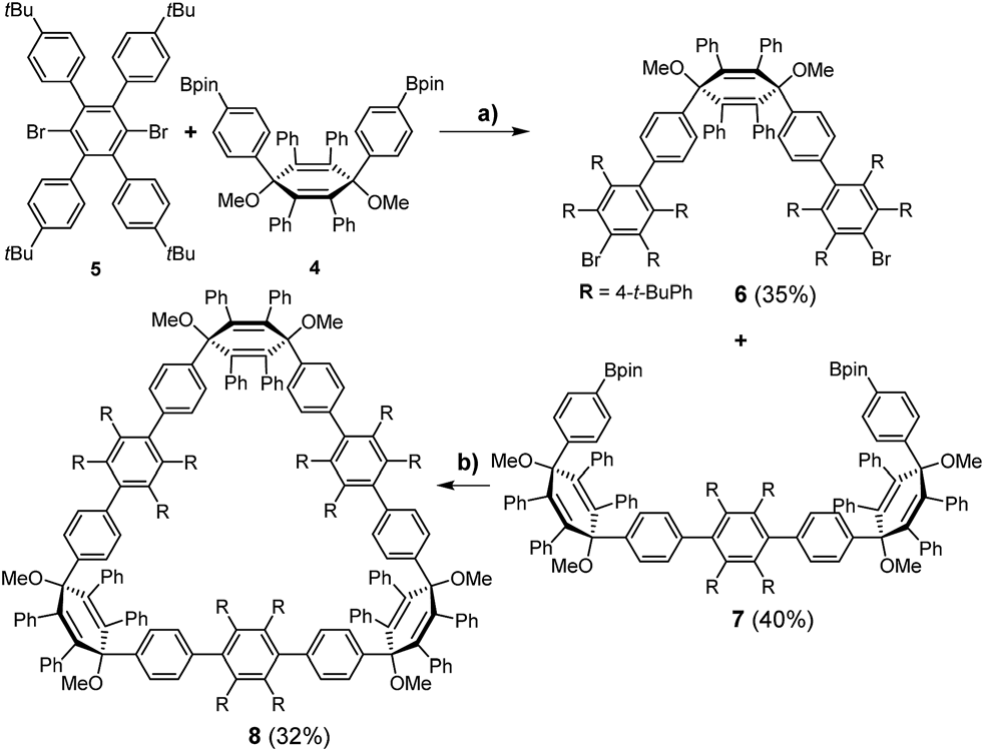
Synthesis of triangular macrocycle **8**. (a) Pd(PPh_3_)_4_, Cs_2_CO_3_, 100 °C, 2 d, (toluene/H_2_O); (b) Pd(PPh_3_)_4_, Cs_2_CO_3_, 100 °C, 2 d, (toluene/H_2_O).

For the synthesis of the new macrocycles [6]CCHPB **2** and **3** (Scheme 1), we applied a Suzuki-mediated cyclization introduced by Bertozzi and Jasti^3*a*^ together with a strategy, developed in our group, to convert structurally related, linear polyphenylene (PP) precursors into GNRs,^12b,16^ using oxidative cyclodehydrogenation.^17^ Therefore, the *para*-HPB diboronate, employed for linear PPs, was exchanged with the kinked diboronate 4. Two different precursors, **6** and **7**, were then foreseen to give the triangular macrocycle **8**, as the kink angles of the three V-shaped units in **6** and **7** add up to ∼210°.^14^ Tetraphenyl and tetrakis(4-*t*-butylphenyl) were introduced in an alternating fashion to provide solubility and render cyclodehydrogenation possible.

As shown in Scheme 1, the unique “di-kinked” diboronate **7** and its “mono-kinked” counterpart **6** are synthesized *via* Suzuki coupling. For each approach, a fourfold excess of either diboronate **4** or dibromide **5** was used. Dibromide **6** and diboronate **7** could be obtained in yields of 35%, and 40% respectively. The macrocyclization of these two building blocks was achieved by Suzuki coupling in diluted media (0.1 mmol) to give **8** in 32% yield; side products such as higher linear homologues could be separated by recycling GPC.

**Scheme 2:**
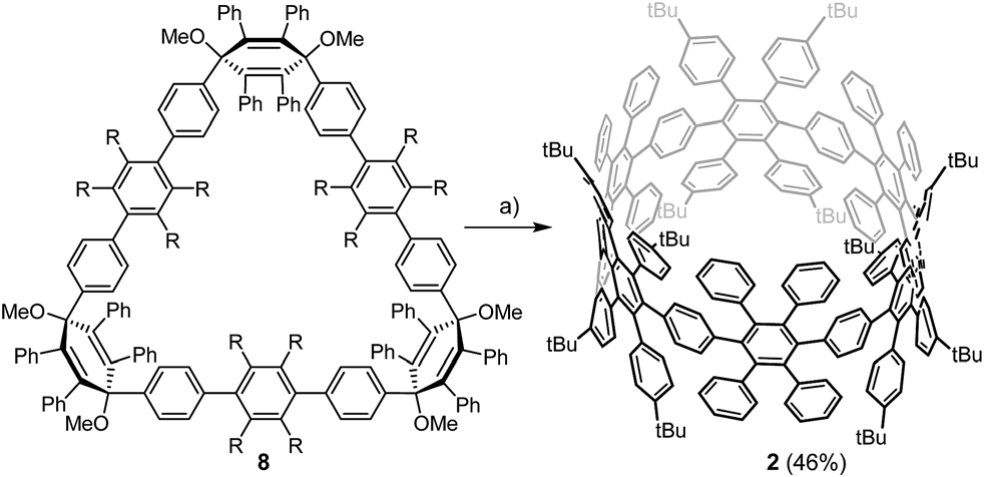
Synthesis of the [6]CCHPB **2**. (a) TiCl_4_, LiAlH_4_, 80 °C, 3 d (THF).

An initial demethoxylation of **8** was achieved under reductive conditions that had previously been applied in our group: low-valent titanium was generated *in situ* and reacted with the triangular macrocycle **8** to give **2** in 46% (Scheme 2). The applied conditions are very effective at inducing reductive aromatization, but require elevated temperatures (80 °C). To unambiguously characterize cyclohexamer **2**, 2D NMR experiments (Section 4.1, ESI†) were carried out. Single crystals of **2** were obtained after slow diffusion of MeCN into DCM. However, the obtained crystals did not yield sufficient reflections to resolve the structure.

**Fig. 3:**
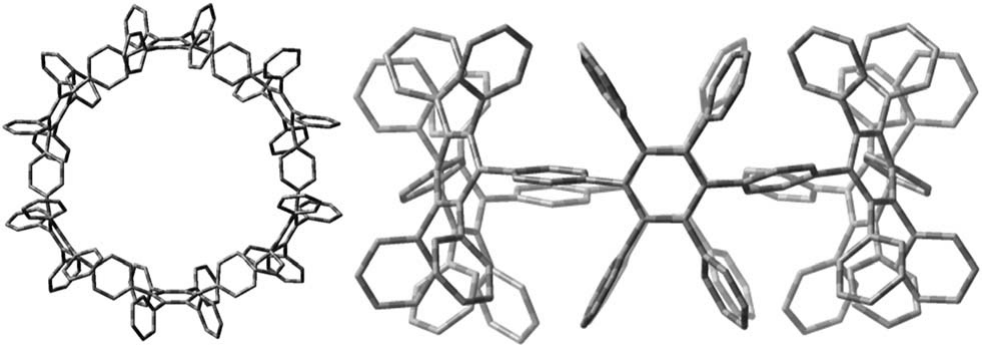
Optimized geometry of **2** top (left) and side (right) view, as calculated at the B3LYP/6-31G(d) level of theory. Hydrogen atoms are omitted for clarity.

Therefore, we performed density functional theory (DFT) calculations of **2** with the B3LYP hybrid functional^18^ and the 6-31G(d) Pople basis set.^19^ The fully optimized geometry revealed a torsional angle between neighboring phenylene units of about 90° (Fig. 3, right), in an alternating fashion within the CPP ring. Interestingly, in comparison to PPC **1** (Fig. 2, left), which is based on an odd membered CPP ring with an ellipsoidal structure, cyclohexamer **2** exhibits a circular-shaped structure.

**Scheme 3:**
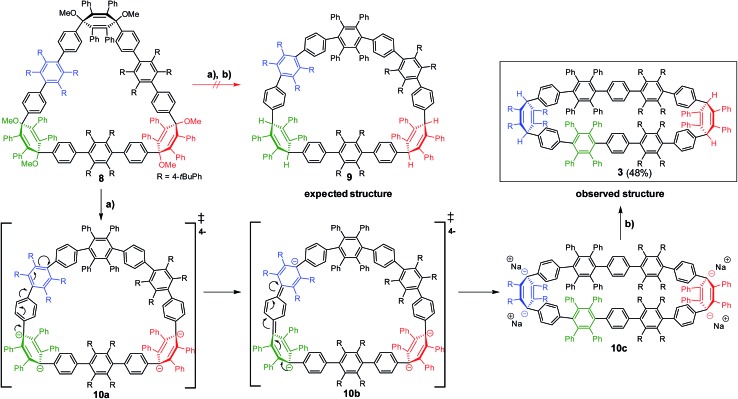
Proposed reaction mechanism for the synthesis of **3**. (a) NaNp, 1 h, –78 °C (THF); (b) –78 °C, H^+^.

In the second approach, a milder reduction of compound **8** with sodium naphthalenide (NaNp) (–78 °C *vs.* 80 °C) was attempted (Scheme 3). To our surprise, we did not obtain the expected [6]CCHPB **2**, but rather a macrocycle containing four additional hydrogen atoms (Scheme 3). This was first indicated by the blue color of the reaction solution, hinting at the formation of anionic species, which can stem from an overreduction of the cyclohexadiene moieties, as reported in the literature.^3*q*^ Therefore, the reaction was quenched with a proton source (MeOH) to trap the charged intermediate, while the use of iodine to quench the reaction or lowering the temperature did not afford any characterizable product. By mass spectrometry, we inferred that the obtained structure possesses four additional hydrogen atoms in comparison to [6]CCHPB **2**. We expected that the reductive aromatization had not overcome ring strain at temperatures of –78 °C, but rather came to a halt after *in situ* formation of a tetraanionic species, which was then preserved by protonation. For this structure, only one of the three 3,6-dimethoxycyclohexadienes was reduced, when applying the above described conditions to give one phenylene moiety instead of the expected three, as shown in Scheme 3. Astonishingly, single crystal X-ray crystallography (Fig. 5) revealed the formation of **3** instead of the expected structure **9**.

The formation of **3** can be explained by taking into account the mechanistic details of the aromatization: a stepwise two-electron reduction is proposed in the literature,^3*a*^ forming a radical under loss of one methoxy group. After the second one-electron transfer, a monoanion is formed, which leads to an expulsion of the remaining methoxy group under concomitant aromatization, affording a phenylene moiety from the previous 3,6-dimethoxycyclohexadiene. Based on the obtained product **3**, we propose a mechanism *via* a tetraanionic species with subsequent charge migration from the 3,6-bridging cyclohexadiene moiety to the tetrakis(*t*-butylphenyl)-substituted phenylene ring (Scheme 3), compound **10c**. In detail, the reductive aromatization follows the above mechanism for one of the three cyclohexadiene moieties. Reduction of the second and third cyclohexadiene units does not yield the neutral hydrocarbon **2** but proceeds instead to tetraanion **10c** (Scheme 3). Migration of the anionic charges in intermediate **10a** and **10b**, as shown by arrows in Scheme 3, gives the thermodynamically more favorable *C*_2_-symmetric tetraanion **10c**.^20^ Protonation “freezes” the anionically charged structure, leading to the oval-shaped compound **3**.

**Fig. 4:**
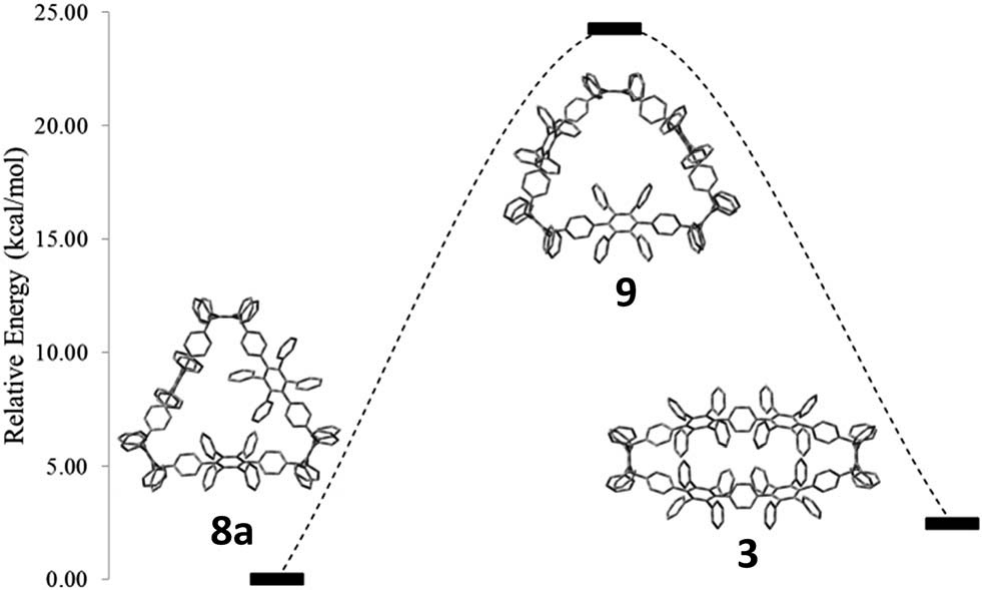
Comparison of strain energies of **8a**, **9**, and **3**, using DFT calculations at the B3LYP/6-31G(d) level of theory. For all structures, methoxy groups are substituted by hydrogen atoms. Hydrogen atoms are omitted for clarity.

To support this mechanistic hypothesis, we performed DFT calculations for the different macrocycles shown in Fig. 4. Interestingly, the tetraanion **10** was not found as a stable intermediate, but rather spontaneously evolved into the *C*_2_-symmetric tetraanion **10c**. On the other hand, the neutral counterpart of the anionic structures (namely **8a**, **9**, and **3**; for all structures the methoxy groups are substituted by hydrogen atoms to reduce computational cost) were obtained as stable structures. To account for the observed charge migration, we compared the relative strain energies of **3**, **8a**, and **9** (Fig. 4). The calculations reveal an increase of strain energy by ∼24.3 kcal mol^–1^ when going from **8a** to **9**. This originates from the presence of one additional phenylene moiety obtained after reductive aromatization of **8a**; hence a strain relief toward the starting material **8a** is not feasible. On the other hand, a thermodynamically more favorable geometry with a reduced relative strain energy of ∼2.5 kcal mol^–1^ can be reached through a charge migration along one phenylene unit from **10a** over **10b** to its protonated form **3**, as depicted in Scheme 3.

**Fig. 5:**
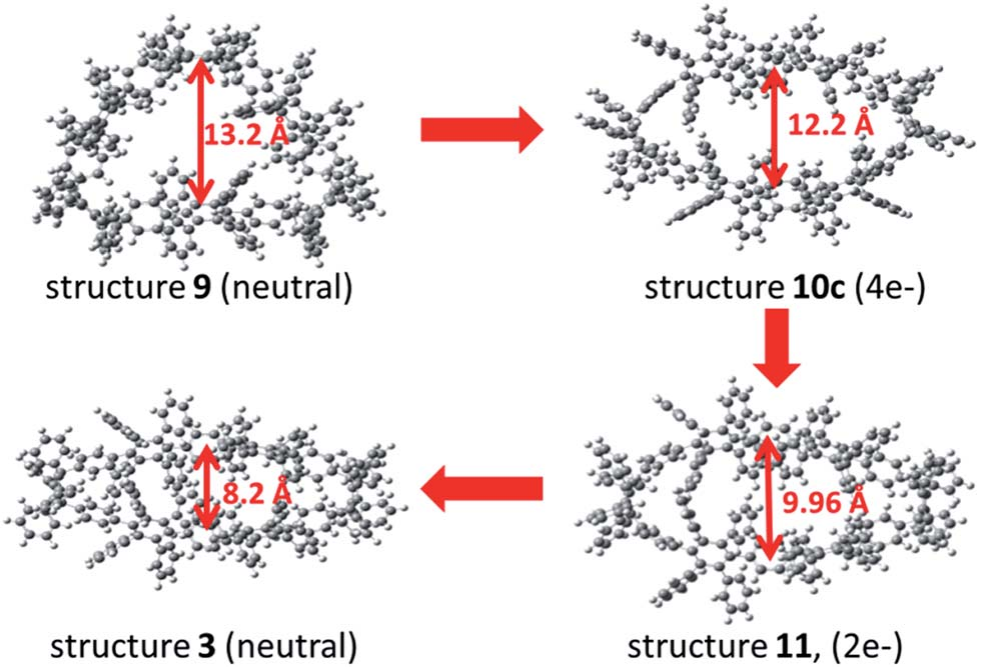
Geometric analysis of structures **9**, **10c**, **11** and **3** to understand the equilibrium structure of tetraanion **10c**, using DFT calculations at the B3LYP/6-31G(d) level of theory. Structure **11** is twice negatively charged at one cyclohexadiene moiety, whereas the other contains two hydrogen atoms, analogously to the other structures.

To support the postulated resonance form of the tetraanionic intermediate **3a**, the size of the holes of macrocycle **3** (neutral structure) and **10c** (tetraanionic intermediate) were studied. The computed geometry optimization suggests an increase of the oval opening from 8.2 Å to 12.2 Å, respectively (opening of the neutral structure **9** is 13 Å), which is shown in Fig. 5. Thus, the equilibrium structure of the tetraanionic intermediate **10c** is shifted toward **3** in comparison to **9**. To understand whether the strain release is accompanied by a redistribution of charge over the entire aromatic system, a charge analysis for **11** (2e^–^) and **10c** (4e^–^) was performed: the dianionic structure **11** is considered as a starting point and shows that the charges are mainly localized over the cyclohexadiene moieties where the hydrogen atoms have been removed. For **10c**, the calculations reveal that the main part of the charges (∼2e^–^) is strongly localized over the cyclohexadiene moieties of the macrocycle, while the remaining partial charges (∼2e^–^) are delocalized over the substituted phenyl rings of the belt (Fig. S16, ESI†), leading to an oval shaped macrocycle. These findings additionally corroborate the postulated form of the tetraanionic intermediate **10c** in opposition to **10a**. The proposed charge migration based on DFT calculations is also supported for a dianionic species after formation of one phenylene moiety (Fig. S10 and S15, ESI†). An over-reduction of [6]CCHPB **2** to give the tetraanion **10c**, which can be trapped by a proton source, has not been observed experimentally.

**Fig. 6:**
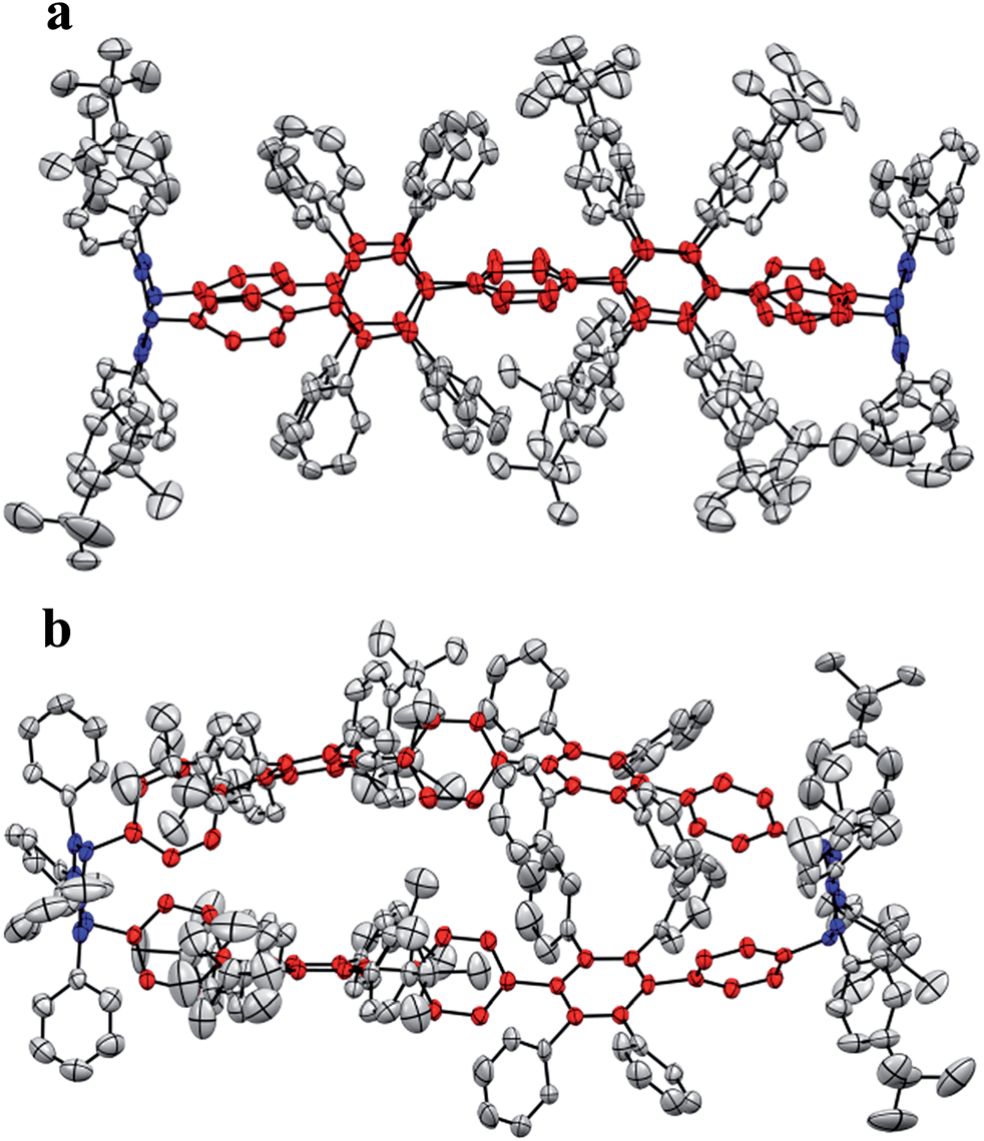
Crystal structure of **3**. ORTEP drawing at 50% probability level. All hydrogen atoms and solvent molecules are omitted for clarity. Phenylene moieties are labelled in red. 3,6-Bridging cyclohexa-1,4-dienes are labelled in blue. Front view (a) and top view (b).

Single crystals of **3** were obtained after slow vapor diffusion of MeCN into a solution of DCM. The crystal structure confirmed the oval-shaped nature (Fig. 6) of **3**. Pentaphenylene units (labeled in red) are bridged by two 3,6-substituted cyclohexa-1,4-dienes (labeled in blue). The pentaphenylene moieties are slightly bent (Fig. 6b) as the sp^3^-carbons cannot be fully torn out of plane due to angle constraints (Fig. 3, ESI†). Additionally, the crystal structure reveals the inherent design pattern: tetraphenyl and tetrakis-(4-*t*-butylphenyl) substituents are attached to the central ring in an alternating fashion.

As compounds **2** and **3** were designed in order to serve as direct precursors for a solution-based bottom-up synthesis of CNTs, we subjected both molecules to oxidative cyclodehydrogenation conditions using FeCl_3_ at room temperature.^15^ For **2**, a loss of 20 hydrogen atoms of a possible 96 was observed by MALDI-MS, at which the cyclodehydrogenation came to a halt, despite prolonged reactions times. In contrast to our previous results, here we could, for the first time, determine the degree of cyclodehydrogenation due to isotopically resolved mass peaks. For the barely strained *C*_2_-symmetric congener **3**, of the 96 hydrogen atoms to obtain a CNT a loss of 60 could be observed (see Scheme S3,† p. S92). The detailed analysis of the mass spectra hints at the formation of ribbon-like sidewall structures (Section 7.2, ESI†), rendering cyclodehydrogenation of the pentaphenylene sidewalls reasonable. However, since the structural analysis is based on mass spectrometry, well known side reactions for Scholl reactions, such as rearrangements, cannot be ruled out. Prolonged reaction times led to chloro-de-*t*-butylations; oxidative aromatization of the cyclohexadiene moieties was not observed, even with other reagents at elevated temperature (DDQ, 120 °C).

## Conclusions

In summary, we presented the bottom-up synthesis of a [12]CPP based nanobelt precursor [6]CCHPB **2** and its unexpected weakly-strained *C*_2_-symmetric congener **3**. Their structural features are based on congested hexaphenylbenzene units, *i.e.*, two hexaphenylbenzenes are bridged by one shared phenylene unit. The surprising synthesis of **3** occurred due to the use of the milder reducing agent NaNp, whereas low valent titanium was applied for the synthesis of the congested HPB **2**. We assume that two different products were obtained because of the high difference of thermal energy (Δ*T* 160 °C), giving rise to a kinetic control of the reaction. The mechanistic explanation for the astonishing charge migration during reductive aromatization of **3** was confirmed by DFT calculations. The inherent congested HPB structural feature leads to reduced rotational freedom for both macrocycles in comparison to the previously prepared PPCs such as **1**. Finally, we attempted oxidative cyclodehydrogenation which resulted in a loss of 20 H and 60 H for **2** and **3**, respectively. The higher loss for **3** may be assigned to the barely strained pentaphenylenes. The above discussed findings suggest that the oxidative cyclodehydrogenation runs more smoothly if (a) the congested HPB structural motif in comparison to the parallelogram approach is applied, as this mode of connectivity restricts rotation of the π-extended macrocycle, thus avoiding mismatched structural arrangements possible for **1** (parallelogram *vs.* trapezoid); and (b) a minimum of strain is present in the cyclic system, observed for **3** in comparison to **2**. Thus, the synthesis of such non-strained precursors which can be pre-cyclodehydrogenated close to the target structure and are then being converted to strained geometries could open up new concepts for a concise bottom-up synthesis of carbon nanobelts.

## References

[cit1] Dresselhaus M. S., Dresselhaus G., Charlier J. C., Hernández E. (2004). Philos. Trans. R. Soc. London.

[cit2] Louie S., in Carbon Nanotubes, ed. M. Dresselhaus, G. Dresselhaus and P. Avouris, Springer Berlin Heidelberg, 2001, vol. 80, ch. 6, pp. 113–145.

[cit3] Jasti R., Bhattacharjee J., Neaton J. B., Bertozzi C. R. (2008). J. Am. Chem. Soc..

[cit4] Wu Y.-T., Siegel J. S. (2006). Chem. Rev..

[cit5] Omachi H., Nakayama T., Takahashi E., Segawa Y., Itami K. (2013). Nat. Chem..

[cit6] Yu X., Zhang J., Choi W., Choi J.-Y., Kim J. M., Gan L., Liu Z. (2010). Nano Lett..

[cit7] (b) Petrukhina M. A. and Scott L. T., Fragments of Fullerenes and Carbon Nanotubes, John Wiley & Sons, Inc., Hoboken, NJ, USA, 2011.

[cit8] Omachi H., Segawa Y., Itami K. (2011). Org. Lett..

[cit9] He Z., Xu X., Zheng X., Ming T., Miao Q. (2013). Chem. Sci..

[cit10] Yagi A., Segawa Y., Itami K. (2012). J. Am. Chem. Soc..

[cit11] Ishii Y., Matsuura S., Segawa Y., Itami K. (2014). Org. Lett..

[cit12] Iyer V. S., Yoshimura K., Enkelmann V., Epsch R., Rabe J. P., Müllen K. (1998). Angew. Chem., Int. Ed..

[cit13] Iyer V. S., Wehmeier M., Brand J. D., Keegstra M. A., Müllen K. (1997). Angew. Chem., Int. Ed..

[cit14] Nishiuchi T., Feng X., Enkelmann V., Wagner M., Müllen K. (2012). Chem.–Eur. J..

[cit15] Golling F. E., Quernheim M., Wagner M., Nishiuchi T., Müllen K. (2014). Angew. Chem., Int. Ed..

[cit16] Müller M., Iyer V. S., Kübel C., Enkelmann V., Müllen K. (1997). Angew. Chem..

[cit17] Sarhan A. A. O., Bolm C. (2009). Chem. Soc. Rev..

[cit18] Stephens P. J., Devlin F. J., Chabalowski C. F., Frisch M. J. (1994). J. Phys. Chem..

[cit19] Rassolov V. A., Ratner M. A., Pople J. A., Redfern P. C., Curtiss L. A. (2001). J. Comput. Chem..

[cit20] Sekiguchi A., Ebata K., Kabuto C., Sakurai H. (1991). J. Am. Chem. Soc..

