# Supplementary material for: π-extended [12]cycloparaphenylenes: from a hexaphenylbenzene cyclohexamer to its unexpected C 2-symmetric congener
Source: Chem Sci. 2015 Sep 8;6(12):7072–8. doi: 10.1039/c5sc02547h (PMC5510010; doi:10.1039/c5sc02547h)
Supplement: Supplementary file 1 [file SC-006-C5SC02547H-s001.pdf]

Electronic Supplementary Material (ESI) for Chemical Science.
This journal is © The Royal Society of Chemistry 2015

Supporting Information

π-Extended [12]Cycloparaphenylenes: from a Hexaphenylbenzene Cyclohexamer to its Unexpected C_2-symmetric Congener

Florian E. Golling[†,‡], *Silvio Osella*[§], *Martin Quernheim*[†], *Manfred Wagner*[†], *David Beljonne*[§], *Klaus Müllen*[†,*]

[†] Max-Planck-Institut für Polymerforschung, Ackermannweg 10, 55128 Mainz (Germany)

[‡] Graduate School Materials Science in Mainz, Staudinger Weg 9, 55128 Mainz (Germany)

[§] Chimie des Matériaux Nouveaux & Centre d'Innovation et de Recherche en Matériaux Polymères, Université de Mons- UMONS/Materia Nova, Place du Parc 20, 7000 Mons (Belgium)

Contents

1. **General Information** S2
2. **Synthesis** S4
3. **X-Ray Crystallographic Analysis** S13
4. **2D-NMR Spectra** S16
5. **Computational Results** S22
6. **NMR Spectra** S82
7. **MALDI-MS spectra** S91
8. **Optical and Electronic Properties** S103
9. **Literature** S105

1. General Information

Unless otherwise stated, the commercially available reagents and dry solvents were used without further purification. The reactions were performed using standard vacuum-line and Schlenk techniques, work-up and purification of all compounds was performed under air and with reagent-grade solvents. Column chromatography was done with silica gel (particle size 0.063-0.200 mm from Macherey-Nagel) and silica coated aluminum sheets with fluorescence indicator from Macherey-Nagel were used for thin layer chromatography. Preparative thin layer chromatography was done with PLC silica gel 60, F254, 2 mm sheets on glass from Merck. Melting points were determined on a Büchi hot stage apparatus. The ^{1}H-NMR and ^{13}C-NMR spectra were recorded on a Bruker AVANCE 250, Bruker AVANCE 300, Bruker AVANCE 500 and Bruker AVANCE 700 spectrometer in the listed deuterated solvents. Trimethylsilane (δ 0.00 ppm) or the deuterated solvent was used as an internal standard. Field desorption (FD) mass spectra were obtained on a VG Instruments ZAB 2 SE-FPD. MALDITOF mass spectra were recorded on a Bruker Reflex II-TOF spectrometer using trans-2-[3-(4-tert-butylphenyl)-2-methyl-2-propenylidene]-malononitrile (DCTB; Aldrich, >99%) as matrix. High resolution MALDI mass spectrometry measurements were performed on a Solarix ESI-/MALDI-ICR (9.4T) system (Bruker Daltonics, Germany), with a SmartBeam laser II. The system was internally calibrated in positive mode using sodium trifluoroacetate (Fluka, >99%) or sodium perfluoroheptanoate (Fluka, >99%) on quadratic calibration mode. A total of 10-400 shots were accumulated for each mass spectrum. The results were calculated using Data Analysis software (Bruker Daltonics, Germany). High-performance liquid chromatography (HPLC) was performed on a HPLC facility from Shimadzu (LC-20AD), using a GPC column from JAI Co., Ltd. Packed with highly cross-linked polystyrene/divinylbenzene.

1.1. *General Information for the 2D NMR experiments*

^{1}H-NMR (700 MHz) and ^{13}C-NMR (176 MHz) measurements were executed on Bruker Avance III 700 NMR spectrometers with a 5 mm QXI probe endowed with a z-gradient. The spectra were received with $\pi/2$-pulse lengths of 13.8 µs (^{1}H) and 16 µs (^{13}C) and a sweep width of 10500 Hz (15 ppm) for ^{1}H and 35000 Hz (200 ppm) for ^{13}C. A relaxation delay of 2s was used for both nuclei. The temperature was held at 298.3 K and calibrated with a standard ^{1}H methanol NMR sample. The control of the temperature was realized with a VTU (variable temperature unit) and an accuracy of +/- 0.1K, which was checked with the standard Bruker Topspin 3.2 software. A standard proton spectrum was recorded with 1024 transients. The 2D ^{1}H,^{13}C-HSQC (heteronuclear single quantum correlations via double inept transfer and phase sensitive using Echo/Antiecho-TPPI gradient selection with decoupling during acquisition) experiment was run with 4096 points in f2 and 512 points in f1 dimension and an averaged ^{1}J-CH coupling constant of 145Hz. Before Fourier transformation, the data were zero filled to 1024 points in f1 and multiplied by a window function (q-sine bell or sine bell) in both dimensions. The assignment of the protons was realized with a 2D-^{1}H,^{1}H NOESY (nuclear overhauser enhancement spectroscopy) and 2D-^{1}H,^{1}H COSY. The used mixing time in this experiment was kept at 250 ms. The spectroscopic widths of the homo-nuclear 2D COSY and NOESY experiments were typically 10000 Hz in both dimensions (f1 and f2) and the relaxation delay was 1.3s.

2. Synthesis

2.1 *Synthesis of 3',6'-dibromo-4,4''-di-tert-butyl-4',5'-bis(4-(tert-butyl)phenyl)-1,1':2',1''-terphenyl* (**5**)[1]

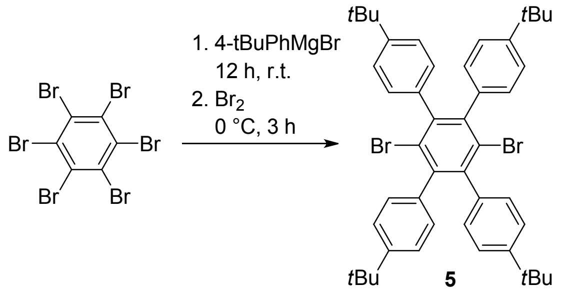

5

C_6Br_6
m.w.: 551,49 g/mol

$C_{46}H_{52}Br_2$
m.w.: 764.71 g/mol

Hexabromobenzene (8.27 g, 15 mmol, 1 eq) was placed into a *Schlenk* flask and 4-*tert*-butylphenylmagnesium bromide (300 mL, 150 mmol, 10 eq.) was added and the reaction was stirred for 12 h. Then, the reaction was cooled down to 0 °C and slowly quenched with bromine. After quenching the excess amount of bromine with aq. $Na_2S_2O_3$, the crude product was extracted with Et_2O and the combined fractions were filtered through a fritted funnel. The filter cake was washed with water, EtOH, hexane and toluene to give the title compound **5**. The solvent of the filtrate was removed under *vacuo*. The solid residue was washed analogously to the filter cake. The reaction afforded **5** (5.1 g, 6.68 mmol 37%) as a white solid.

^{1}H NMR (300 MHz, THF) δ [ppm] = 7.14 (d, *J*=8.3, 8H), 6.94 (d, *J*=8.3, 8H), 1.21 (s, 36H).
^{13}C NMR (75 MHz, THF) δ [ppm] = 149.9, 144.5, 139.2, 130.5, 125.4, 124.6, 34.8, 31.4.
MS (FD, 8 kV) (*m/z*): obsvd. for $C_{46}H_{52}Br_2$ [M^+] = found 763.9.(calcd. 764.2)
Elemental Analysis for $C_{46}H_{52}Br_2$: found C, 71.46 (calcd. C, 72.25); H, 6.46 (H, 6.85).
Melting Point: > 300 °C.
X-ray crystal structure: CCDC 1033268. These data can be obtained from The Cambridge Crystallographic Data Centre via *https://summary.ccdc.cam.ac.uk/structure-summary-form*.

2.2 *Synthesis of (1's,4's)-4-bromo-4'-(4-bromophenyl)-3',5',6'-triphenyl-1',4'-dihydro-[1,1':2',1''-terphenyl]-1',4'-diol* (**11**)

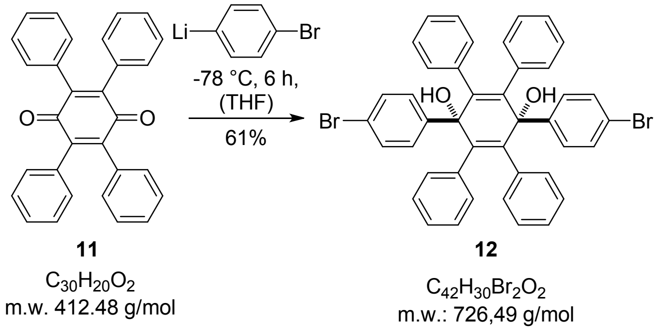

1,4-dibromobenzene (9.26 g, 39.3 mmol) was dissolved in THF (200 mL) and cooled down to – 78 °C. After addition of *n*-BuLi, the reaction mixture was stirred for 30 min. Then, a suspension of 2,3,5,6-Tetrakis(phenyl)benzoquinone[2] **11** (2.70 g, 6.54 mmol) in THF (30 mL) was slowly added. The reaction was stirred for 2 h, warmed to r.t. and stirred overnight. The crude product was purified by column chromatography (6:4 DCM/hexane → DCM → 8:2 hexane/EtOAc) to give **12** (2.9 g, 3.99 mmol, 61%) as a white solid.

^{1}H NMR (300 MHz, CD_2Cl_2) ^{1}H NMR (300 MHz, CD_2Cl_2) δ [ppm] =7.41 (d, *J* = 8.8 Hz, 4H), 7.29 (d, *J* = 8.5 Hz, 4H), 7.01 – 6.86 (m, 12H), 6.87 – 6.75 (m, 8H), 2.66 (s, 2H).

^{13}C NMR (75 MHz, CD_2Cl_2) δ [ppm] = 142.06, 141.05, 137.47, 131.85, 131.19, 129.42, 127.26, 126.82, 121.67, 75.26.

MS (FD, 8 kV) (*m/z*): obsd. for $C_{42}H_{30}Br_2O_2$ $[M]^+$: 725.4 (726.1)

Elemental Analysis: not measured due to rapid decomposition of the sample.

Mp: decomposes above 40 °C.

2.3 Synthesis of (1's,4's)-4-bromo-4'-(4-bromophenyl)-1',4'-dimethoxy-3',5',6'-triphenyl-1',4'-dihydro-1,1':2',1''-terphenyl (**13**)

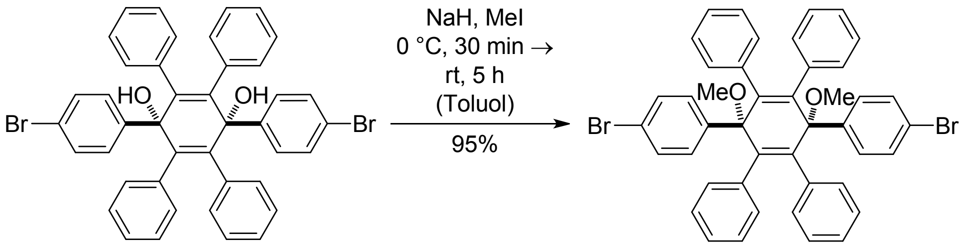

12
$C_{42}H_{30}Br_2O_2$
m.w.: 726,49 g/mol

13
$C_{44}H_{34}Br_2O_2$
m.w.: 754,55 g/mol

12 (2.9 g, 3.99 mmol) was dissolved in 30 mL THF and cooled to 0 °C. After 30 min, NaH (0.48 mg, 12.0 mmol) (60% mineral oil) was added and the solution was stirred for another 30 min until MeI (3.4 g, 24.0 mmol) was added. The reaction was stirred at 0 °C for 2 h, then warmed to r.t. and stirred overnight. After quenching with methanol, water was added and the reaction mixture was extracted with Et_2O (3 x) and the combined organic fractions were dried over $MgSO_4$. The crude product was filtered through a short pad of silica (6:4 → hexane/DCM) to give **13** (2.85 g, 3.78 mmol) as a white solid (95%).

^{1}H NMR (500 MHz, $C_2D_2Cl_4$,) δ [ppm] = 7.44 (d, J = 8.5 Hz, 4H), 7.35 (d, J = 8.7 Hz, 4H), 6.91 – 6.80 (m, 12H), 6.72 (dd, J = 8.1, 1.4 Hz, 8H), 3.89 (s, 6H).

^{13}C NMR (126 MHz, $C_2D_2Cl_4$,) δ [ppm] = 143.15, 142.55, 138.67, 131.64, 130.87, 130.18, 127.17, 126.71, 121.38, 81.77, 52.70.

MS (FD, 8 kV) (m/z): obsd. for $C_{44}H_{34}Br_2O_2$ $[M]^+$: 755.4 (calcd. 754.1).

Elemental Analysis for $C_{44}H_{34}Br_2O_2$: found C, 70.47 (calcd. C, 70.04); H, 4.54 (H, 4.30).

Mp: 292 °C.

2.4 *Synthesis of 2,2'-((1's,4's)-1',4'-dimethoxy-2',3',5',6'-tetraphenyl-1',4'-dihydro-[1,1':4',1''-terphenyl]-4,4''-diyl)bis(4,4,5,5-tetramethyl-1,3,2-dioxaborolane)* (4)

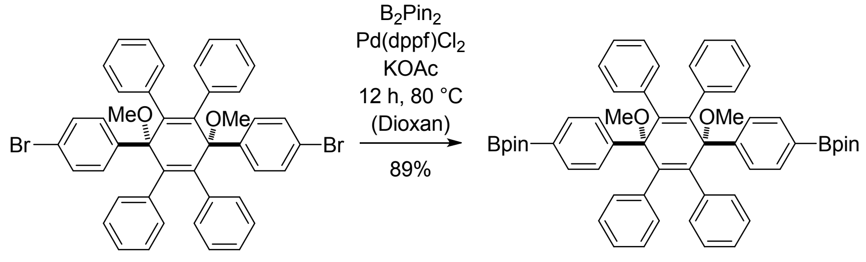

13 | **4**
$C_{44}H_{34}Br_2O_2$ m.w.: 754.55 g/mol | $C_{56}H_{58}B_2O_6$ m.w.: 848.68 g/mol

13 (1.3 g, 1.72 mmol, 1 eq.), B_2Pin_2 (3.78 g, 5.17 mmol, 3 eq.), KOAc (3.38 g, 34.4 mmol, 20 eq.), and Pd(dppf)Cl_2[3] (76 mg, 0.10 mmol, 10mol%) were dissolved in dioxane (15 mL). The reaction was heated to 80 °C and stirred for 24 h. After completion, the crude product was extracted with Et_2O (3x) and the combined organic fractions were dried over $MgSO_4$. After removal of solvent until complete dryness, the crude product was recrystallized from hexane/MeOH to give **4** (1.30 g, 1.53 mmol, 89%) as a white solid.

^{1}H NMR (500 MHz, $C_2D_2Cl_4$, 373 K) δ[ppm] = 7.63 (s, 1H), 6.90 – 6.77 (m, 1H), 6.72 (d, *J* = 6.9 Hz, 1H), 3.91 (s, 1H), 1.36 (s, 1H).

^{13}C NMR (126 MHz, $C_2D_2Cl_4$, 373 K) δ [ppm] = 145.66, 142.59, 138.55, 133.56, 131.22, 127.20, 126.35, 125.79, 83.50, 81.70, 52.01, 24.77.

MS (FD, 8 kV) (*m/z*): obsd. for $C_{56}H_{58}B_2O_6$ $[M]^+$ = 848.0 (calcd. 848.4).

Elemental Analysis for $C_{56}H_{58}B_2O_6$: found C, 80.31 (calcd. C, 79.25); H, 6.89 (H, 7.35).*

*Solvent molecules were still encapsulated with the crystals, despite prolonged drying.

Mp: 300 °C.

2.5 *Synthesis of dikinked diboronate* ***6***

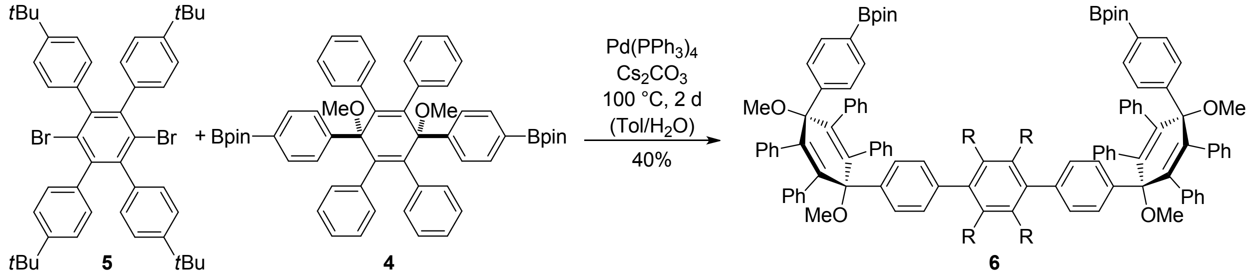

$C_{46}H_{52}Br_2$ MW: 764.71 g/mol | $C_{56}H_{58}B_2O_6$ m.w.: 848.68 g/mol | $C_{146}H_{144}B_2O_8$ m.w.: 2048,32 g/mol

5 (110 mg, 0.14 mmol, 1 eq.), **4** (732 mg, 0.86 mmol, 6 eq.) and Cs_2CO_3 (5.63g, 17.3 mmol, 20 eq.) (3 M) were added to a 25 mL Schlenk tube. After addition of toluene (6 mL), the reaction mixture was heated to 100 °C and stirred for 2 d. The crude product was extracted with DCM (3x) and the combined fractions were dried over $MgSO_4$. The crude product was filtered through a short pad of celite and washed with DCM. Finally, the crude product was fully purified by preparative GPC (eluent THF) to give **6** (114 mg, 56.0 µmol, 40%).

^{1}H NMR (500 MHz, $C_2D_2Cl_4$) δ = 7.43 (d, *J* = 8.0 Hz, 4H), 7.33 (d, *J* = 7.9 Hz, 4H), 6.88 (t, *J* = 8.2 Hz, 14H), 6.82 (dd, *J* = 9.5, 6.8 Hz, 14H), 6.79 – 6.74 (m, 10H), 6.69 (dd, *J* = 17.2, 7.6 Hz, 16H), 6.57 (d, *J* = 7.4 Hz, 8H), 3.88 (s, 6H), 3.66 (s, 6H), 1.34 (s, 24H), 1.13 (s, 36H).

^{13}C NMR (126 MHz, $C_2D_2Cl_4$) δ 148.10, 146.12, 143.39, 142.20, 141.25, 140.36, 139.45, 139.36, 139.20, 138.90, 138.68, 134.07, 132.13, 131.78, 131.35, 127.51, 126.86, 126.73, 126.23, 126.16, 123.46, 84.02, 82.20, 82.15, 74.62, 74.40, 74.18, 52.52, 52.46, 34.43, 31.77, 25.37.

HR-MS (MALDI) *m/z* obsv for $C_{146}H_{144}B_2O_8$ = 2048.1080 (calcd. 2048.1098).

MP: 290 °C

2.6 Synthesis of monokinked dibromide **7**

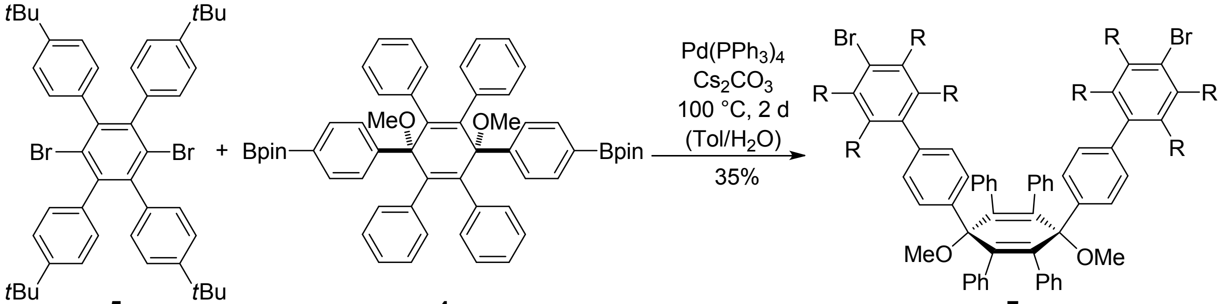

5 — $C_{46}H_{52}Br_2$, MW: 764.71 g/mol

4 — $C_{56}H_{58}B_2O_6$, m.w.: 848.68 g/mol

7 **R = 4-*t*BuPh** — $C_{136}H_{138}Br_2O_2$, m.w.: 1964,36 g/mol

5 (100 mg, 0.14 mmol, 4 eq.), **4** (28 mg, 0.033 mmol,1 eq.) and Cs_2CO_3 (215 mg ,0,66 mmol, 20eq.) (3 M) were added to a 25 mL Schlenk tube. After addition of toluene (8 mL), the reaction mixture was heated to 100 °C and stirred for 2 d. The crude product was extracted with DCM (3x) and the combined fractions were dried over $MgSO_4$. The crude product was filtered through a short pad of celite and washed with DCM. Finally, the crude product was fully purified by preparative GPC (eluent THF) to give **7** (42 mg, 21.4 µmol, 65%).

^{1}H NMR (500 MHz, $C_2D_2Cl_4$, 373 K) δ 7.16 (d, *J* = 8.6 Hz, 8H), 7.01 (d, *J* = 8.4 Hz, 8H), 6.90 (d, *J* = 8.6 Hz, 8H), 6.85 (t, *J* = 7.4 Hz, 4H), 6.76 (t, *J* = 7.7 Hz, 8H), 6.72 (d, *J* = 7.2 Hz, 8H), 6.59 (m, *J* = 11.2, 8.8 Hz, 8H), 6.47 (d, *J* = 7.4 Hz, 8H).

^{13}**C NMR** (126 MHz, $C_2D_2Cl_4$, 373 K) δ 149.53, 148.61, 142.90, 142.61, 142.40, 139.81, 139.51, 139.42, 138.89, 138.69, 137.88, 132.03, 131.52, 130.90, 130.76, 127.03, 126.60, 126.14, 125.05, 124.08, 123.50, 82.16, 52.37, 34.68, 34.47, 31.77, 31.65.

HR-MS (MALDI) (*m/z*) = obsv. for $C_{136}H_{138}Br_2O_2$ [M$^+$]: 1963.9097 (calcd. 1963.9086).

Mp: > 300 °C.

*2.7 Synthesis of triangular macrocycle **8***

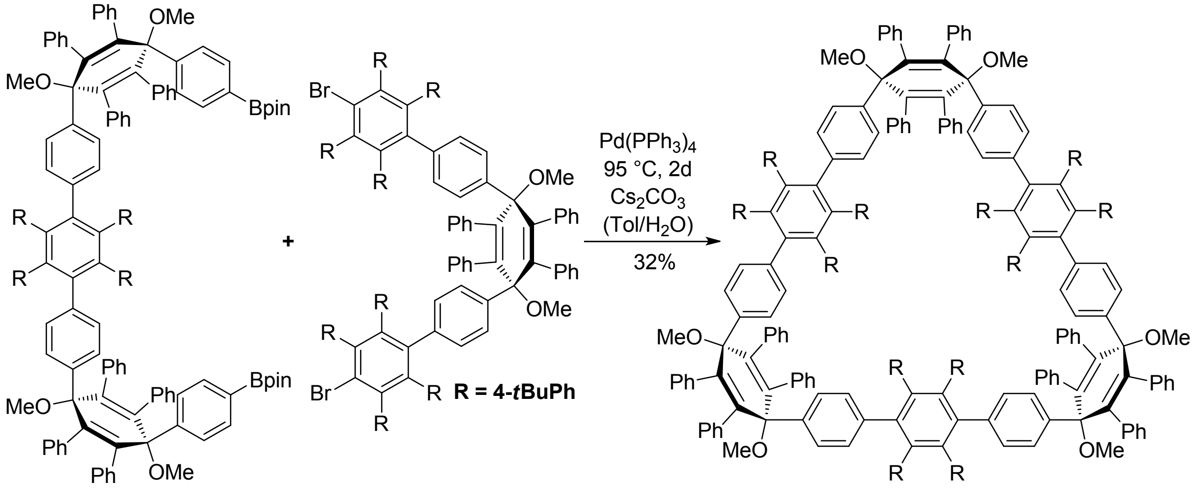

6 $C_{146}H_{144}B_2O_8$ m.w.: 2048,32 g/mol

7 $C_{136}H_{138}Br_2O_2$ m.w.: 1964,36 g/mol

8 $C_{270}H_{258}O_6$ m.w.: 3598,93 g/mol

Dikinked diboronate **6** (18.2 mg, 8.9 μmol, 1 eq.), monokinded dibromide (17.5 mgf , 8.9 μmol, 1 eq.) and Cs_2CO_3 (116 mg, 0.36 mmol, 40 eq.) (3 M) were added to a 25 mL Schlenk tube. After addition of toluene (5 mL, 2 mM), the reaction mixture was heated to 95 °C and stirred for 2 d. The crude product was extracted with DCM (3x) and the combined fractions were dried over $MgSO_4$. The crude product was filtered through a short pad of celite and washed with DCM. The crude product was purified by recycling GPC (eluent chloroforom) to give **8** (10.2 mg, 2.85 μmol, 32%).

^{1}**H NMR** (700 MHz, $C_2D_2Cl_4$, 333 °C) δ ^{1}H NMR (700 MHz, $C_2D_2Cl_4$) δ 7.21 (d, *J* = 8.6 Hz, 6H), 7.09 (d, *J* = 9.0 Hz, 6H), 6.86 (d, *J* = 7.6 Hz, 24H), 6.81 (s, 24H), 6.75 (t, *J* = 7.3 Hz, 14H), 6.72 (d, *J* = 8.2 Hz, 6H), 6.61 (t, *J* = 7.6 Hz, 24H), 6.38 (d, *J* = 8.1 Hz, 6H), 6.34 (d, *J* = 7.8 Hz, 24H), 3.52 (s, 28H), 1.04 (s, 108H).

^{13}**C NMR** (176 MHz, $C_2D_2Cl_4$, 333 °C) δ ^{13}C NMR (176 MHz, $C_2D_2Cl_4$) δ 147.24, 141.82, 140.91, 137.96, 133.28, 131.59, 131.23, 126.40, 125.47, 122.98, 31.48.*
*not all quarternary carbons observed.

2D-NMR: ^{1}H-^{1}H-COSY, NOSY (see *section 4.2*)

HR-MS (MALDI) (*m/z*) = obsv. for $C_{270}H_{258}O_6$ [M^+]: 3598.0038 (calcd. 3598.9946)
Mp: > 300 °C

2.8 Congested Cyclic Hexaphenylbenzene Hexamer **2**

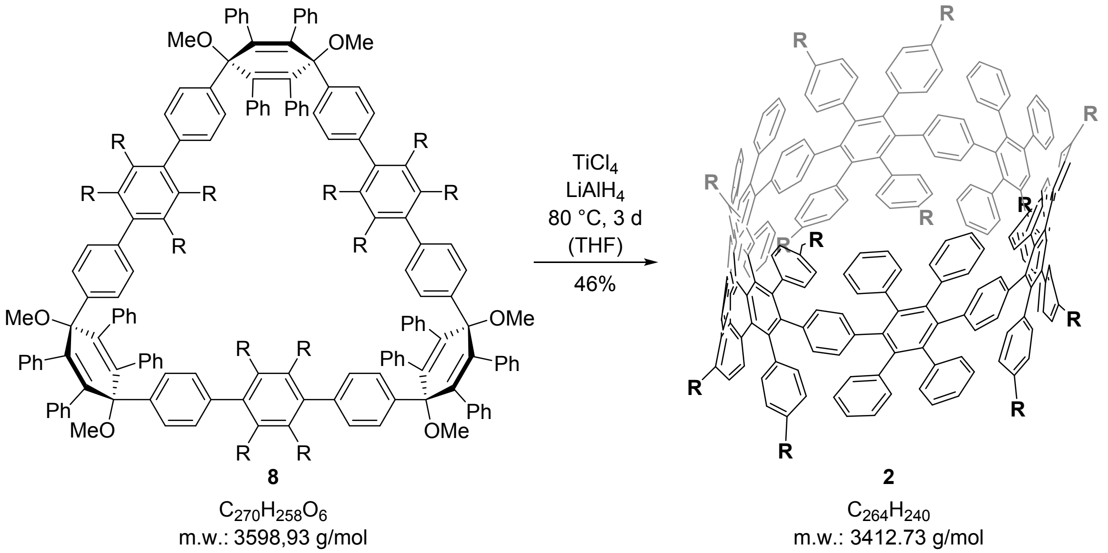

To TiCl4 (0.108 ml, 1.00 mmol, 600 eq.) was added THF (20 ml) at 0 °C under argon atmosphere and stirred for 30 min at ambient temperature. After cooling to 0 °C again, a solution of $LiAlH_4$ in THF (2.00 ml, 2.0 M, 4.00 mmol, 2400 eq) was added and stirred for 1h at 80 °C to generate low-valent titanium. A solution of **8** (6 mg, 1.66 µmol, 1 eq.) in THF (3 ml) was added and stirred for 3 d at same temperature under absence of light. After cooling to 0 °C, the reaction was quenched by water and 2M hydrochloric acid (Caution! Excess amount of unreacted $LiAlH_4$ can explosively lead to the formation of hydrogen gas upon addition of water. Therefore, at first, large glass adaptor or glass trap should be equipped at the top of the reactor. Additionally, several drops of water were slowly added to the reaction), and extracted with DCM. After filtration over celite, the organic layer was washed with water and brine, and dried over Na_2SO_4. After removing the solvent in *vacuo*, the crude product was subjected to preparative GPC (THF) to give **2** (2.6 mg, 76.2 µmol, 46%).

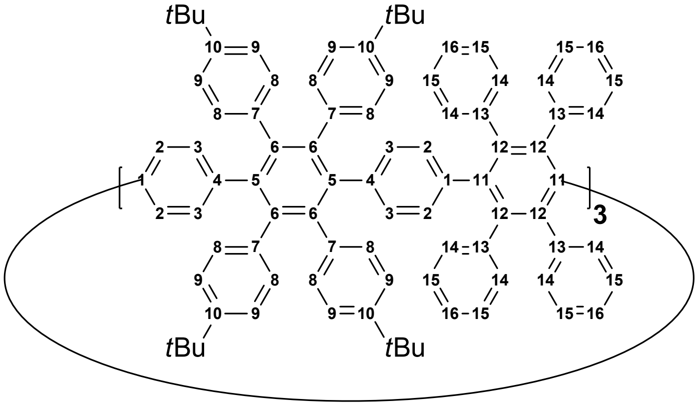

¹H NMR (500 MHz, $C_2D_2Cl_4$) δ 6.75 (t, 4H, C-16), 6.71 (d, *J* = 7.2 Hz, 8H, C-9), 6.65 (t, *J* = 6.7 Hz, 8H, C-15), 6.56 (d, *J* = 7.1 Hz, 8H, C-14), 6.44 (d, *J* = 7.3 Hz, 8H, C-8), 6.36 (d, *J* = 7.3 Hz, 4H, C-3), 6.22 (d, *J* = 7.2 Hz, 4H, C-2), 1.11 (s, 36H, $C((CH_3)_3)$).

¹³C NMR (126 MHz, $C_2D_2Cl_4$) δ 147.4 (C-7/C-6), 141.2 (C-1/11), 140.2 (C-7/C-6), 139.9 (C-4/5), 139.9 (C-13), 138.5 (C-1/11), 138.2 (C-4/5), 137.1 (C-10), 131.9 (C-14), 131.7 (C-16), 131.4 (C-8), 130.5 (C-3), 130.1 (C-2), 126.6 (C-15), 125.1 (C-12), 123.2 (C-9), 34.24 ($(CH_3)_3C$), 31.5 ($(CH_3)_3C$)

2D-NMR: see *section 4.1*

HR-MS (MALDI) (*m/z*) = obsv. for $C_{264}H_{240}$ [M^+]: 3411.8796 (calcd. 3411.8842).

Mp: > 300 °C

2.9. Synthesis of a C_2 -symmetric Cyclohexadiene Macrocycle **3**

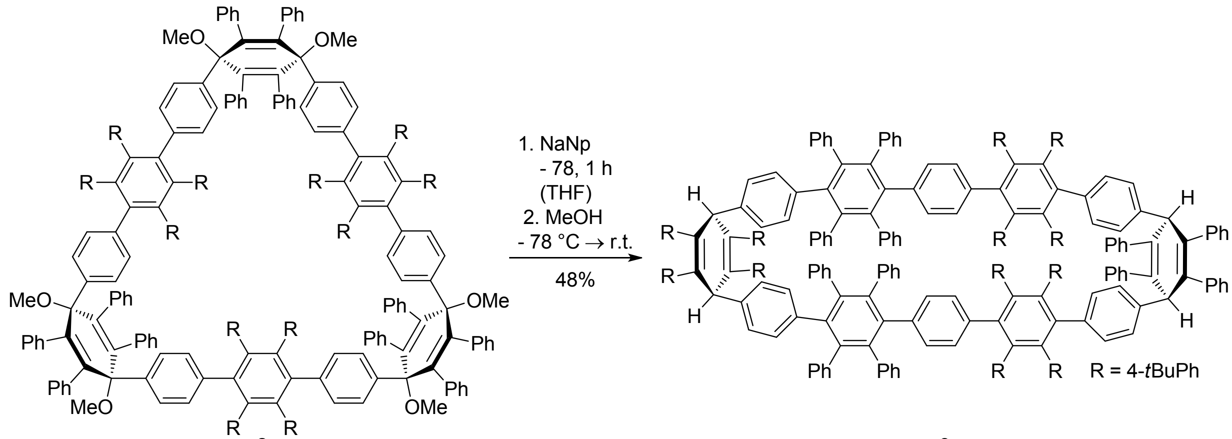

8 $C_{270}H_{258}O_6$ m.w.: 3598,93 g/mol

3 $C_{264}H_{244}$ m.w.: 3416.76 g/mol

8 (4 mg, 1.1 µmol, 1 eq.) were dissolved in THF (2 mL) and cooled to – 78 °C. Sodium napthalide (0.06 mL, 55.0 µmol, 50 eq.) in THF (1 M) was slowly added. Upon addition, the reaction color turned blue. The reaction solution was stirred at – 78 °C for 1 h and MeOH

(0.5 mL) was added. The colorless reaction solution was warmed to r.t.. After addition of water, the crude product was extracted with DCM (3x) and the combined fraction were dried over Na_2SO_4. The solvent was removed under *vacuo* and the product was purified by preparative GPC (THF). **3** (1.8 mg, 0.53 µmol, 48%) was obtained as a white solid.

HR-MS (MALDI) (*m/z*) = obsv. for $C_{264}H_{244}$ [M^+]: 3415.9074 (calcd. 3415.9154).
X-ray crystal structure: CCDC 1033267. These data can be obtained from The Cambridge Crystallographic Data Centre via *https://summary.ccdc.cam.ac.uk/structure-summary-form*.

Mp: > 300 °C

2.10 Oxidative Cyclodehydrogenation

General Reaction Conditions:

To a solution of **2** or **3** (1.00 µmol) in dichloromethane (5 ml) was added a solution of $FeCl_3$ (0.180 mmol) in nitromethane (1.5 ml). The mixture was reacted 1 d to 2 d under argon bubbling. Then, the reaction was quenched by MeOH and extracted with dichloromethane. The organic layer was washed with water and brine, and dried over $MgSO_4$. After removing solvent vacuum, the crude material was subjected to preparativeTLC. The isolated material was used for MALDI TOF-MS analysis and UV-vis, fluorescence measurements.

2.10 Control experiments investigating the overreduction of **8** using sodium napthalenide

Experiments were performed according to literature: F. E. Golling, M. Quernheim, M. Wagner, T. Nishiuchi, K. Müllen, *Angew. Chem. Int. Ed.* **2014**, *53*, 1525-1528.

3. X-Ray Crystallographic Analysis

Details of the crystal data and a summary of the intensity data collection parameters for **5** and **3** are listed in Tables **S1** and **S2**. In each case, suitable crystals were measured with STOE IPDS 2T diffractometer. Graphite-monochromated Mo Kα radiation was used. The structures were solved by direct methods with SIR-97 and refined by the full-matrix least-squares techniques against *F*2 (SHELXL-97). The intensities were corrected for Lorentz and polarization effects. The non-hydrogen atoms were refined anisotropically. Hydrogen atoms were refined using the riding model. The crystals structures were visualized using Mercury 3.3.

3.1 *X-ray crystal structure of* **5**

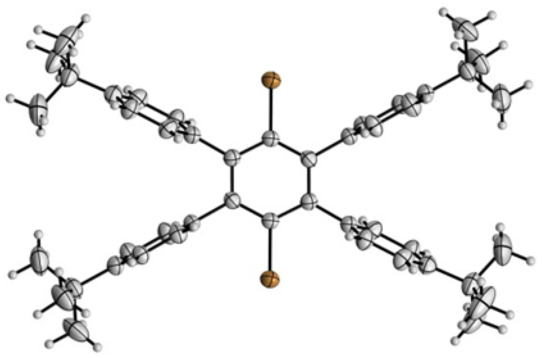

Figure S1: X-ray crystal structure of **5**.

Table S1: Crystallographic data and structure refinement details of **5**. Single crystals of **5** were obtained from dichloromethane/MeOH and of **9**. See CCDC 1033268.

Compound	**5**
Molecular Formula	$C_{46}H_{52}Br_2$
Formula Weight	764.69 gmol^{-1}
Crystal Dimensions	0.13 x 0.16 x 0.16 mm³
Crystal color	colorless
Crystal System	monoclinic
Space Group	P 2_1/c
a	12.9767(9)Å
b	10.3514(6)Å
c	15.6662(9)Å
α	
β	ß = 105.739(5)°
γ	
Cell Volume	V = 2025.5(2)Å^3
Z value	2
$D_{calc.}$	1.254 gcm^{-3}
F (000)	796
Temperature	- 60 °C
Total number of Reflections	13702
Unique number of Reflections (R_{int})	4974 (0.052)
Observed number of Reflections	3376
Residuals: R_1 (I>2.00σ(I))	0.0383
Residuals: R_1 (All Reflections)	0.0726
wR_2 (All Reflections)	0.1010
Goodness of Fit	1.021
Max Shift / Error	0.001 * e.s.d

3.2. *X-ray structure of* **3**

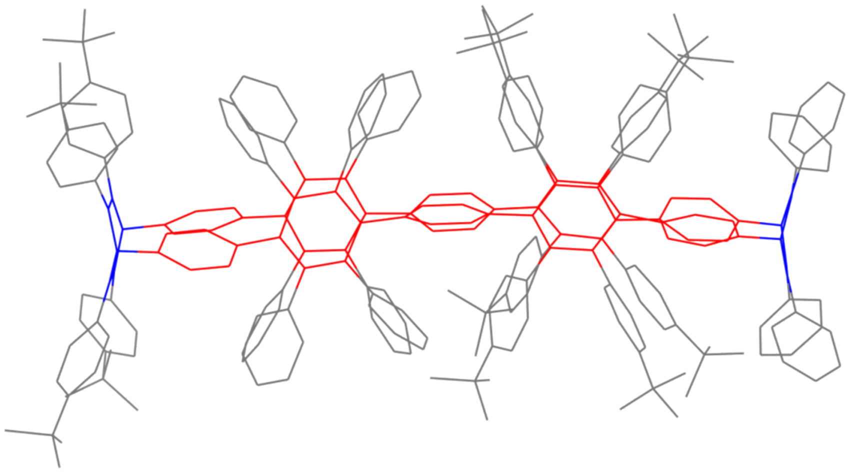

Figure S2: X-ray crystal structure of **3** in wireframe model (side view). Hydrogen atoms and solvent molecules are omitted for clarity. Pentaphenylenes are labeled in red, whereas the bridging cyclohexadiene units are labeled in blue.

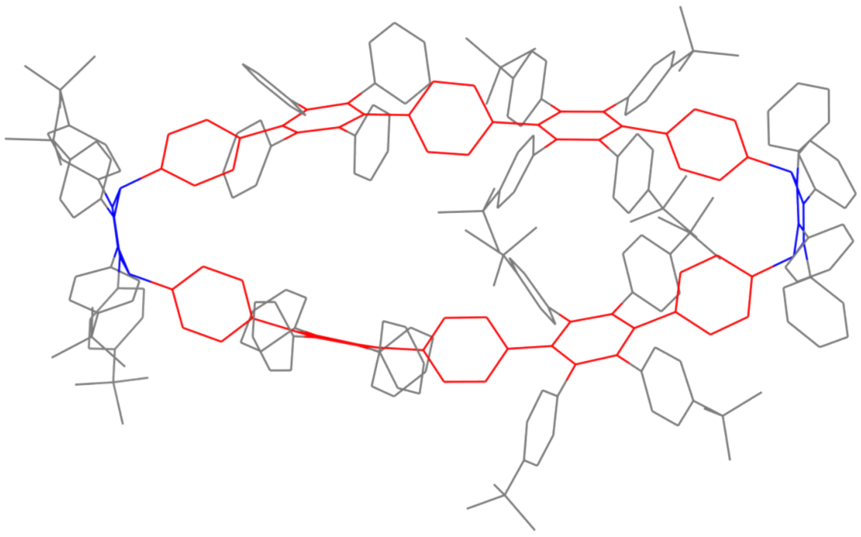

Figure S3: X-ray crystal structure of **2** in wireframe model (top view). Hydrogen atoms and solvent molecules are omitted for clarity. The pentaphenylene is labeled in red, whereas the bridging cyclohexadiene units are labeled in blue.

Comments: The structure of compound **3** is disordered. The solvent molecules (CH_2Cl_2, CH_3CN, H_2O) are severely disordered. The amount of solvent molecules cannot exactly be determined since the crystal contains large pores with varying amounts of solvent. This effect is amplified by loss of solvent (high vapor pressure of CH_2Cl_2 in combination with large pores) during crystal preparation. Thus, an accurate determination of solvent molecules within the voids is not possible. The voids of the crystal lattice are filled with a lot of scattered electron density; the SQUEEZE protocol was used to remove the void electron density for solvent molecules which could not be localized.

Due to the poor crystal quality and diffraction power, CheckCif gives 3 A and 15 B level alerts. Nearly all of these alerts (both A and B level) result from disordered solvent, large thermal movement of *t*-butyl and phenyl moieties, the high value of weighted R and the ratio of the observed/unique reflections.

Table S2: Crystallographic data and structure refinement details of **4** and **9**. Single crystals of **4** were obtained from dichloromethane/MeCN. See CCDC 1033267.

Compound	**3**
Molecular Formula	$C_{264}H_{244}$+mCH_2CL_2+nCH_3CN+k*H_2O
Formula Weight	3416 $gmol^{-1}$
Crystal Dimensions	0.12 x 0.38 x 0.6 mm³
Crystal color	Colorless
Crystal System	P 21/n
Space Group	monoklin
a	16.1937(15)Å
b	43.708(4)Å
c	33.799(3)Å
α	
β	92.792(1)°
γ	
Cell Volume	23894(7)$Å^3$
Z value	4
$D_{calc.}$	1.144 gcm^{-3}
F (000)	8704
Temperature	100 °C
Total number of Reflections	261715
Unique number of Reflections (R_{int})	56067 (0.1513)
Observed number of Reflections	21529
Residuals: R_1 (I>2.00σ(I))	0.1421
Residuals: R_1 (All Reflections)	0.2912
wR_2 (All Reflections)	0.3803
Goodness of Fit	1.675
Max Shift / Error	0.0001 * e.s.d.
Remark	Structure is disordered. Solvent molecules are disordered and amount of solvent could not be determined.

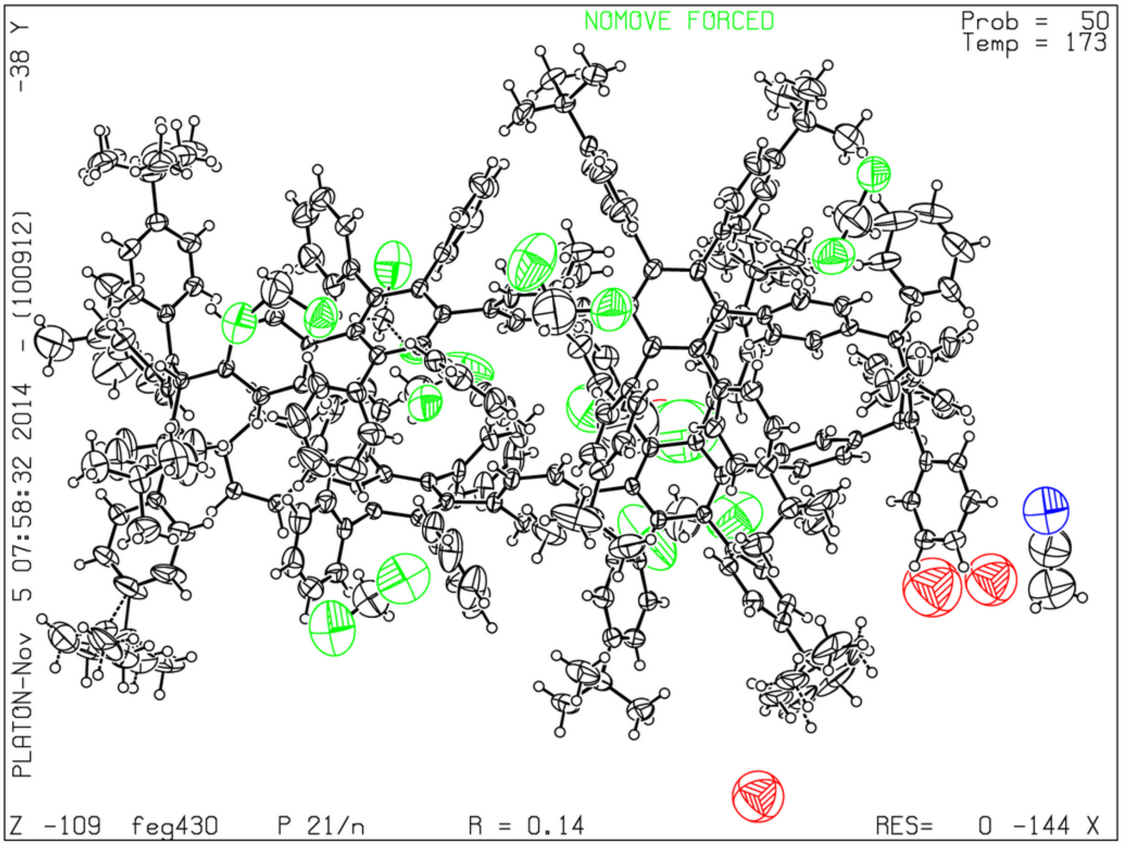

4. 2D-NMR Spectra

4.1 Compound **2**

4.1.1 *^{1}H-^{1}H-COSY and NOESY*

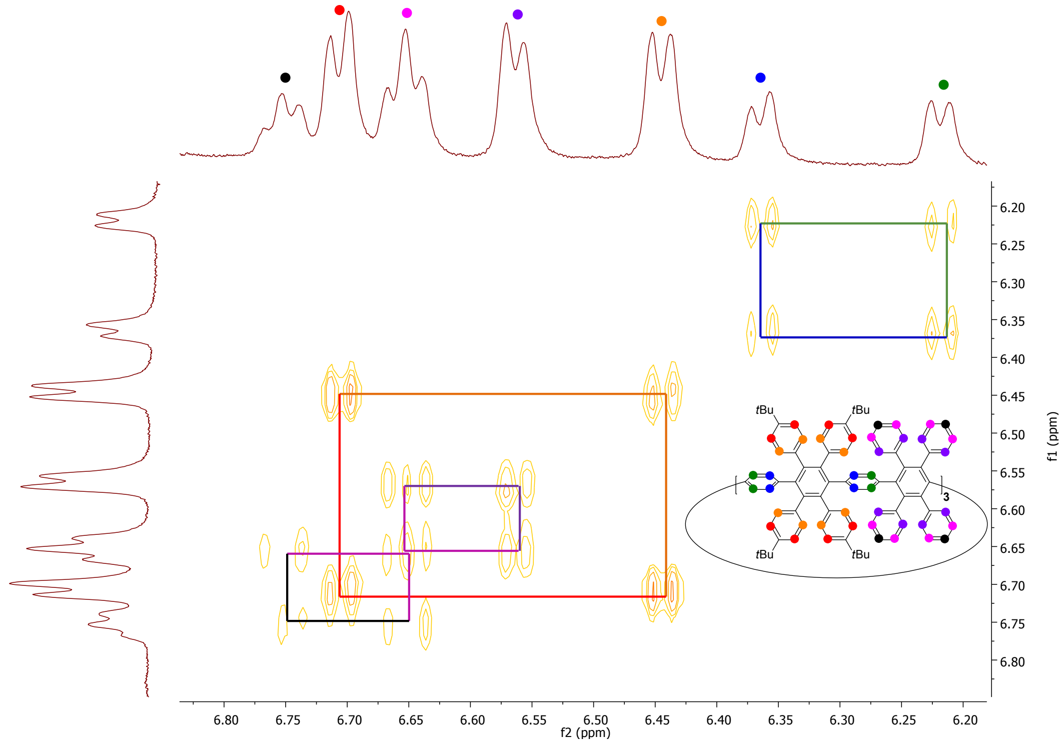

Figure S4: ^{1}H-^{1}H-COSY (500 MHz) of **2** recorded at 298 K in $C_2D_2Cl_4$.

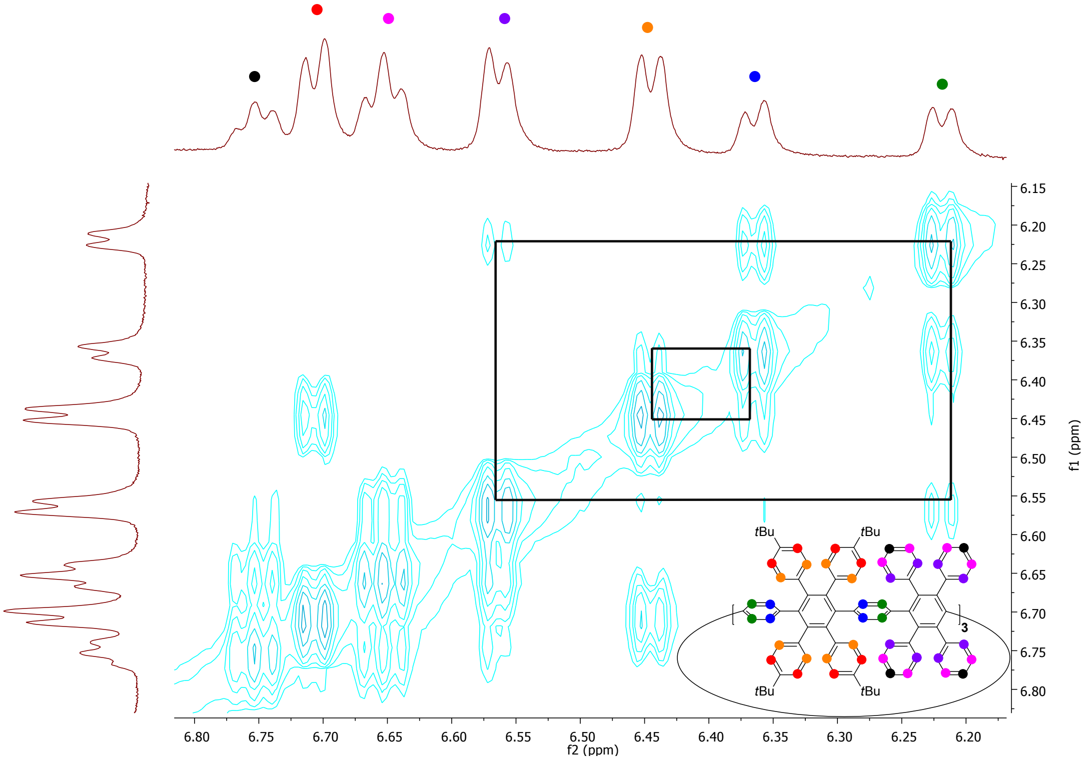

Figure S5: NOESY-spectrum of **2** recorded at 298 K in $C_2D_2Cl_4$. Intense through-space-coupling between the protons of the bridging phenylene with its neighboring 4-*t*BuPh and phenyl ring highlighted in black frames.

4.1.2 HSQC spectrum

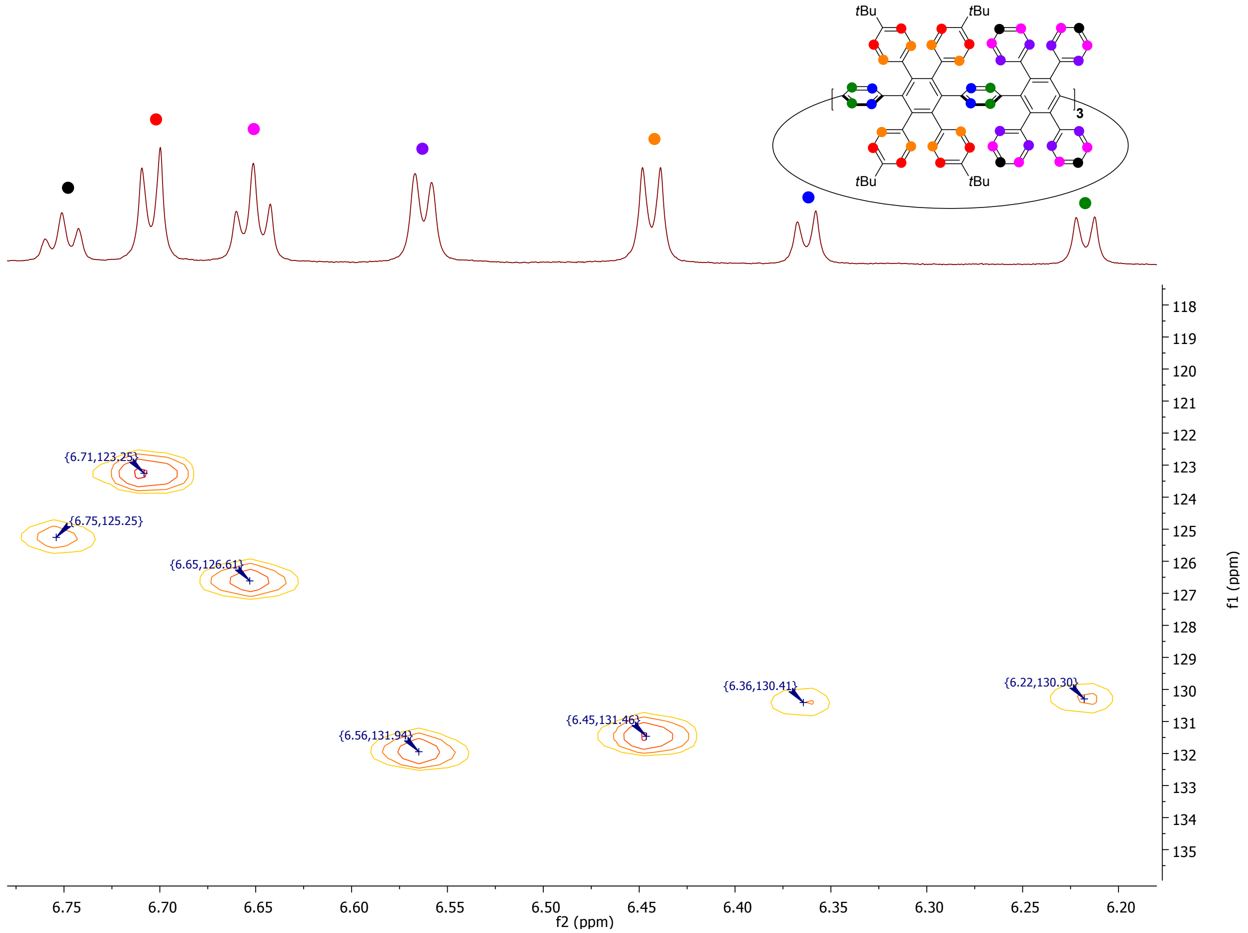

Figure S6: HSQC spectrum (850 MHz/214 MHz) of **2** recorded at 273 K in $C_2D_2Cl_4$.

4.1.3 HMBC spectrum

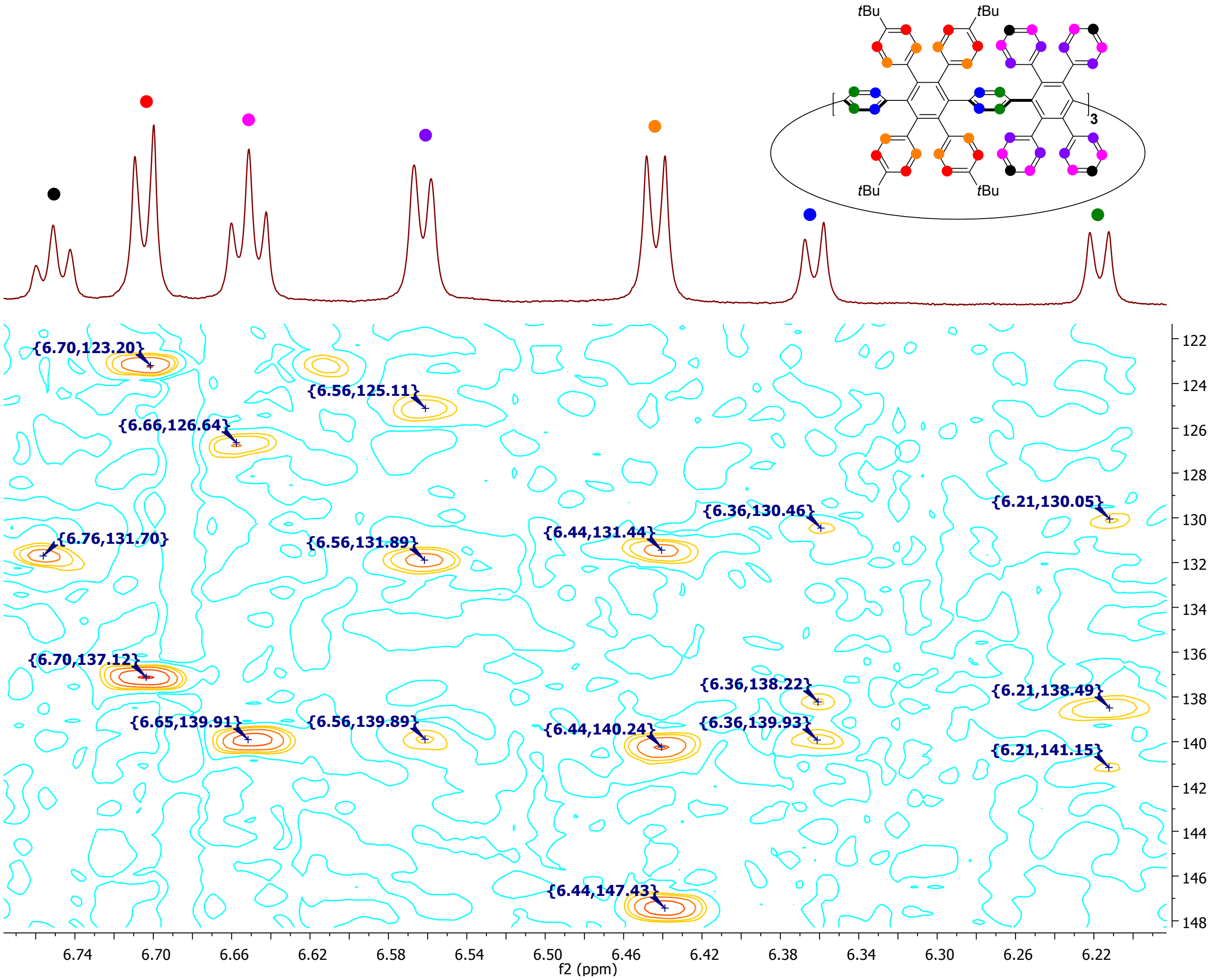

Figure S7: HMBC spectrum (850 MHz/214 MHz) of **2** recorded at 273 K in $C_2D_2Cl_4$. Neighboring *C*-H carbons are not detected due to the settings of the band pass filter.

4.2. *Compound **8***

4.2.1. *^{1}H-H^1-COSY*

The two colored rectangles belong to a split up A-B spin-system of compound **8** (see *Figure S8*): by integration and multiplicity (see *Figure S8*), these protons are attached to the phenylene ring, which bridges the cyclohexadiene and the *tetrakis*(4-*t*butylphenyl)benzene moieties. However, instead of the expected two signals, the A-B spin-system is split up into a spin-system with four different signals. This splitting is due to the interlocking of the phenylene ring which renders all four protons magnetically different, as two of those point inwards, whereas the other point outwards of the macrocycle and thus experience magnetically different surroundings.

In this spectrum, only the ^4J-couplings are highlighted by two colored rectangles, whereas the intense ^3J couplings are not visualized. These weak signals denote couplings between the two lightly labeled protons and the couplings between the darkly labeled protons.

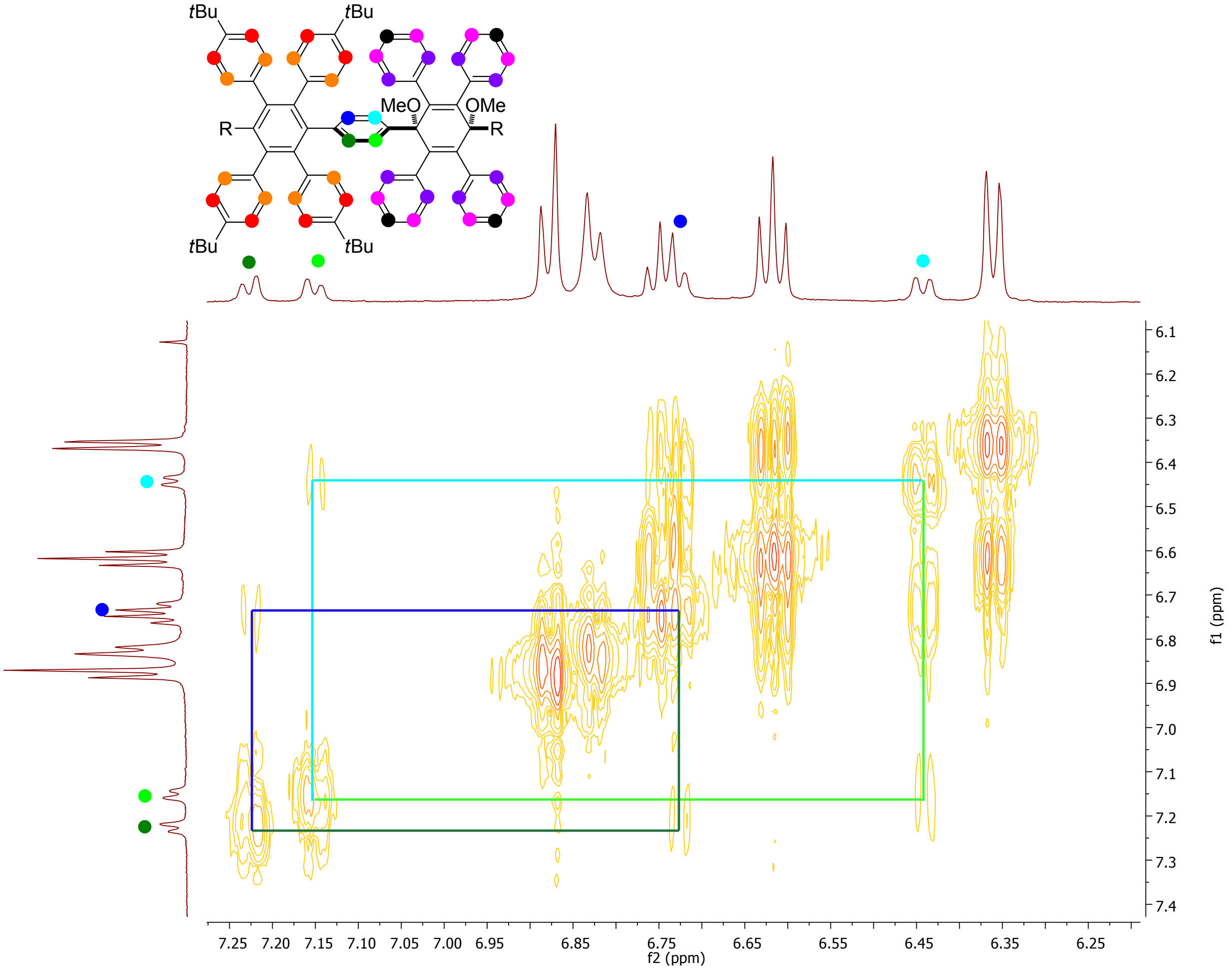

Figure S8: COSY (80°C, $C_2D_2Cl_4$, 500 MHz). The weak ^4J coupling is highlighted by the two rectangles.

4.2.2. NOESY

The coupling of the backwards pointing methoxy groups with the light blue labeled protons in the aromatic region of compound **8** shows a higher coupling intensity than the more remotely lying light green labeled proton (see *Figure S9*). This can be explained by the distant-dependent signal intensity inherent for NOE measurement, as the nuclear *Overhauser* effect (NOE) scales with $1/r^6$, with *r* being the distance between the interacting nuclei. In addition, a slight axial twisting of the phenylene ring is also necessary to give rise to through-space coupling, since the nuclei of the proton is otherwise hidden behind it. The question arises for why one does not observe the other two protons in this NOESY-spectrum. This can rationalized by two arguments: first, the kinked 1,4-dimethoxycyclohexa-2,5-diene moiety has the phenylene point away from the methoxy group (see *Scheme S1*). Therefore, the lightly colored protons are on the "visual axis" in between the methoxy group and the dark colored protons. Second, the distance between the protons is larger, rendering the signal intensity smaller.

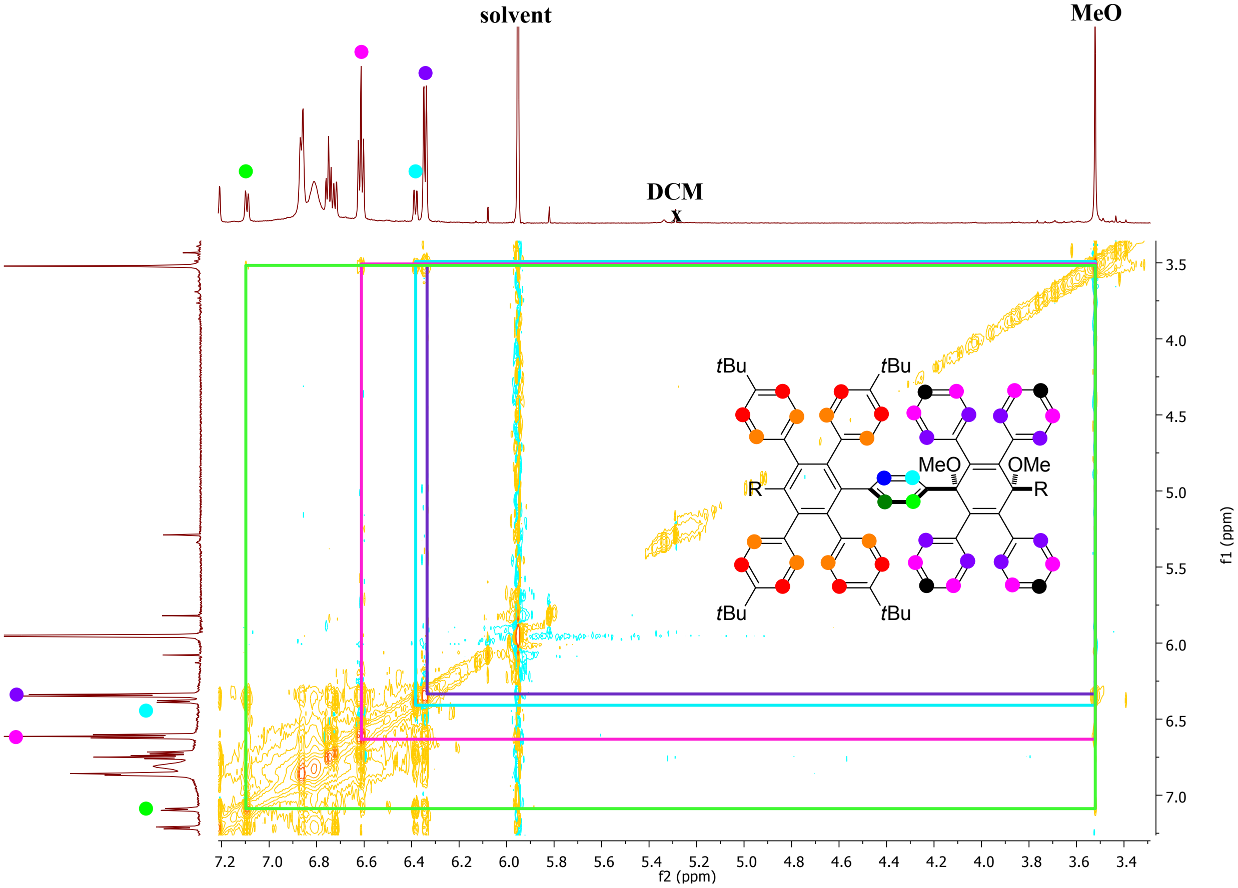

Figure S9: NOESY spectrum of compound **8** recorded at 60 °C in $C_2D_2Cl_4$ (700 MHz).

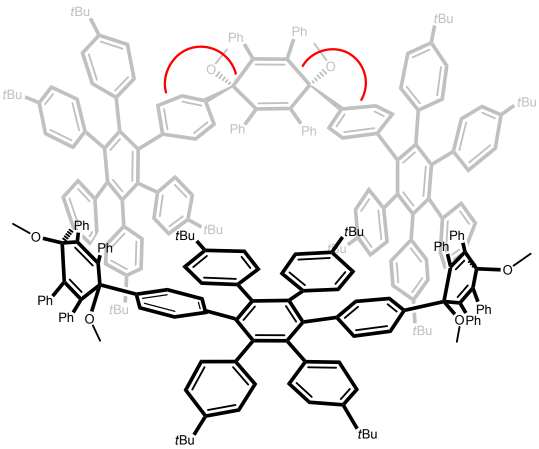

83

Scheme S1: 3D-representation of macrocycle **8** based on the 2D-NMR spectra discussed above. The expected angle of around. 90 ° between the phenylenes the cyclohexadiene unit is labeled with red semicircles.

5. Computational Results

The geometry optimization of all structures reported in *Scheme S2* has been performed using Density Functional Theory (DFT) with the B3LYP hybrid functional and the 6-31G(d) Pople's basis set. To reduce computation time, the methoxy groups of the synthesized structures have been removed and substituted by hydrogen atoms while we considered phenyl rings as R substituents.

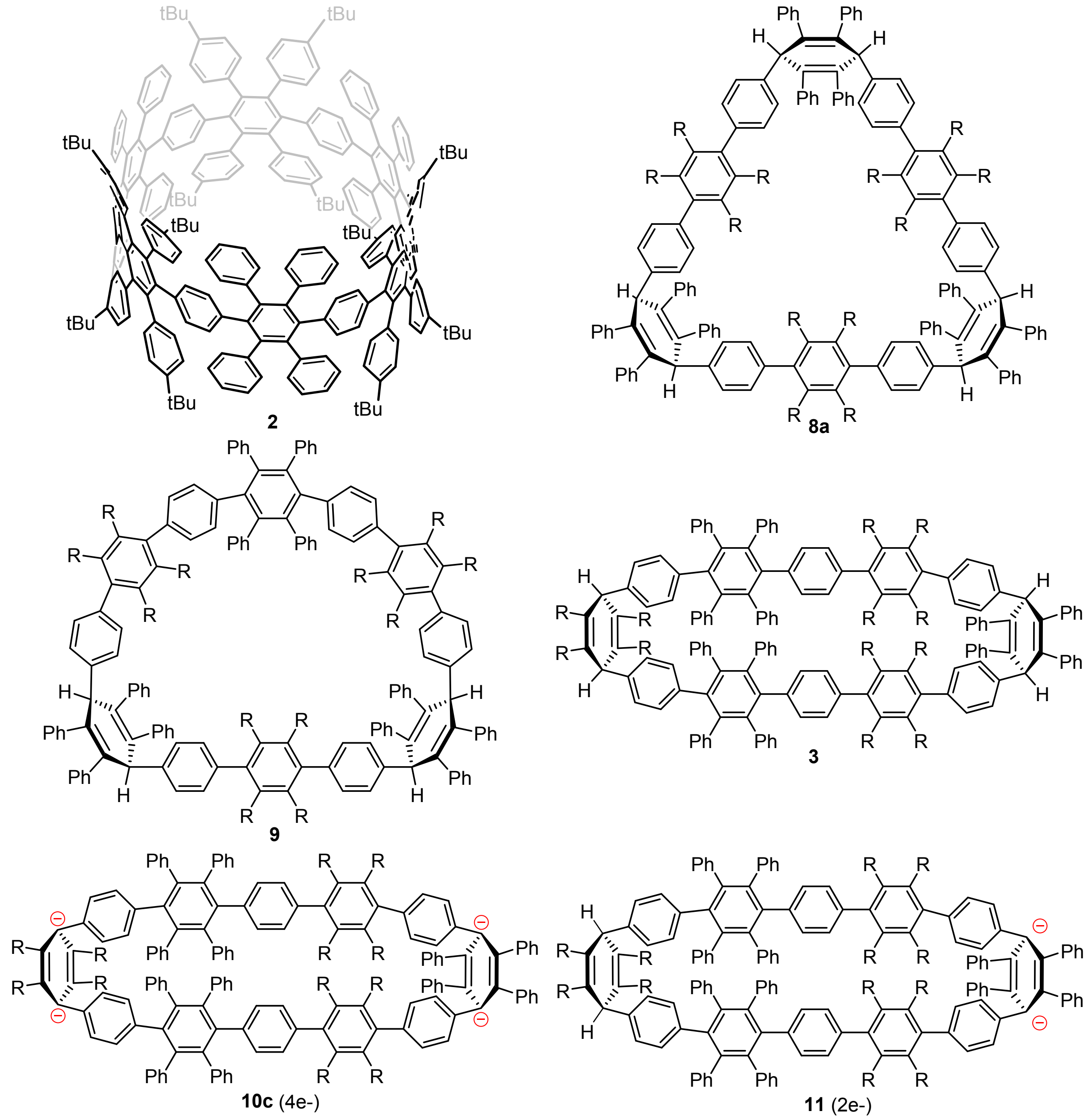

Scheme S2: structural overview of computed structures within this chapter.

5.1 Geometrical analysis

Here, the opening of the nanoring of the macrocycle is considered with the aim to understand whether the equilibrium structure of **10c** is rather close to compound **3** or **9**. By going from **3** to **10c**, there is a nanoring opening of about 4 Å (see *Figure S10*). In contrast, the nanoring opening of the neutral structure **9** is 13 Å. Hence, we can conclude that the equilibrium structure of the tetraanionic intermediate **10c** is shifted toward **3** in comparison to **9**, due to its oval shape. To support this result, a dianionic structure **11** was analyzed and it was found that the doubly negatively charged macrocycle has a similar shape to **3**, but with a smaller opening of the nanoring due to constrains of the two remaining hydrogen atoms bounded to the sp^3-carbons.

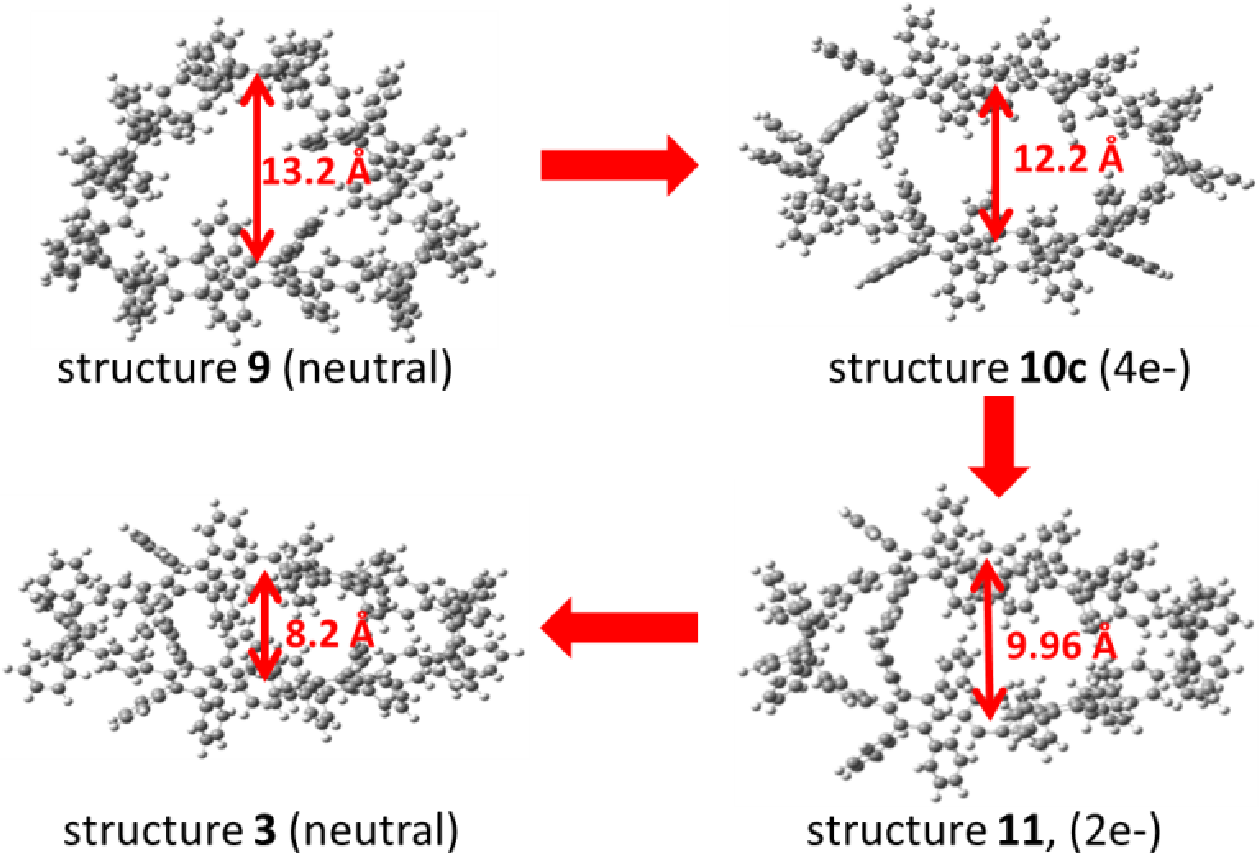

Figure S10: Nanoring opening for the different structures studied with different charge.

The bond length analysis of the structures **3** and **10c** shows two peculiar situations (see *Figure S11*): when the negatively charged structures **10c** is considered, the bond length is close to the aromatic bond length, in between 1.4-1.45Å (see *Figure S11*, top view left, black double arrows); on the other hand, for the neutral structure **3**, the bond length differs significantly. In fact, the bonds between the sp^3-carbon and the neighboring atoms is 1.53 Å, while the distance between the two sp^2-carbon atoms of the ring is 1.35 Å (see *Figure S11*, top view right, green double arrows). These results are in agreement with the bond angle analysis of the cyclohexadiene moieties. In fact, for the charged structures, the angles are close to the aromatic ring values, in

between 116-120 degrees, while for neutral structures the angles decrease to 110-113 degrees, close to a sp^3 hybridization of the carbon atoms (see *Figure S11*, top view, red dotted curves). The torsional angles analysis enforces this conclusion; in fact, the value of the out-of-plane bending of the phenyl substituents, bonded with the cyclohexadiene moiety, of ~38 degrees increases to ~65 degrees when passing to the neutral structure (see *Figure S11*, side view, black curves). Hence, we can conclude that the tetraaionic structure **10c** is a resonant form in between **3** and **9**, with a strong aromatic character of the anionic carbons that are sp^2 hybridized.

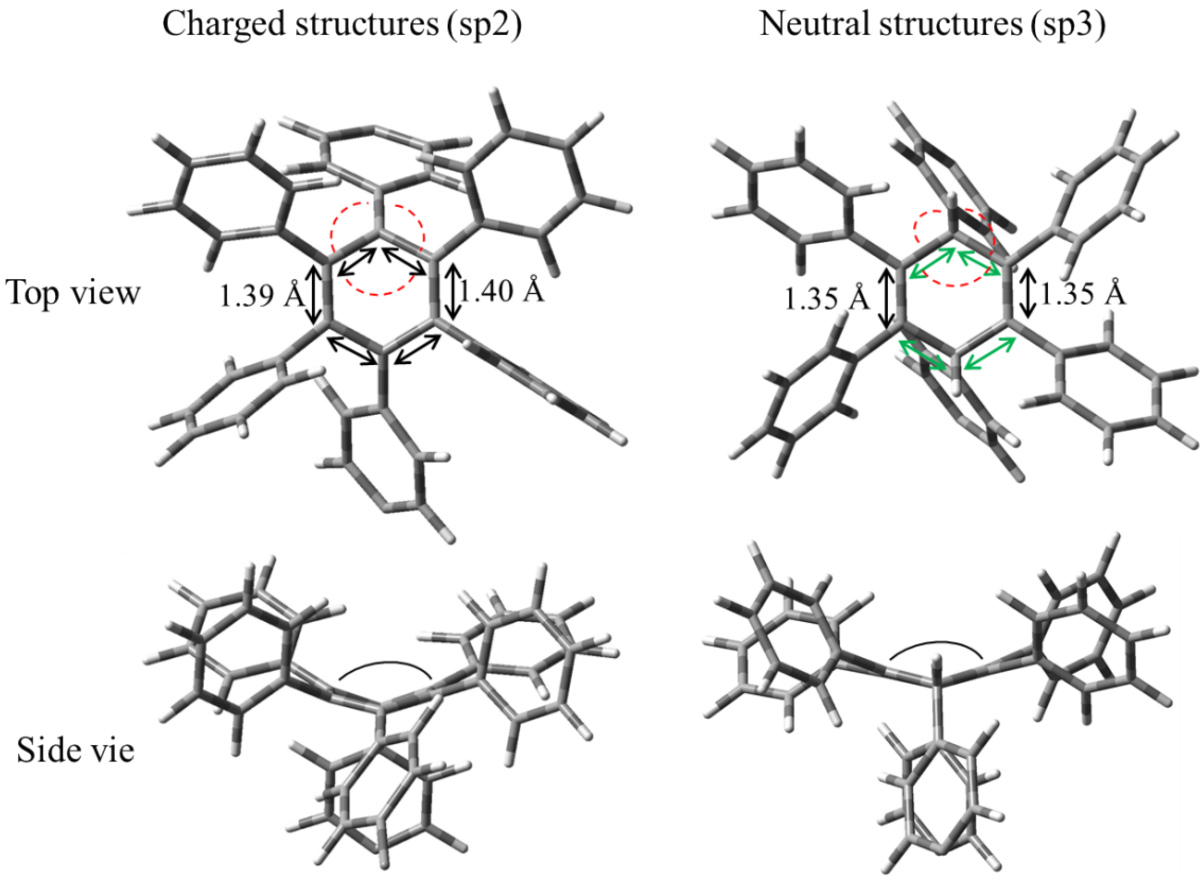

Figure S11: geometrical analysis of structures **3** (sp^2) and **10c**(sp^3, tetraanion). Black double arrows refer to the double bond length, while green double arrows refer to the single bonds length. Red dotted curves refer to the bond angles of the cyclohexadiene moieties, while black lines refer to the out-of-plane torsion angles.

5.2 Frontier Orbital Analysis

The frontier orbitals of the neutral structures **8a** and **3** are depicted in *Figure S12*.

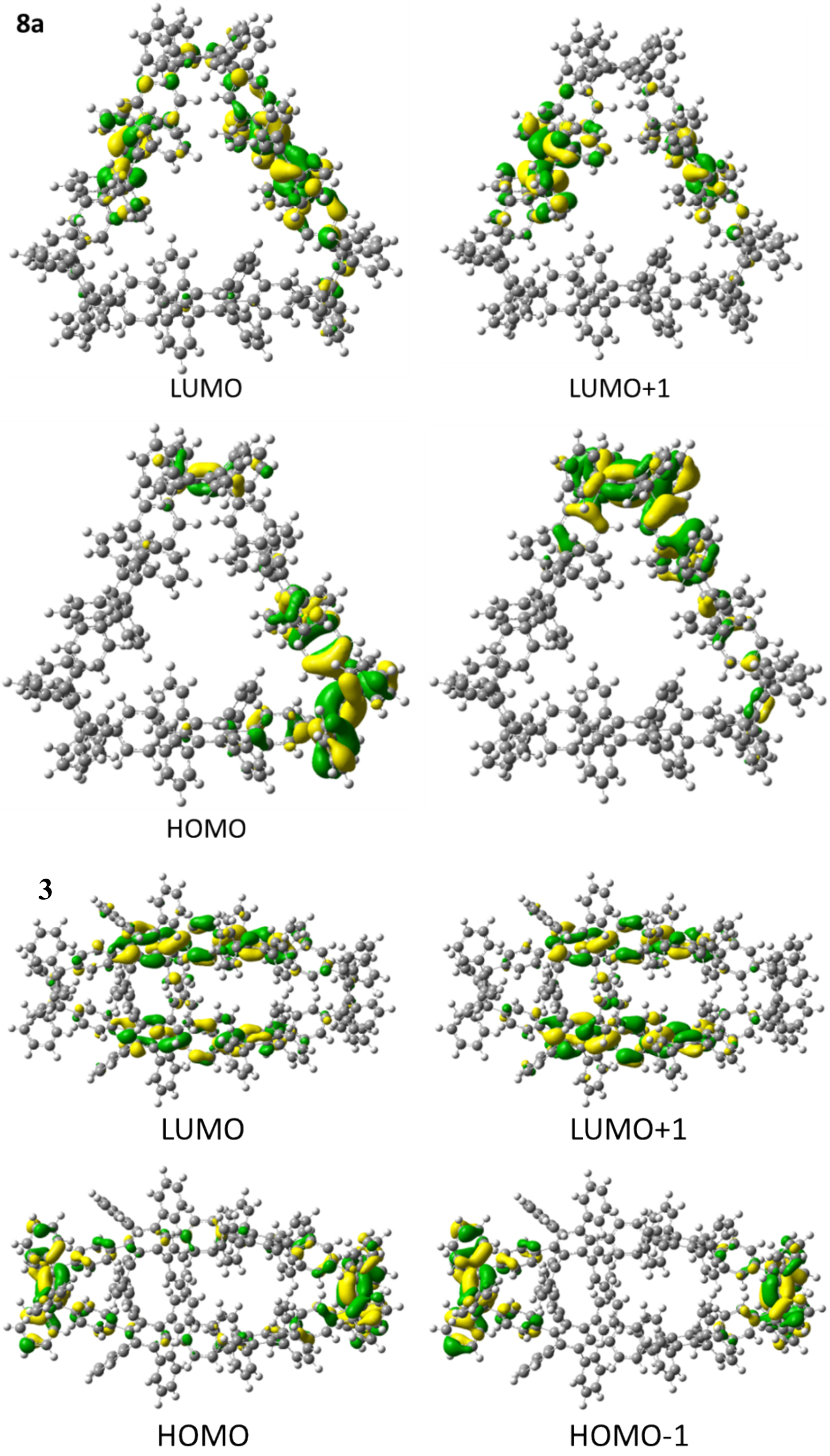

Figure S12: frontier orbital of **8a** (above) and **3** (below)

The HOMO of the neutral structures **8a** and **3** is localized over the ring bridging the sp^3-carbon atom, while the LUMO is localized over the sp^2 part of the macro ring. Due to the high symmetry of the structures, the last three occupied orbitals (namely HOMO-2, HOMO-1 and HOMO) are quasi-degenerate, as well as the first three virtual orbitals (LUMO, LUMO+1 and LUMO+2). Interestingly, for the neutral structure **9** (see *Figure S13*) both the HOMO and the LUMO are localized over the aromatized ring (previously sp^3 in **8a**; see upper image *Figure S12*).

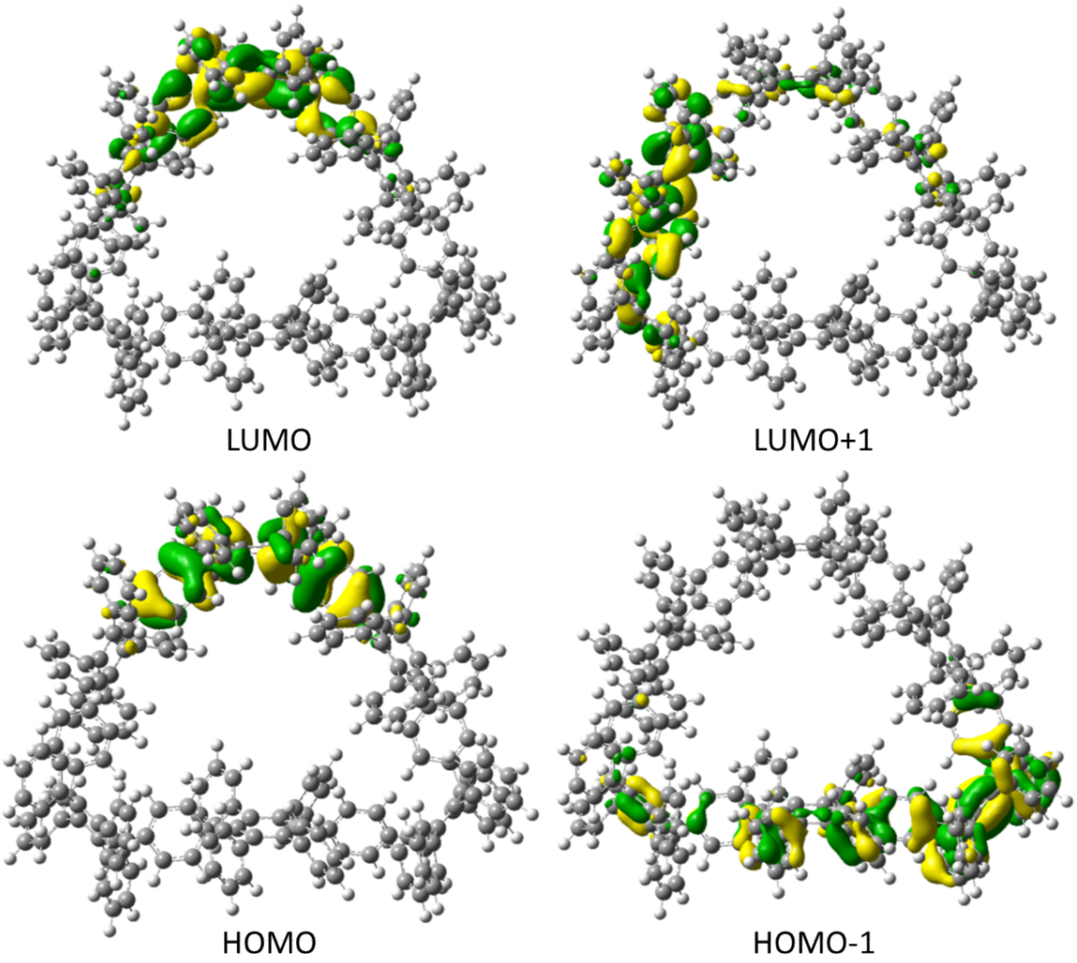

Figure S13: frontier orbitals of neutral structure **9.**

When the tetraanionic species **10c** is considered, the HOMO is localized over two aromatic rings, bridging the charge, while the LUMO is delocalized over the remaining part of the macro ring (see *Figure S13*). As for structures **3** and **8a**, the strong symmetry of the system leads to a quasi-degeneracy of the orbitals.

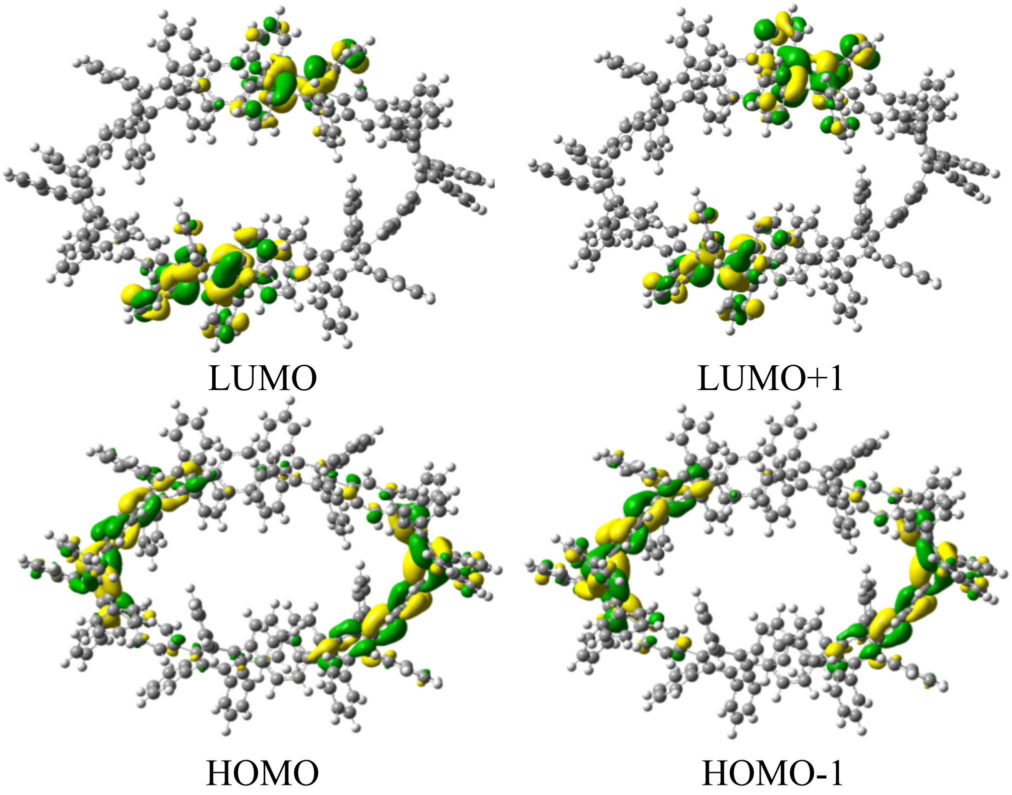

Figure S14: frontier orbitals of tetraanionic structure **3a**.

When an intermediate case is considered, in which two negative charges are removed (structure **11**), the HOMO is localized over the charged part of the molecule, while the LUMO is mainly localized over the cyclohexadiene moiety (see *Figure S14*).

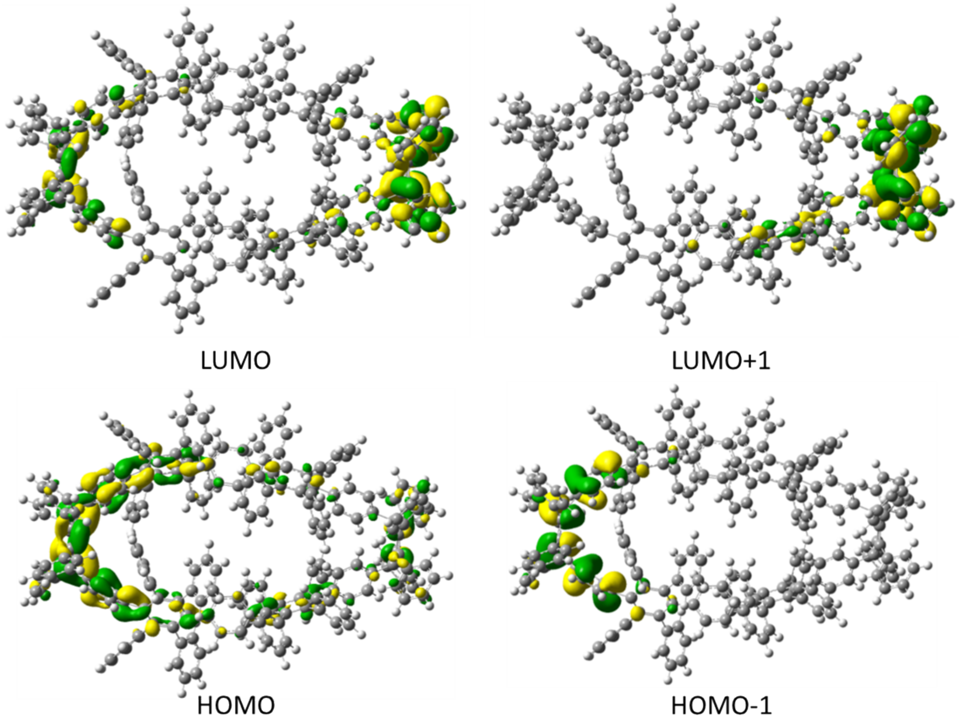

Figure S15: frontier orbital of dianionic macrocycle **3b**.

5.3 Electrostatic Potential (ESP) Analysis

To gain insight into the formation of structure **3** from structure **8** when a mild reductive aromatization was performed, we need to analyze the distribution of charges of our molecules. The electrostatic potential (ESP) analysis is therefore performed to assess the charge migration proposed in the mechanism. To analyze the charge distribution, we consider the charge on different subunits of the structure; in particular, the charge over tetraphenyl-1,4-phenylene moieties as one group and the phenylene ring linking them as a second group (see *Figure S16*, top).

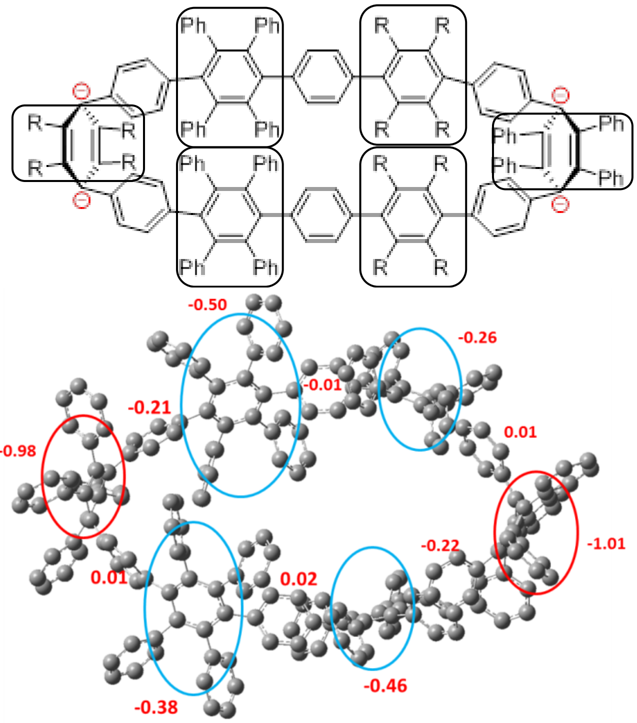

Figure S16: ESP of the tetraanionic structure **3a**. Schematic representation of the grouping of the charges (top) and actual charges found (bottom). Hydrogen atoms are not shown for clarity.

Interestingly, we did observe a strong localization of the charge (~2e) over the cyclohexadiene moieties (see *Figure S16*, red circles) while the remaining charge (~2e) is delocalized over the substituted aromatic phenyl rings (see *Figure S16*, blue circles).

5.4 Energy analysis

The total energy of compound **8a** and of the two isomers **9** and **3** are reported in the following table. Since **8a** has two hydrogen atoms more than **9** and **3**, we added the energy of H2 to the total energy of **9** and **3** to be comparable to **8a**. The energy difference (in kcal/mol) is also reported.

	**8a**	**9**	**3**
Total energy (Hartree)	-8321.3240	-8321.2853	-8321.3201
ΔE (kcal/mol)	0	24.28	2.47

5.5 Cartesian Coordinates

Structure 2: Atom number: 360

Total energy (Hartree): -8317.694435

C	-7.460167	5.195055	-0.731658
C	-6.611917	6.297382	-0.914827
C	-5.255268	6.228549	-0.579986
C	-4.778978	5.021084	-0.043945
C	-5.613192	3.928152	0.124887
C	-6.976128	3.984572	-0.216317
H	-8.505394	5.282771	-1.011664
H	-7.019833	7.213700	-1.331977
H	-3.729736	4.940497	0.227898
H	-5.201949	3.001654	0.512762
C	-4.188818	7.279440	-0.745248
C	-3.771882	8.028969	0.377273
C	-3.409555	7.305846	-1.921575
C	-2.466948	8.572366	0.420654
C	-2.111607	7.859782	-1.884959
C	-1.589541	8.355166	-0.665283
C	-0.098570	8.331172	-0.471754
C	0.756725	9.380562	-0.110332
C	0.463599	7.046967	-0.582311
C	2.117159	9.151486	0.148367
H	0.362918	10.387464	-0.009484
C	1.805399	6.821263	-0.325443
H	-0.175245	6.211248	-0.850886
C	2.662001	7.865932	0.058315
H	2.750414	9.987327	0.431952
H	2.205597	5.814471	-0.414108
C	4.073267	7.422390	0.331673
C	4.356634	6.772732	1.553893
C	5.039642	7.413715	-0.697811
C	5.475128	5.919440	1.651329
C	6.151278	6.542184	-0.606159
C	6.290029	5.699521	0.516972
C	6.976860	4.364061	0.392185
C	8.341149	4.055795	0.440870
C	6.080125	3.304271	0.175271
C	8.791405	2.736266	0.284265
H	9.068900	4.844596	0.604937
C	6.524299	2.003171	0.012923

H	5.015327	3.514477	0.128503
C	7.891729	1.686211	0.063963
H	9.856420	2.530936	0.342525
H	5.802025	1.208275	-0.148001
C	8.187030	0.218776	-0.079546
C	7.816551	-0.418104	-1.287365
C	8.562932	-0.566602	1.031823
C	7.577780	-1.809970	-1.306384
C	8.332220	-1.961829	1.008249
C	7.733182	-2.561644	-0.122285
C	-7.710232	2.681402	-0.062207
C	-7.664258	2.033331	1.196970
C	-8.213831	1.976618	-1.179147
C	-7.839710	0.633766	1.276999
C	-8.399410	0.577277	-1.094458
C	-8.096194	-0.107359	0.104290
C	-7.792062	-1.581725	0.061163
C	-8.624113	-2.638519	0.449531
C	-6.503631	-1.903063	-0.393137
C	-8.182945	-3.968297	0.395386
H	-9.627972	-2.430029	0.808901
C	-6.061478	-3.216854	-0.431664
H	-5.835390	-1.103083	-0.700942
C	-6.887947	-4.284174	-0.037988
H	-8.853860	-4.760479	0.712875
H	-5.043686	-3.427089	-0.745594
C	7.016991	-3.877943	0.025377
C	5.743496	-3.788295	0.609541
C	7.448024	-5.140971	-0.395310
C	4.923959	-4.899939	0.724224
H	5.383847	-2.821555	0.951963
C	6.626861	-6.269946	-0.261927
H	8.429018	-5.253128	-0.848715
C	5.338595	-6.169694	0.282838
H	3.928852	-4.783016	1.140948
H	6.992789	-7.230019	-0.611828
C	4.275308	-7.231271	0.345452
C	3.756601	-7.820113	-0.831704
C	3.603708	-7.452445	1.574424
C	2.438478	-8.329427	-0.829404
C	2.296454	-7.988982	1.579220
C	1.664305	-8.290367	0.352766

C	0.163406	-8.301452	0.250129
C	-0.390076	-7.139692	-0.312738
C	-0.725272	-9.278639	0.712410
C	-1.761366	-6.953535	-0.379414
H	0.273187	-6.359777	-0.676445
C	-2.113263	-9.095759	0.632036
H	-0.339349	-10.191238	1.157808
C	-2.664565	-7.921926	0.098351
H	-2.149585	-6.024720	-0.785476
H	-2.763309	-9.879012	1.005134
C	-6.215605	-5.627226	-0.044029
C	-5.939124	-6.320909	1.153608
C	-5.655715	-6.110362	-1.254669
C	-4.870669	-7.250500	1.197538
C	-4.614913	-7.056808	-1.216570
C	-4.107329	-7.498279	0.035543
C	-2.026705	9.371387	1.611912
C	-1.093336	8.894738	2.542320
C	-2.561147	10.655909	1.797088
C	-0.709063	9.679645	3.630177
H	-0.655136	7.909910	2.412299
C	-2.177498	11.441537	2.883691
H	-3.286194	11.036584	1.082988
C	-1.249330	10.954376	3.805042
H	0.023259	9.292547	4.332412
H	-2.604024	12.433591	3.008452
H	-0.947165	11.565003	4.652055
C	-4.705799	8.179529	1.538485
C	-5.893658	8.909758	1.384111
C	-4.434960	7.602051	2.786838
C	-6.781342	9.067857	2.448423
H	-6.114380	9.364762	0.422037
C	-5.324270	7.752559	3.849779
H	-3.522858	7.029298	2.923393
C	-6.499303	8.488038	3.685771
H	-7.692922	9.643542	2.309438
H	-5.099227	7.291162	4.807500
H	-7.189815	8.608166	4.516494
C	-1.271247	7.940472	-3.124857
C	-0.832339	6.807795	-3.826813
C	-0.919808	9.205750	-3.620624
C	-0.076071	6.935917	-4.991134

H	-1.086980	5.818610	-3.460356
C	-0.170398	9.337514	-4.789609
H	-1.249353	10.092762	-3.086548
C	0.252182	8.201557	-5.481928
H	0.252369	6.044539	-5.519395
H	0.080965	10.328198	-5.159389
H	0.828574	8.300495	-6.398281
C	-3.935130	6.731172	-3.205603
C	-3.910750	5.354308	-3.468948
C	-4.419527	7.593657	-4.199295
C	-4.331545	4.857014	-4.702995
H	-3.547914	4.671300	-2.706667
C	-4.849752	7.097982	-5.430395
H	-4.442306	8.663020	-4.007984
C	-4.798131	5.727784	-5.690264
H	-4.287165	3.788000	-4.896200
H	-5.215193	7.784384	-6.189758
H	-5.115169	5.341203	-6.655520
C	3.486644	6.992584	2.759963
C	2.314520	6.258366	2.989035
C	3.881850	7.933815	3.721258
C	1.569127	6.446336	4.153675
H	1.989101	5.528195	2.254652
C	3.137603	8.126302	4.885653
H	4.788432	8.509901	3.557252
C	1.981334	7.377355	5.109660
H	0.670900	5.856681	4.319015
H	3.466754	8.855883	5.621042
H	1.408099	7.513825	6.023101
C	5.793321	5.218360	2.937825
C	4.975211	4.210884	3.469161
C	6.939134	5.592646	3.655288
C	5.283014	3.608222	4.688324
H	4.090505	3.898320	2.923517
C	7.245706	4.997913	4.879586
H	7.584935	6.368532	3.252877
C	6.414729	4.006235	5.403514
H	4.633478	2.831727	5.084143
H	8.131751	5.312847	5.424792
H	6.644740	3.548336	6.362222
C	7.134372	6.435937	-1.730989
C	8.455482	6.870672	-1.547845

C	6.771822	5.905062	-2.976497
C	9.387299	6.786785	-2.582907
H	8.747097	7.289839	-0.588097
C	7.703180	5.814271	-4.010011
H	5.404419	1.446143	-4.858349
C	9.012919	6.257491	-3.818520
H	10.404438	7.135577	-2.423209
H	7.404042	5.394098	-4.966342
H	9.736683	6.190812	-4.626698
C	4.893232	8.324244	-1.881798
C	3.898316	8.153011	-2.854541
C	5.782077	9.402181	-2.018853
C	3.798384	9.031715	-3.934038
H	3.193104	7.332718	-2.768254
C	5.681705	10.282297	-3.095514
H	6.558115	9.547963	-1.272566
C	4.688378	10.098907	-4.058753
H	3.014440	8.880749	-4.670555
H	6.380022	11.111060	-3.180503
H	4.608246	10.783609	-4.899249
C	9.199780	0.071411	2.230819
C	8.491082	0.909544	3.102696
C	10.558303	-0.168017	2.488810
C	9.122137	1.485439	4.205887
H	7.442351	1.120039	2.915669
C	11.190953	0.410033	3.588985
H	11.118566	-0.817068	1.821543
C	10.473289	1.238663	4.452864
H	8.553220	2.136130	4.863137
H	12.244204	0.211285	3.770431
H	10.964178	1.689744	5.311504
C	8.657890	-2.812659	2.197524
C	9.637308	-3.811784	2.094468
C	8.001922	-2.646083	3.425580
C	9.959164	-4.615186	3.188326
H	10.154998	-3.951497	1.149127
C	8.316998	-3.452670	4.518160
H	7.239144	-1.879653	3.523572
C	9.298624	-4.438491	4.404635
H	10.725308	-5.379934	3.088655
H	7.792514	-3.311179	5.459408
H	9.546444	-5.064563	5.257833

C	7.675171	0.406408	-2.532855
C	8.808387	1.040939	-3.064257
C	6.450845	0.569079	-3.197439
C	8.725289	1.802610	-4.229609
H	9.763695	0.928424	-2.558683
C	6.363997	1.333396	-4.360111
H	5.559697	0.092516	-2.801810
C	7.501785	1.949490	-4.883846
H	9.616850	2.283431	-4.623389
H	5.755067	5.557748	-3.132552
H	7.434754	2.539384	-5.794357
C	7.124814	-2.455292	-2.584103
C	5.776858	-2.762779	-2.818392
C	8.052933	-2.696063	-3.606794
C	5.363847	-3.263287	-4.053526
H	5.046816	-2.592690	-2.032636
C	7.644174	-3.206170	-4.839313
H	9.100290	-2.460471	-3.439137
C	6.295542	-3.480297	-5.071443
H	4.311257	-3.475262	-4.224296
H	8.378563	-3.376496	-5.622274
H	5.971539	-3.856638	-6.038455
C	4.301324	-7.107544	2.855655
C	3.772681	-6.197988	3.784181
C	5.534365	-7.711652	3.147762
C	4.453044	-5.903700	4.964743
H	2.821650	-5.716674	3.579741
C	6.214471	-7.422393	4.330250
H	5.959093	-8.416659	2.438490
C	5.675732	-6.516378	5.244245
H	4.025646	-5.193146	5.667693
H	7.167352	-7.903481	4.534775
H	6.204282	-6.288535	6.166143
C	1.586401	-8.235253	2.877434
C	2.005800	-9.294011	3.696520
C	0.515656	-7.439763	3.307656
C	1.377557	-9.547473	4.915716
H	2.836875	-9.916040	3.375674
C	-0.108429	-7.688059	4.529785
H	0.168038	-6.624734	2.679703
C	0.318885	-8.742368	5.337919
H	1.718337	-10.372701	5.535840

H	-0.937867	-7.062204	4.844238
H	-0.171591	-8.936723	6.288179
C	1.832788	-8.891840	-2.078674
C	1.664833	-8.118536	-3.236638
C	1.415878	-10.231357	-2.106879
C	1.103590	-8.668652	-4.387988
H	1.976250	-7.078524	-3.233307
C	0.859459	-10.785395	-3.259333
H	1.538642	-10.842819	-1.217068
C	0.701655	-10.005124	-4.405073
H	0.980333	-8.050955	-5.273843
H	0.548249	-11.826900	-3.260529
H	0.266842	-10.434386	-5.303835
C	4.603336	-7.954519	-2.063789
C	4.938745	-6.876775	-2.893289
C	5.095209	-9.228351	-2.390252
C	5.739520	-7.069059	-4.019895
H	4.585207	-5.879181	-2.651548
C	5.895253	-9.422185	-3.515870
H	4.841585	-10.072401	-1.754760
C	6.219629	-8.340616	-4.335919
H	5.996220	-6.216737	-4.641364
H	6.264055	-10.417599	-3.750055
H	6.844590	-8.486637	-5.213248
C	-4.536361	-7.917637	2.496391
C	-4.042716	-7.194888	3.591265
C	-4.775491	-9.290717	2.658028
C	-3.800422	-7.825274	4.811521
H	-3.859936	-6.129606	3.486331
C	-4.529468	-9.924744	3.876446
H	-5.175268	-9.860076	1.822541
C	-4.041520	-9.192360	4.958915
H	-3.430417	-7.244726	5.652832
H	-4.726164	-10.988691	3.980708
H	-3.854742	-9.681501	5.911168
C	-6.794702	-6.111132	2.369030
C	-7.709768	-7.117576	2.716863
C	-6.737529	-4.958199	3.162058
C	-8.538592	-6.981246	3.829838
H	-7.766547	-8.015108	2.107092
C	-7.565939	-4.820632	4.276685
H	-6.052083	-4.157890	2.899820

C	-8.467148	-5.830313	4.615710
H	-9.239218	-7.773653	4.080714
H	-7.514222	-3.913306	4.869890
H	-9.112133	-5.718559	5.483472
C	-4.065352	-7.663373	-2.473242
C	-3.364275	-6.931977	-3.442193
C	-4.271146	-9.033541	-2.698892
C	-2.893709	-7.545939	-4.602191
H	-3.175003	-5.875687	-3.282600
C	-3.806402	-9.649616	-3.860502
H	-4.810141	-9.615440	-1.956175
C	-3.116715	-8.906317	-4.818512
H	-2.349000	-6.959124	-5.337239
H	-3.983161	-10.710957	-4.015051
H	-2.752250	-9.383437	-5.724340
C	-6.273928	-5.693247	-2.561381
C	-7.439069	-6.353083	-2.979622
C	-5.751063	-4.686843	-3.384342
C	-8.056648	-6.026799	-4.187272
H	-7.858719	-7.133810	-2.350954
C	-6.368337	-4.355000	-4.590291
H	-4.857639	-4.151547	-3.077485
C	-7.521758	-5.025896	-4.998116
H	-8.956441	-6.555012	-4.492129
H	-5.946877	-3.568323	-5.210587
H	-8.000806	-4.768245	-5.938976
C	-7.719381	-0.045254	2.610556
C	-6.548337	-0.715198	2.992904
C	-8.770306	0.045019	3.534196
C	-6.418544	-1.248044	4.275820
H	-5.728338	-0.802835	2.286167
C	-8.647243	-0.497004	4.813704
H	-9.681760	0.564688	3.251825
C	-7.465459	-1.134710	5.193400
H	-5.494372	-1.744243	4.561850
H	-9.470358	-0.408369	5.517917
H	-7.359169	-1.537680	6.197431
C	-7.451807	2.858443	2.431980
C	-8.398213	3.843498	2.755529
C	-6.355097	2.686643	3.289425
C	-8.261282	4.623006	3.903603
H	-9.251925	3.991721	2.099919

C	-6.213923	3.467137	4.435851
H	-5.606912	1.935636	3.058112
C	-7.168618	4.435652	4.750436
H	-9.008408	5.377868	4.134155
H	-5.355025	3.316622	5.084942
H	-7.060705	5.040029	5.647213
C	-8.862828	-0.203665	-2.285991
C	-10.090361	-0.881231	-2.244828
C	-8.096191	-0.284250	-3.457410
C	-10.543222	-1.611376	-3.343580
H	-10.696539	-0.825741	-1.344617
C	-8.544008	-1.018522	-4.554601
H	-7.142140	0.231798	-3.506698
C	-9.770465	-1.682736	-4.502954
H	-11.499349	-2.125833	-3.291997
H	-7.933410	-1.070660	-5.452274
H	-10.119900	-2.253945	-5.358860
C	-8.577478	2.701371	-2.441811
C	-9.935985	2.855434	-2.759673
C	-7.622531	3.244011	-3.311667
C	-10.329812	3.527875	-3.916236
H	-10.686724	2.439122	-2.093681
C	-8.014987	3.916539	-4.469590
H	-6.566472	3.152241	-3.076367
C	-9.368632	4.060515	-4.776441
H	-11.387460	3.634960	-4.143336
H	-7.257646	4.340293	-5.122031
H	-9.672140	4.587069	-5.677608

Structure 8a: Atom numer: 366

Total energy (Hartree): -8321.324018

C	-5.071178	-0.130640	-4.351562
C	-5.725870	-1.360094	-4.255378
C	-5.698442	-2.078213	-3.059528
C	-5.021265	-1.581119	-1.936975
C	-4.371416	-0.343010	-2.044642
C	-4.393239	0.375599	-3.240358
C	-4.982869	-2.365412	-0.657978
C	-5.696387	-1.915164	0.474024
C	-5.650011	-2.651168	1.677770
C	-4.912570	-3.855165	1.740259
C	-4.209902	-4.311374	0.604430

C	-4.231606	-3.557958	-0.589459
C	-6.494022	-0.647079	0.387780
C	-7.733568	-0.620738	-0.264535
C	-8.463415	0.562064	-0.370998
C	-7.967322	1.767960	0.141113
C	-6.739950	1.734882	0.814266
C	-6.019330	0.547895	0.942292
C	-8.754253	3.075815	-0.058200
C	-8.612342	4.022725	1.124139
C	-7.973627	5.204777	1.027275
C	-7.381427	5.708774	-0.290724
C	-7.859864	4.933286	-1.509973
C	-8.465009	3.735183	-1.410548
C	-9.250108	3.544930	2.391660
C	-10.625476	3.261260	2.426147
C	-11.236676	2.803406	3.593024
C	-10.480272	2.606764	4.749454
C	-9.111267	2.876510	4.727930
C	-8.503616	3.341667	3.562072
C	-9.033229	3.023239	-2.601886
C	-8.334964	2.007378	-3.271473
C	-8.909707	1.343996	-4.356782
C	-10.193421	1.680041	-4.789048
C	-10.900481	2.686491	-4.128789
C	-10.325107	3.348686	-3.043874
C	-7.926093	6.189437	2.156261
C	-6.747664	6.443342	2.875295
C	-6.728870	7.396728	3.894858
C	-7.880957	8.118731	4.209160
C	-9.057732	7.878103	3.498137
C	-9.077927	6.923295	2.481506
C	-7.642313	5.621023	-2.821543
C	-6.830784	5.064757	-3.821070
C	-6.648471	5.716473	-5.040995
C	-7.266696	6.944116	-5.283095
C	-8.068199	7.515475	-4.293601
C	-8.249697	6.861704	-3.075239
C	-5.849158	5.828088	-0.266274
C	-5.231287	7.074228	-0.110587
C	-3.841577	7.193020	-0.076796
C	-3.021250	6.064057	-0.191362
C	-3.641052	4.816860	-0.356850

C	-5.028179	4.701827	-0.403670
C	-1.524451	6.160823	-0.157321
C	-0.814740	5.643084	0.948587
C	0.596713	5.683888	0.964592
C	1.297761	6.219313	-0.137478
C	0.589593	6.746920	-1.238707
C	-0.823241	6.734664	-1.241082
C	-1.560667	5.049278	2.107126
C	-1.497613	3.673798	2.372869
C	-2.192114	3.119210	3.448088
C	-2.957347	3.933460	4.284164
C	-3.023207	5.305607	4.034480
C	-2.334970	5.856673	2.952914
C	-1.574698	7.348970	-2.384591
C	-2.352087	6.569551	-3.252587
C	-3.055618	7.156870	-4.303651
C	-2.995015	8.536442	-4.505081
C	-2.223567	9.323642	-3.649203
C	-1.519372	8.734087	-2.599669
C	1.351222	5.168183	2.155402
C	2.118761	3.996838	2.078722
C	2.819170	3.524024	3.189217
C	2.764605	4.219494	4.399496
C	2.005637	5.387920	4.488862
C	1.304977	5.855578	3.376882
C	1.340932	7.288050	-2.418973
C	2.116917	8.451411	-2.316676
C	2.821079	8.940043	-3.416854
C	2.764826	8.270805	-4.640142
C	1.996225	7.111735	-4.755278
C	1.289975	6.626843	-3.654834
C	2.797773	6.205313	-0.142520
C	3.540727	7.111601	0.624522
C	4.934160	7.077703	0.623621
C	5.636213	6.115037	-0.114437
C	4.891736	5.221925	-0.894301
C	3.498691	5.271300	-0.914694
C	7.173550	6.060026	-0.049885
C	7.791904	5.562773	-1.349175
C	8.429101	4.377346	-1.432190
C	8.619048	3.469238	-0.214161
C	8.385207	4.180524	1.110361

C	7.696749	5.333030	1.193660
C	7.641917	6.490273	-2.514631
C	8.088207	7.819706	-2.427704
C	7.947285	8.698661	-3.501232
C	7.343500	8.270093	-4.684207
C	6.888312	6.954583	-4.783539
C	7.037715	6.075656	-3.711769
C	7.514307	6.062210	2.490779
C	6.374378	5.880755	3.287854
C	6.215069	6.593465	4.477276
C	7.191089	7.501818	4.889783
C	8.328702	7.694157	4.103783
C	8.485577	6.982738	2.913596
C	9.149864	3.928780	-2.666809
C	8.735656	2.813106	-3.411692
C	9.462872	2.390506	-4.526020
C	10.618547	3.069657	-4.913731
C	11.042191	4.179521	-4.180404
C	10.315667	4.601882	-3.067704
C	9.022961	3.528669	2.297453
C	10.423691	3.450370	2.372254
C	11.054001	2.862682	3.468280
C	10.292659	2.333669	4.512072
C	8.900401	2.394813	4.444704
C	8.270757	2.981506	3.345892
C	7.824644	2.153136	-0.278579
C	6.449027	2.110954	-0.016703
C	5.744370	0.909947	-0.067968
C	6.387581	-0.296694	-0.379439
C	7.764285	-0.254437	-0.638189
C	8.468985	0.947961	-0.582442
C	-6.367412	-2.139116	2.891659
C	-7.768132	-2.106612	2.945680
C	-8.428912	-1.616623	4.071919
C	-7.699141	-1.147152	5.164868
C	-6.304497	-1.175145	5.124160
C	-5.645608	-1.668185	3.998098
C	-4.870575	-4.646123	3.014067
C	-3.684230	-4.747868	3.755098
C	-3.646372	-5.467085	4.950034
C	-4.795357	-6.105713	5.420839
C	-5.980095	-6.019189	4.687748

C	-6.016924	-5.293288	3.497434
C	-3.434114	-4.010313	-1.778177
C	-3.838641	-5.103835	-2.556338
C	-3.086068	-5.513373	-3.658251
C	-1.908716	-4.842315	-3.994530
C	-1.495195	-3.752901	-3.226426
C	-2.254586	-3.339332	-2.131714
C	-3.449185	-5.603846	0.645470
C	-4.118989	-6.833612	0.617089
C	-3.410740	-8.035448	0.590458
C	-2.010693	-8.051254	0.600755
C	-1.341818	-6.822021	0.673544
C	-2.047866	-5.621106	0.683967
C	-1.244847	-9.381386	0.521530
C	-0.201084	-9.536883	1.623196
C	1.119939	-9.508716	1.361412
C	1.665640	-9.332654	-0.058828
C	0.639466	-9.635242	-1.139272
C	-0.682524	-9.653924	-0.878818
C	5.637193	-1.595898	-0.405222
C	5.471488	-2.299798	-1.619267
C	4.802941	-3.545479	-1.631212
C	4.299288	-4.087363	-0.428488
C	4.433916	-3.367548	0.779523
C	5.103907	-2.124031	0.791920
C	6.000511	-1.722891	-2.899400
C	5.446301	-0.552649	-3.439515
C	5.918291	-0.026364	-4.642219
C	6.960740	-0.658386	-5.323801
C	7.528021	-1.817412	-4.790978
C	7.049378	-2.345070	-3.591949
C	5.251296	-1.374579	2.083416
C	4.131127	-0.827169	2.726061
C	4.262249	-0.150373	3.938941
C	5.519007	-0.010066	4.531648
C	6.641543	-0.548426	3.900343
C	6.507724	-1.221546	2.686267
C	4.612880	-4.288836	-2.920406
C	3.776562	-3.775645	-3.922697
C	3.585296	-4.467975	-5.118256
C	4.230783	-5.686492	-5.334192
C	5.067990	-6.206235	-4.345923

C	5.255056	-5.514258	-3.149606
C	3.851837	-3.916036	2.049858
C	4.675052	-4.319948	3.110521
C	4.126206	-4.803621	4.297863
C	2.740918	-4.889691	4.447978
C	1.909928	-4.496350	3.397139
C	2.461826	-4.016149	2.209520
C	3.636315	-5.433010	-0.410196
C	2.378874	-5.649255	-0.986252
C	1.753370	-6.894039	-0.900478
C	2.362022	-7.968867	-0.241384
C	3.645006	-7.765334	0.286397
C	4.264047	-6.520207	0.214160
C	-0.756707	-9.864586	2.976290
C	-0.528425	-11.136168	3.528777
C	-1.082040	-11.498068	4.756070
C	-1.882716	-10.595128	5.457653
C	-2.121157	-9.329938	4.920103
C	-1.567063	-8.967467	3.691334
C	-1.698158	-10.091414	-1.890116
C	-1.798225	-11.452842	-2.219275
C	-2.761604	-11.904954	-3.120703
C	-3.647749	-11.001760	-3.710082
C	-3.559523	-9.646248	-3.390723
C	-2.596892	-9.193440	-2.486506
C	2.151095	-9.686497	2.429952
C	2.297832	-8.748169	3.461623
C	3.278217	-8.910241	4.440471
C	4.130214	-10.014988	4.409224
C	3.997554	-10.956761	3.386790
C	3.021622	-10.789163	2.404462
C	1.206196	-9.932429	-2.492405
C	0.852419	-9.177373	-3.621364
C	1.402609	-9.455740	-4.872035
C	2.318285	-10.497910	-5.023181
C	2.680211	-11.258962	-3.910665
C	2.134215	-10.973848	-2.659624
H	-8.132455	-1.536410	-0.693176
H	-9.425291	0.549311	-0.875790
H	-6.343557	2.644626	1.252946
H	-5.074194	0.552012	1.478768
H	-5.843998	7.966985	-0.013851

H	-5.475459	3.725458	-0.559831
H	-3.390078	8.174997	0.033433
H	-3.026623	3.926121	-0.456849
H	3.023341	7.853399	1.227094
H	2.947553	4.573698	-1.539770
H	5.482746	7.802726	1.217828
H	5.404113	4.483601	-1.502292
H	-5.205512	-6.851431	0.607546
H	-1.502773	-4.681446	0.716216
H	-3.956389	-8.974914	0.553390
H	-0.258696	-6.800748	0.725550
H	5.922860	3.022377	0.247512
H	9.537653	0.948271	-0.781906
H	4.680199	0.906453	0.150924
H	8.291891	-1.172821	-0.879970
H	1.876319	-4.837049	-1.504344
H	5.248153	-6.387373	0.655323
H	0.775343	-7.022920	-1.352009
H	4.162094	-8.584649	0.776769
H	-5.844669	5.887753	2.642820
H	-9.997407	6.734001	1.934052
H	-5.808100	7.575564	4.444834
H	-9.962647	8.431496	3.736032
H	-7.862850	8.861257	5.002706
H	-11.228948	3.418309	1.535318
H	-7.437910	3.545797	3.554982
H	-12.304965	2.602007	3.598177
H	-8.510453	2.721350	5.620247
H	-10.954040	2.246300	5.658858
H	-6.341464	4.113983	-3.637593
H	-8.888766	7.311260	-2.318948
H	-6.024330	5.260331	-5.805605
H	-8.557807	8.469613	-4.471208
H	-7.126444	7.450742	-6.234374
H	-7.338333	1.730741	-2.941632
H	-10.875831	4.137272	-2.537009
H	-8.348239	0.560500	-4.858708
H	-11.899291	2.959811	-4.459636
H	-10.639622	1.162674	-5.634494
H	-0.896733	3.035557	1.731231
H	-2.396370	6.924039	2.760436
H	-2.131752	2.049972	3.634434

H	-3.609174	5.949667	4.685509
H	-3.495358	3.503668	5.124913
H	2.168971	3.453886	1.139417
H	0.712817	6.763026	3.454403
H	3.404299	2.611251	3.109987
H	1.953460	5.935071	5.426771
H	3.306427	3.850689	5.266662
H	2.171641	8.974322	-1.366347
H	6.411992	6.609445	-5.697473
H	3.419605	9.841478	-3.315644
H	1.944907	6.582387	-5.703372
H	3.318316	8.648577	-5.495605
H	-2.412910	5.496512	-3.096345
H	-0.918404	9.351490	-1.938052
H	-3.663982	6.537487	-4.956317
H	-2.169702	10.399337	-3.796237
H	-3.549124	8.994484	-5.320088
H	5.602784	5.185140	2.972371
H	9.376910	7.129625	2.308465
H	5.321476	6.437243	5.075925
H	9.097294	8.395875	4.417918
H	7.067109	8.055999	5.816617
H	11.026298	3.873774	1.572004
H	7.187530	3.011121	3.296770
H	12.139605	2.823908	3.509365
H	8.297514	1.986006	5.251417
H	10.780864	1.878379	5.369839
H	8.570540	8.167736	-1.517632
H	6.681687	5.054521	-3.800380
H	8.311568	9.719067	-3.412391
H	0.690425	5.726153	-3.751962
H	7.229210	8.954963	-5.520414
H	7.838493	2.274024	-3.125332
H	10.646709	5.468030	-2.501436
H	9.120611	1.523760	-5.085029
H	11.939667	4.718248	-4.473944
H	11.184410	2.737789	-5.780475
H	3.148985	-0.944190	2.277135
H	7.386852	-1.631702	2.197781
H	3.380041	0.259358	4.424328
H	7.625623	-0.438562	4.346643
H	5.620723	0.510893	5.480332

H	5.753528	-4.245670	3.006122
H	1.810249	-3.711987	1.395301
H	4.783063	-5.108355	5.108475
H	0.829671	-4.560219	3.501566
H	2.312056	-5.256489	5.377038
H	3.276695	-2.824400	-3.763212
H	5.899760	-5.930222	-2.380885
H	2.932101	-4.053284	-5.881896
H	5.572737	-7.155808	-4.502774
H	4.082246	-6.227606	-6.264898
H	4.637034	-0.054876	-2.912812
H	7.487912	-3.253770	-3.189793
H	5.467762	0.875088	-5.049721
H	8.340763	-2.316597	-5.312107
H	7.321947	-0.254711	-6.266219
H	1.648805	-7.878626	3.484244
H	2.922879	-11.532836	1.616619
H	3.380763	-8.163653	5.223320
H	4.652526	-11.823812	3.354293
H	4.893361	-10.140658	5.172827
H	0.090529	-11.844417	2.985955
H	-1.763829	-7.978958	3.291210
H	-0.889368	-12.487948	5.162102
H	-2.747080	-8.617152	5.450587
H	-2.317703	-10.876284	6.413331
H	0.143135	-8.362919	-3.515354
H	2.417582	-11.583349	-1.804959
H	1.115950	-8.853186	-5.730074
H	3.387294	-12.077996	-4.015539
H	2.745817	-10.715522	-5.998445
H	-1.107565	-12.158196	-1.765259
H	-2.536141	-8.135903	-2.251528
H	-2.818175	-12.963047	-3.363372
H	-4.244424	-8.935075	-3.846527
H	-4.399275	-11.351384	-4.413186
H	-2.787197	-4.253275	3.393400
H	-6.944910	-5.221017	2.937527
H	-2.719763	-5.523984	5.515645
H	-6.879704	-6.513681	5.044691
H	-4.769178	-6.661761	6.354425
H	-8.343149	-2.463185	2.096103
H	-4.559720	-1.690159	3.974439

H	-9.515225	-1.595419	4.091854
H	-5.726143	-0.812718	5.970253
H	-8.214042	-0.761125	6.040540
H	-4.748605	-5.636515	-2.294463
H	-1.931384	-2.486108	-1.541669
H	-3.419665	-6.358135	-4.255510
H	-0.581521	-3.221877	-3.480978
H	-1.320990	-5.163549	-4.850270
H	-6.204563	-3.037057	-2.993935
H	-3.842225	0.056876	-1.184678
H	-6.252702	-1.766507	-5.115044
H	-3.875615	1.329382	-3.304677
H	-5.084720	0.425683	-5.285074
H	9.675507	3.167043	-0.232299
H	7.489185	7.106222	0.075443
H	2.468514	-10.075435	-0.168530
H	-1.997212	-10.162641	0.698990
H	-7.748516	6.739059	-0.404951
H	-9.810475	2.770970	-0.099396

Structure 9: Atom numer: 364

Total energy (Hartree): -8320.10984775

C	0.405604	0.072351	-0.636821
C	0.265107	-0.132485	0.736874
C	1.390452	-0.150309	1.561566
C	2.673962	0.036270	1.030652
C	2.800937	0.264873	-0.347167
C	1.677932	0.278526	-1.175060
C	3.882742	-0.095094	1.911114
C	4.611914	1.036353	2.333733
C	5.707798	0.878631	3.210651
C	6.149984	-0.415678	3.565047
C	5.480529	-1.549413	3.058144
C	4.311141	-1.387556	2.283991
C	4.366233	2.367418	1.681460
C	3.333068	3.259337	1.990477
C	3.134688	4.427479	1.243012
C	3.954145	4.730865	0.146258
C	5.018677	3.850613	-0.126240
C	5.225874	2.705733	0.626369
C	3.742036	5.786285	-0.899798
C	4.844133	6.574975	-1.319748

C	4.894318	7.065178	-2.643557
C	3.849226	6.735013	-3.538119
C	2.618686	6.276740	-3.005093
C	2.557480	5.812422	-1.674277
C	5.941848	6.857313	-0.340498
C	5.620470	7.435884	0.898165
C	6.609973	7.719502	1.838666
C	7.945039	7.423832	1.561429
C	8.279502	6.843073	0.336937
C	7.289122	6.564665	-0.603678
C	1.243848	5.385675	-1.085772
C	0.871831	4.045178	-0.921429
C	-0.362919	3.713759	-0.362270
C	-1.245392	4.715948	0.043672
C	-0.885115	6.055038	-0.112459
C	0.349942	6.385037	-0.671149
C	6.014810	7.963604	-3.080069
C	7.040985	7.558041	-3.943582
C	8.047721	8.448849	-4.319210
C	8.045560	9.759295	-3.839753
C	7.026872	10.176419	-2.981961
C	6.020721	9.285970	-2.608436
C	1.401814	6.295406	-3.878872
C	0.650871	5.145180	-4.164079
C	-0.485196	5.213624	-4.968813
C	-0.897023	6.435374	-5.503634
C	-0.156498	7.586836	-5.235081
C	0.982800	7.514897	-4.434311
C	4.106974	6.545637	-5.005793
C	4.568290	7.476445	-5.946321
C	4.920891	7.077539	-7.243756
C	4.813386	5.740777	-7.645188
C	4.317995	4.817821	-6.711728
C	3.980702	5.208646	-5.426921
C	5.214601	5.153058	-8.969074
C	6.566206	4.857766	-9.241759
C	6.894711	3.974692	-10.294760
C	5.869842	3.357150	-11.046806
C	4.529837	3.760535	-10.862216
C	4.205960	4.674370	-9.835191
C	7.670172	5.434357	-8.403764
C	7.978103	4.903079	-7.142982

C	9.071138	5.377426	-6.417206
C	9.866768	6.402389	-6.934813
C	9.551830	6.958643	-8.175468
C	8.463859	6.475947	-8.903435
C	2.790757	5.126774	-9.632017
C	1.785824	4.251511	-9.196596
C	0.477496	4.698770	-9.019434
C	0.148207	6.029909	-9.279497
C	1.138968	6.912070	-9.711283
C	2.449121	6.464219	-9.880807
C	8.330469	3.684842	-10.622414
C	9.161811	2.962556	-9.754170
C	10.495954	2.720006	-10.078395
C	11.028084	3.202130	-11.275909
C	10.210856	3.920608	-12.150366
C	8.873475	4.153685	-11.827534
C	3.430883	3.194569	-11.712653
C	2.798549	3.991917	-12.676887
C	1.736915	3.493634	-13.432324
C	1.284759	2.188811	-13.230107
C	1.912444	1.381619	-12.278479
C	2.980666	1.879035	-11.531319
C	6.205785	2.184296	-11.919937
C	6.069279	2.167322	-13.312944
C	6.371598	1.017551	-14.047893
C	6.805528	-0.155729	-13.418421
C	6.957411	-0.132296	-12.026581
C	6.664038	1.012862	-11.293767
C	7.096562	-1.435307	-14.219671
C	5.978540	-2.473662	-14.083617
C	6.155201	-3.650655	-13.448561
C	7.502390	-4.086824	-12.891258
C	8.663392	-3.180513	-13.298151
C	8.481154	-1.998870	-13.918213
C	4.733098	-2.161729	-14.857902
C	4.472389	-2.883258	-16.035386
C	3.361101	-2.594361	-16.826041
C	2.487039	-1.570540	-16.456984
C	2.733894	-0.844659	-15.291531
C	3.846382	-1.134966	-14.499390
C	9.619601	-1.204164	-14.486862
C	10.033546	0.021546	-13.942868

C	11.067123	0.749195	-14.535225
C	11.699149	0.273814	-15.684682
C	11.292894	-0.941173	-16.238753
C	10.263075	-1.670256	-15.645059
C	5.051765	-4.651694	-13.303292
C	3.845938	-4.309026	-12.671692
C	2.816605	-5.239251	-12.533350
C	2.971663	-6.537860	-13.021770
C	4.167811	-6.897223	-13.644607
C	5.196873	-5.965501	-13.778059
C	10.029220	-3.730299	-13.028723
C	10.456824	-4.918263	-13.643641
C	11.728528	-5.437235	-13.399650
C	12.597164	-4.780377	-12.526501
C	12.182631	-3.602084	-11.903814
C	10.911178	-3.083772	-12.150513
C	7.482803	-4.395555	-11.383447
C	6.843688	-3.570923	-10.449871
C	6.869777	-3.867134	-9.085785
C	7.516802	-5.013949	-8.606951
C	8.140810	-5.848746	-9.543576
C	8.126645	-5.542248	-10.902174
C	6.413164	2.095291	3.729954
C	5.762125	2.953511	4.628017
C	6.401241	4.090390	5.122725
C	7.702580	4.392925	4.720437
C	8.359272	3.549605	3.822907
C	7.721077	2.410015	3.335037
C	7.350345	-0.578470	4.450115
C	8.578383	-1.010270	3.928864
C	9.700643	-1.128229	4.749424
C	9.609611	-0.829244	6.110357
C	8.389667	-0.409370	6.642582
C	7.271723	-0.281124	5.817988
C	3.539435	-2.598801	1.846001
C	2.786611	-3.326889	2.778280
C	2.062157	-4.454487	2.389279
C	2.087357	-4.880972	1.060189
C	2.836277	-4.166302	0.123484
C	3.551360	-3.034154	0.513380
C	6.049145	-2.926818	3.229608
C	6.020237	-3.637167	4.434600

C	6.544958	-4.929589	4.513459
C	7.121272	-5.549058	3.397627
C	7.169155	-4.828949	2.197362
C	6.637096	-3.546392	2.115302
C	7.696398	-6.970670	3.486588
C	9.142309	-7.034637	3.002972
C	9.479379	-7.629363	1.841918
C	8.458856	-8.369917	0.974762
C	7.149689	-8.660699	1.693649
C	6.795504	-8.016740	2.823000
C	7.546343	-5.372192	-7.147727
C	6.775067	-6.458722	-6.675949
C	6.849603	-6.842584	-5.317963
C	7.727249	-6.167684	-4.440587
C	8.478446	-5.066721	-4.905792
C	8.375266	-4.657860	-6.255365
C	5.902476	-7.222714	-7.627005
C	4.803278	-6.615234	-8.250596
C	4.013222	-7.322198	-9.156356
C	4.307415	-8.653543	-9.453472
C	5.395018	-9.272432	-8.835596
C	6.184214	-8.562841	-7.930959
C	9.146181	-3.464618	-6.737954
C	8.816961	-2.178770	-6.284263
C	9.523256	-1.060498	-6.727161
C	10.576179	-1.209060	-7.631200
C	10.914611	-2.483413	-8.088935
C	10.203917	-3.599769	-7.648561
C	5.974092	-7.947475	-4.803697
C	4.587149	-7.756584	-4.719149
C	3.754188	-8.764100	-4.233025
C	4.295069	-9.983266	-3.822836
C	5.673285	-10.186781	-3.904541
C	6.504395	-9.177881	-4.390487
C	9.401128	-4.346797	-3.966902
C	10.791816	-4.416214	-4.133989
C	11.651920	-3.754022	-3.258284
C	11.135789	-3.004450	-2.200060
C	9.753496	-2.925540	-2.023612
C	8.895550	-3.593113	-2.897680
C	7.906858	-6.663995	-3.033287
C	6.958906	-6.441995	-2.028123

C	7.133838	-6.961505	-0.743053
C	8.255187	-7.734689	-0.418116
C	9.203218	-7.955896	-1.427667
C	9.040465	-7.419954	-2.702596
C	10.159895	-6.507368	3.968973
C	11.075127	-7.399497	4.553947
C	11.995801	-6.966719	5.506953
C	12.017817	-5.628393	5.902651
C	11.112509	-4.731775	5.334737
C	10.191496	-5.164563	4.379235
C	5.565540	-8.366051	3.604433
C	5.541589	-9.542445	4.370200
C	4.428181	-9.873232	5.142301
C	3.316150	-9.029758	5.165577
C	3.328551	-7.856481	4.410425
C	4.443671	-7.523219	3.639580
C	10.889558	-7.659352	1.343566
C	11.541818	-6.476935	0.964959
C	12.846579	-6.505500	0.472783
C	13.526827	-7.717975	0.351333
C	12.890321	-8.903956	0.723291
C	11.582659	-8.873706	1.208113
C	6.311243	-9.739856	1.080936
C	4.991755	-9.507050	0.664226
C	4.229740	-10.525167	0.092393
C	4.769489	-11.801426	-0.073825
C	6.080952	-12.049503	0.334132
C	6.842997	-11.027712	0.899883
H	2.655891	3.036086	2.810497
H	2.311609	5.084871	1.502806
H	5.668472	4.052799	-0.971122
H	6.045860	2.038934	0.373367
H	4.678370	8.519302	-5.666213
H	3.639387	4.461976	-4.717089
H	5.296526	7.821280	-7.941126
H	4.221626	3.773715	-6.996528
H	5.719766	3.055847	-13.831005
H	6.789095	1.001545	-10.214773
H	6.255864	1.032367	-15.128672
H	7.329600	-1.011622	-11.511447
H	5.585470	-3.180231	5.319267
H	6.678250	-3.010809	1.170620

H	6.505740	-5.463518	5.459586
H	7.635145	-5.268832	1.321648
H	6.300576	-2.694792	-10.790454
H	8.626786	-6.208742	-11.599643
H	6.371870	-3.201913	-8.385192
H	8.638369	-6.753123	-9.204272
H	6.060693	-5.873142	-2.253584
H	9.794790	-7.609941	-3.461713
H	6.370573	-6.777690	0.006408
H	10.081089	-8.559635	-1.218023
H	7.050148	6.544767	-4.333312
H	5.230425	9.612167	-1.937837
H	8.826516	8.114935	-4.997866
H	7.012881	11.195054	-2.602653
H	8.830465	10.451053	-4.134642
H	4.582443	7.668390	1.118911
H	7.563538	6.112247	-1.550981
H	6.335869	8.170140	2.789043
H	9.315635	6.603411	0.111947
H	8.718304	7.642684	2.293145
H	0.959053	4.189202	-3.753593
H	1.554587	8.414696	-4.224561
H	-1.050717	4.308892	-5.176869
H	-0.464609	8.543804	-5.648369
H	-1.786613	6.488733	-6.125727
H	1.554362	3.254808	-1.218463
H	0.627323	7.428487	-0.794417
H	-0.625054	2.667608	-0.237309
H	-1.563618	6.844825	0.199920
H	-2.206372	4.455114	0.479680
H	7.366231	4.102197	-6.737756
H	8.236942	6.895464	-9.879642
H	9.307663	4.939350	-5.450752
H	10.160503	7.760800	-8.584511
H	10.727545	6.761435	-6.376444
H	8.760037	2.588852	-8.817384
H	8.241731	4.711891	-12.513114
H	11.122616	2.157237	-9.391194
H	10.613244	4.303454	-13.084819
H	12.070780	3.021174	-11.523349
H	3.133270	5.015076	-12.823201
H	2.065577	-0.041957	-14.993230

H	1.257105	4.129203	-14.171959
H	1.567198	0.364164	-12.113935
H	0.445833	1.805046	-13.804951
H	2.031298	3.213129	-8.995647
H	3.218425	7.154758	-10.216320
H	-0.285474	4.005726	-8.675048
H	0.894343	7.951530	-9.914429
H	-0.872243	6.377542	-9.143024
H	9.554733	0.406741	-13.048860
H	9.951365	-2.617034	-16.077694
H	11.374039	1.691592	-14.089867
H	11.777976	-1.323812	-17.133248
H	12.502474	0.843868	-16.144443
H	9.795254	-5.431911	-14.337586
H	10.592680	-2.170586	-11.657796
H	12.042056	-6.351978	-13.896372
H	12.853324	-3.080759	-11.225139
H	13.588464	-5.182551	-12.334809
H	5.150661	-3.680551	-16.326253
H	4.020121	-0.560483	-13.596014
H	3.179897	-3.168983	-17.730910
H	3.464846	1.247355	-10.791837
H	1.621300	-1.340849	-17.072897
H	3.718627	-3.301402	-12.288887
H	6.116130	-6.260426	-14.277923
H	1.890255	-4.946969	-12.045031
H	4.300579	-7.904373	-14.031375
H	2.167908	-7.262348	-12.919405
H	8.002725	-2.057698	-5.575320
H	10.468168	-4.585804	-8.018573
H	9.251844	-0.073038	-6.362152
H	11.730938	-2.613178	-8.794446
H	11.129214	-0.339018	-7.976010
H	11.200425	-4.993045	-4.958690
H	7.820828	-3.531120	-2.752070
H	12.726700	-3.822970	-3.405170
H	9.340695	-2.343591	-1.203259
H	11.805095	-2.484278	-1.519645
H	4.160559	-6.810371	-5.040492
H	7.576399	-9.342428	-4.443297
H	2.681840	-8.594830	-4.175461
H	6.104394	-11.129801	-3.579578

H	3.649325	-10.768395	-3.439141
H	4.572779	-5.576893	-8.031134
H	7.030168	-9.050192	-7.454904
H	3.177557	-6.826878	-9.642516
H	5.633454	-10.309134	-9.059570
H	3.696204	-9.202354	-10.165042
H	11.013677	-5.532109	1.047976
H	11.099438	-9.803092	1.501524
H	13.328894	-5.577483	0.177243
H	13.412349	-9.853732	0.638330
H	14.543808	-7.739937	-0.031335
H	11.058186	-8.443596	4.256066
H	9.498519	-4.450656	3.948451
H	12.693065	-7.677590	5.943220
H	11.111118	-3.688457	5.638328
H	12.732106	-5.288880	6.648382
H	4.561364	-8.518910	0.788946
H	7.858094	-11.240877	1.226102
H	3.211436	-10.318037	-0.225835
H	6.511522	-13.040851	0.216994
H	4.173545	-12.596065	-0.515306
H	6.403447	-10.204025	4.350794
H	4.435994	-6.609036	3.054402
H	4.429566	-10.791496	5.724134
H	2.466854	-7.193718	4.417273
H	2.446966	-9.286266	5.765741
H	8.654334	-1.248236	2.871727
H	6.328342	0.060686	6.234674
H	10.647235	-1.450101	4.322820
H	8.308195	-0.174030	7.700544
H	10.483607	-0.918370	6.750294
H	4.747622	2.723034	4.942139
H	8.240864	1.756916	2.640398
H	5.880069	4.740597	5.820718
H	9.371228	3.779070	3.499698
H	8.201027	5.280129	5.101860
H	2.767907	-3.003146	3.815411
H	4.125924	-2.479872	-0.222947
H	1.474166	-4.997234	3.125285
H	2.861823	-4.488737	-0.914249
H	1.525650	-5.760321	0.756505
H	1.276720	-0.331220	2.626816

H	3.787931	0.418626	-0.773469
H	-0.720323	-0.291250	1.167274
H	1.798448	0.438279	-2.243679
H	-0.467860	0.068241	-1.283694
H	7.716079	-5.058601	-13.362710
H	7.106578	-1.129772	-15.275833
H	8.916455	-9.346924	0.759381
H	7.709846	-7.220139	4.556709

Structure 3: Atom numer: 364
Total energy (Hartree): -8320.144625

C	19.303699	29.996594	6.737405
C	20.141305	31.069678	6.356123
C	21.522757	30.850937	6.155579
C	22.073851	29.566954	6.357939
C	21.259482	28.522519	6.843806
C	19.875448	28.735260	7.026435
C	19.584005	32.448879	6.160783
C	18.765856	32.764858	5.067890
H	18.496835	31.989362	4.356531
C	18.292142	34.064151	4.886945
H	17.667722	34.288271	4.027372
C	18.614496	35.066665	5.802741
C	19.416702	34.760462	6.902921
H	19.672718	35.531910	7.624660
C	19.900135	33.463741	7.076283
H	20.536261	33.232987	7.926369
C	22.413158	31.970256	5.701915
C	22.268127	32.544229	4.429954
H	21.486814	32.182737	3.767782
C	23.112430	33.571069	4.008140
H	22.983601	34.001253	3.018285
C	24.115562	34.048231	4.854155
C	24.268651	33.487720	6.123366
H	25.044101	33.852929	6.791871
C	23.426704	32.456237	6.540358
H	23.554640	32.020882	7.527401
C	21.864027	27.183040	7.152906
C	22.028799	26.768620	8.481976
H	21.708563	27.423613	9.287440
C	22.593217	25.527710	8.779057
H	22.713558	25.225912	9.816339

C	22.998262	24.676224	7.750178
C	22.838415	25.076442	6.422092
H	23.146418	24.418698	5.612701
C	22.279173	26.320453	6.128721
H	22.166699	26.632871	5.094793
C	19.024479	27.610424	7.539277
C	18.502284	27.655362	8.839267
H	18.689268	28.528329	9.458510
C	17.761570	26.587902	9.347810
H	17.391020	26.630543	10.368467
C	17.508617	25.466342	8.556217
C	18.008894	25.417427	7.253469
H	17.813625	24.552798	6.624580
C	18.767214	26.476432	6.755315
H	19.169579	26.423051	5.747747
C	17.810479	30.151645	6.731570
C	17.064861	29.393669	5.815522
H	17.577036	28.702482	5.153123
C	15.682234	29.512676	5.736585
H	15.136982	28.907814	5.018369
C	14.978084	30.396026	6.569234
C	15.720295	31.155263	7.484824
H	15.209495	31.843674	8.151419
C	17.109710	31.036240	7.563525
H	17.653246	31.646517	8.278531
C	13.482200	30.444190	6.453400
C	12.661611	30.008437	7.519054
C	11.271742	29.848623	7.320449
C	10.695485	30.149903	6.067112
C	11.495826	30.688785	5.038137
C	12.887904	30.829159	5.228889
C	13.243638	29.720959	8.871559
C	14.058819	28.605367	9.105199
H	14.309887	27.941383	8.283091
C	14.552850	28.341558	10.382674
H	15.173513	27.465176	10.542681
C	14.254841	29.195684	11.445343
H	14.642179	28.988736	12.439614
C	13.456585	30.318249	11.221490
H	13.219664	30.992967	12.040151
C	12.952719	30.574387	9.946587
H	12.319983	31.441772	9.779935

C	10.398522	29.332937	8.426371
C	9.406760	30.148776	8.989561
H	9.283857	31.164746	8.625250
C	8.579667	29.673055	10.007550
H	7.821444	30.325039	10.433710
C	8.725632	28.366480	10.476734
H	8.084028	27.994344	11.271308
C	9.707228	27.542614	9.922351
H	9.830607	26.524001	10.281094
C	10.537203	28.023034	8.909646
H	11.301633	27.376906	8.487990
C	10.868613	31.095577	3.735699
C	10.422507	30.135165	2.817032
H	10.527258	29.081651	3.058972
C	9.843842	30.515823	1.605963
H	9.511441	29.755210	0.903420
C	9.695589	31.869086	1.295864
H	9.246265	32.168062	0.352409
C	10.131718	32.835022	2.203786
H	10.020913	33.891198	1.971947
C	10.715205	32.450828	3.411601
H	11.059701	33.208002	4.110459
C	13.722393	31.406225	4.123232
C	13.940433	30.702408	2.930066
H	13.520946	29.707174	2.814010
C	14.680353	31.265467	1.890703
H	14.843746	30.698074	0.978245
C	15.202154	32.554195	2.020316
H	15.763245	33.002944	1.204548
C	14.989439	33.265923	3.202167
H	15.377930	34.275138	3.309369
C	14.266562	32.691946	4.248389
H	14.109863	33.249416	5.167777
C	9.261421	29.802096	5.798890
C	8.247336	30.755776	5.644240
H	8.482418	31.812341	5.738398
C	6.935362	30.367180	5.371183
H	6.168777	31.130128	5.271646
C	6.593467	29.014999	5.215327
C	7.607627	28.064582	5.375839
H	7.379844	27.009761	5.271631
C	8.911907	28.452256	5.671611

H	9.677700	27.693254	5.801536
C	5.141245	28.612595	4.879715
H	4.509748	29.280370	5.478921
C	4.795266	28.894976	3.410733
C	4.736043	27.896500	2.503679
C	5.087813	26.466868	2.904610
H	4.425377	25.808166	2.329360
C	4.792555	26.189293	4.385542
C	4.784856	27.188115	5.293812
C	4.531845	30.327625	3.079675
C	3.540731	31.054466	3.762510
H	2.933289	30.558836	4.516109
C	3.306355	32.398366	3.471944
H	2.525984	32.935985	4.004550
C	4.069992	33.049832	2.501451
C	5.064925	32.343831	1.823259
H	5.672345	32.842590	1.072600
C	5.292426	30.997930	2.108511
H	6.073374	30.459108	1.581212
C	4.237888	28.071976	1.104169
C	2.998616	28.693483	0.867309
H	2.430874	29.079393	1.707860
C	2.487498	28.814686	-0.423524
H	1.525610	29.297648	-0.576299
C	3.202294	28.313985	-1.513410
C	4.428777	27.686199	-1.294849
H	5.000003	27.291918	-2.131511
C	4.938504	27.559028	-0.002149
H	5.895302	27.069249	0.142026
C	4.517931	24.761579	4.728294
C	5.299558	24.081574	5.675793
H	6.105949	24.609505	6.175002
C	5.060099	22.740360	5.972823
H	5.683910	22.233806	6.704588
C	4.032064	22.048802	5.330051
C	3.247155	22.709948	4.383353
H	2.441072	22.183489	3.878624
C	3.493030	24.049217	4.081091
H	2.868827	24.552752	3.346687
C	4.340397	27.019565	6.712260
C	5.103211	27.501465	7.790946
H	6.068870	27.960512	7.609326

C	4.643858	27.382158	9.103124
H	5.262147	27.751765	9.917300
C	3.407345	26.793554	9.369206
C	2.631561	26.324195	8.307479
H	1.661458	25.871990	8.497861
C	3.092296	26.437260	6.996999
H	2.477613	26.075893	6.178731
C	6.520843	26.044259	2.514902
C	6.839326	24.687157	2.351201
H	6.068325	23.934062	2.485371
C	8.133844	24.280862	2.025282
H	8.351390	23.221072	1.925285
C	9.152629	25.220840	1.824629
C	8.825757	26.575393	1.961217
H	9.595122	27.324016	1.796939
C	7.539740	26.980811	2.308590
H	7.330059	28.038986	2.416710
C	10.570156	24.855699	1.497657
C	11.407146	24.310245	2.493588
C	12.789158	24.158807	2.246823
C	13.336277	24.538134	0.998622
C	12.476675	24.979583	-0.033275
C	11.097364	25.150848	0.221473
C	10.829858	23.907106	3.819973
C	10.430418	24.869108	4.758273
H	10.535641	25.922329	4.515082
C	9.897047	24.490405	5.990495
H	9.600727	25.252110	6.707819
C	9.748044	23.137749	6.302776
H	9.333521	22.840368	7.262520
C	10.138931	22.170252	5.376105
H	10.027678	21.114524	5.609741
C	13.664727	23.580869	3.319899
C	10.677258	22.552439	4.146891
H	10.986413	21.794226	3.432764
C	13.932475	24.288028	4.500996
H	13.519606	25.284331	4.630740
C	14.714467	23.727932	5.510665
H	14.916693	24.298342	6.413421
C	15.229215	22.438256	5.363847
H	15.823626	21.991697	6.156895
C	14.966678	21.722823	4.194336

H	15.349204	20.712710	4.074731
C	14.201622	22.293980	3.176886
H	14.007155	21.733721	2.266464
C	13.006919	25.265042	-1.407363
C	12.670691	24.414731	-2.471470
H	12.040479	23.550217	-2.281776
C	13.126569	24.670431	-3.764450
H	12.855166	23.998048	-4.574281
C	13.921175	25.789434	-4.017377
H	14.271201	25.995824	-5.025499
C	14.263580	26.640776	-2.965921
H	14.882008	27.514392	-3.148491
C	13.817816	26.377190	-1.670808
H	14.103523	27.038707	-0.858090
C	10.184369	25.673185	-0.848626
C	9.163787	24.865205	-1.370178
H	9.047409	23.850482	-1.000257
C	8.300215	25.347303	-2.354304
H	7.519882	24.701497	-2.748814
C	8.438143	26.652171	-2.830557
H	7.767948	27.029020	-3.598874
C	9.448222	27.468212	-2.317372
H	9.565455	28.485471	-2.681939
C	10.314350	26.981519	-1.338512
H	11.100859	27.621279	-0.949009
C	14.826584	24.576879	0.823338
C	15.527902	23.813545	-0.120591
H	14.987362	23.129067	-0.767534
C	16.913991	23.923577	-0.252572
H	17.425790	23.310380	-0.988180
C	17.651971	24.802943	0.552359
C	16.947233	25.565290	1.496561
H	17.489078	26.252279	2.139406
C	15.567902	25.455782	1.628005
H	15.054624	26.064116	2.366499
C	19.144669	24.948167	0.488856
C	19.989850	23.868197	0.832206
C	21.379392	24.077940	0.979613
C	21.930307	25.359092	0.759833
C	21.103931	26.410545	0.311102
C	19.712620	26.207064	0.182245
C	19.433466	22.490375	1.039605

C	18.649957	22.170708	2.156586
H	18.406771	22.942494	2.881131
C	18.177016	20.872091	2.344698
H	17.579928	20.645022	3.222735
C	18.465262	19.873953	1.412914
C	19.232749	20.183827	0.289305
H	19.462174	19.415947	-0.445066
C	19.715563	21.479683	0.108374
H	20.324404	21.713159	-0.760702
C	22.279589	22.951842	1.395745
C	23.254843	22.459299	0.516675
H	23.346005	22.894913	-0.474321
C	24.105049	21.421017	0.898460
H	24.849943	21.050534	0.198835
C	23.999159	20.860382	2.172426
C	23.034988	21.344435	3.058851
H	22.943238	20.914381	4.052883
C	22.181987	22.377904	2.672229
H	21.430827	22.744559	3.365772
C	21.703647	27.748043	-0.015262
C	21.825130	28.165991	-1.347849
H	21.475592	27.514635	-2.144047
C	22.383292	29.406072	-1.659899
H	22.469813	29.710737	-2.699714
C	22.824863	30.253218	-0.642575
C	22.708515	29.849331	0.688940
H	23.045671	30.503796	1.489328
C	22.155531	28.606088	0.997095
H	22.075781	28.290863	2.033255
C	18.849741	27.338110	-0.296022
C	18.281517	27.299467	-1.576833
H	18.442693	26.427590	-2.204792
C	17.527661	28.371534	-2.055446
H	17.120295	28.333498	-3.062180
C	17.308486	29.491908	-1.252188
C	17.855191	29.534766	0.032012
H	17.686424	30.398293	0.670001
C	18.625762	28.470829	0.499888
H	19.063918	28.519470	1.492653
C	23.365025	25.626123	1.105735
C	24.364379	25.858602	0.152477
H	24.117175	25.831509	-0.905185

C	25.677438	26.124071	0.542756
H	26.432567	26.284202	-0.221258
C	26.035049	26.194499	1.898057
C	25.035340	25.956849	2.847819
H	25.274159	25.994776	3.904776
C	23.730209	25.668989	2.456814
H	22.975866	25.478077	3.214555
C	27.488254	26.521981	2.303423
H	28.117613	25.976662	1.589053
C	27.869423	26.019449	3.692383
C	27.865255	26.859882	4.749025
C	27.548441	28.351937	4.574691
H	28.214434	28.890565	5.260300
C	27.872858	28.850655	3.169838
C	27.812893	28.010471	2.114761
C	28.332721	24.598649	3.761345
C	27.576257	23.543965	3.219845
H	26.601979	23.742405	2.786584
C	28.053265	22.232869	3.246354
H	27.439636	21.436749	2.832324
C	29.301316	21.945015	3.799252
H	29.672990	20.923707	3.816045
C	30.070669	22.983699	4.327021
H	31.049445	22.776776	4.752487
C	29.592376	24.292560	4.306804
H	30.201889	25.093192	4.713501
C	28.166283	26.427314	6.146760
C	27.403355	25.431118	6.776322
H	26.591447	24.961204	6.230285
C	27.668524	25.049316	8.091018
H	27.059277	24.280755	8.559367
C	28.703974	25.654268	8.805313
H	28.910352	25.356317	9.829886
C	29.470266	26.648770	8.194376
H	30.281692	27.124787	8.739051
C	29.198520	27.035769	6.882240
H	29.807687	27.807024	6.416803
C	28.348492	30.266148	3.079878
C	27.628850	31.329085	3.654364
H	26.672833	31.141408	4.130984
C	28.119025	32.634683	3.606338
H	27.533589	33.437632	4.047185

C	29.344431	32.908550	2.998307
H	29.726528	33.925588	2.964823
C	30.077590	31.861443	2.436718
H	31.038575	32.057433	1.967747
C	29.585860	30.558087	2.478484
H	30.167222	29.750632	2.044902
C	28.056995	28.439561	0.704823
C	27.275821	29.441661	0.107916
H	26.492559	29.918844	0.688306
C	27.486541	29.819735	-1.217680
H	26.863907	30.592827	-1.660226
C	28.484912	29.205375	-1.975413
H	28.649014	29.500542	-3.008407
C	29.268812	28.205054	-1.397117
H	30.051742	27.721758	-1.976005
C	29.051081	27.821501	-0.073920
H	29.673052	27.045417	0.365807
C	26.117996	28.694469	5.043562
C	25.080059	28.942750	4.138527
H	25.272301	28.904217	3.072154
C	23.796047	29.242564	4.586275
H	23.011082	29.440193	3.862219
C	23.490614	29.288632	5.951980
C	24.528667	29.047114	6.860631
H	24.328538	29.076362	7.928149
C	25.820706	28.768632	6.413190
H	26.606735	28.601232	7.143714
H	3.893005	34.098713	2.278704
H	2.805061	28.409222	-2.520695
H	3.845768	21.003474	5.561750
H	3.049414	26.704408	10.391667
H	18.243091	36.078270	5.660787
H	24.768717	34.853662	4.528258
H	16.722705	30.327593	-1.626623
H	23.254291	31.221874	-0.884797
H	24.658609	20.049465	2.470871
H	18.094314	18.863017	1.560694
H	23.432900	23.707095	7.980941
H	16.932915	24.634382	8.953818

Structure 3a: Atom numer: 360

Total energy (Hartree): -8317.4881

C	3.231026	6.552191	0.482996
C	2.024690	7.234503	0.295143
C	0.820334	6.551896	0.033469
C	0.902840	5.145273	-0.036337
C	2.098239	4.468056	0.149661
C	3.296364	5.151807	0.422831
H	4.137245	7.122420	0.674392
H	2.019762	8.319083	0.355984
H	-0.003997	4.577015	-0.217363
H	2.108389	3.382844	0.093848
C	-0.529462	7.163801	-0.147876
C	-1.299216	6.803386	-1.297641
C	-1.173825	7.927124	0.859279
C	-2.691561	6.965723	-1.326839
C	-2.566044	8.139368	0.819285
C	-3.389001	7.505110	-0.190442
C	-4.820759	7.331631	0.014592
C	-5.815831	7.348484	-1.011843
C	-5.324003	6.947715	1.299941
C	-7.097146	6.888541	-0.823419
H	-5.559727	7.743126	-1.990260
C	-6.600492	6.483678	1.500890
H	-4.639839	6.930851	2.143491
C	-7.547458	6.312763	0.422688
H	-7.780206	6.930828	-1.664488
H	-6.864216	6.124787	2.489730
C	-8.718975	5.534464	0.551443
C	-9.432669	4.984843	-0.626578
C	-9.113759	4.889918	1.827259
C	-9.562684	3.606258	-0.655601
C	-9.300882	3.504429	1.789373
C	-9.255459	2.809282	0.522047
C	-8.783987	1.423923	0.359394
C	-9.110044	0.298235	1.160668
C	-7.818546	1.173326	-0.652589
C	-8.440309	-0.917942	1.041628
H	-9.892858	0.381961	1.905093
C	-7.180497	-0.048996	-0.796676
H	-7.505187	2.004114	-1.276649
C	-7.436081	-1.122375	0.078702

H	-8.699480	-1.726957	1.722194
H	-6.410849	-0.157004	-1.557834
C	-6.558443	-2.331898	0.010319
C	-5.655520	-2.618918	1.062533
C	-6.519090	-3.134767	-1.158520
C	-4.651572	-3.598347	0.893941
C	-5.513585	-4.116335	-1.326654
C	-4.529063	-4.302691	-0.328988
C	4.528097	4.316366	0.619508
C	5.576151	4.293797	-0.329430
C	4.565894	3.406495	1.704632
C	6.566967	3.284583	-0.270550
C	5.552337	2.397419	1.762762
C	6.525836	2.289233	0.740134
C	7.400743	1.078997	0.661738
C	8.327664	0.705265	1.650975
C	7.224669	0.175228	-0.404256
C	8.996050	-0.516200	1.610621
H	8.526387	1.383495	2.478812
C	7.860342	-1.056405	-0.424501
H	6.518528	0.418923	-1.195113
C	8.744058	-1.484721	0.603280
H	9.719190	-0.730161	2.388358
H	7.609221	-1.762265	-1.209590
C	-3.279668	-5.094012	-0.588003
C	-2.070650	-4.379869	-0.661149
C	-3.210392	-6.483843	-0.769203
C	-0.862907	-5.020446	-0.893844
H	-2.083083	-3.301853	-0.524583
C	-1.991762	-7.129070	-1.001693
H	-4.123367	-7.073950	-0.734622
C	-0.777779	-6.417707	-1.068943
H	0.050438	-4.434393	-0.918752
H	-1.984258	-8.207529	-1.131352
C	0.582030	-6.997960	-1.279249
C	1.172686	-7.927230	-0.385179
C	1.411145	-6.454297	-2.309170
C	2.564618	-8.145819	-0.391388
C	2.802879	-6.622322	-2.293864
C	3.440220	-7.356862	-1.234107
C	4.859896	-7.236412	-0.931208
C	5.299204	-7.092118	0.424989

C	5.905182	-7.079838	-1.894564
C	6.562713	-6.677946	0.766985
H	4.575424	-7.223270	1.224232
C	7.174124	-6.667838	-1.565184
H	5.699198	-7.294888	-2.938561
C	7.558818	-6.319839	-0.216955
H	6.778169	-6.505346	1.815708
H	7.898769	-6.565893	-2.365457
C	9.217154	-2.877707	0.561361
C	9.599202	-3.464930	-0.714976
C	9.198746	-3.775968	1.693762
C	9.478777	-4.827759	-0.925317
C	9.032225	-5.149513	1.488018
C	8.712865	-5.571404	0.102773
C	-3.429691	6.475782	-2.538775
C	-4.154246	5.274027	-2.506727
C	-3.381363	7.187991	-3.745450
C	-4.801833	4.797606	-3.645274
H	-4.222325	4.723155	-1.573818
C	-4.029302	6.713908	-4.888098
H	-2.823121	8.119724	-3.785587
C	-4.741861	5.515558	-4.842724
H	-5.361658	3.867024	-3.593665
H	-3.975036	7.282947	-5.814543
H	-5.250706	5.144365	-5.729792
C	-0.566715	6.308919	-2.512081
C	0.316727	7.175964	-3.176205
C	-0.726089	5.014799	-3.034204
C	0.994722	6.778106	-4.328462
H	0.461589	8.177189	-2.779190
C	-0.041852	4.608540	-4.179844
H	-1.400433	4.323167	-2.539754
C	0.819388	5.488560	-4.837568
H	1.657894	7.479265	-4.831910
H	-0.184060	3.599186	-4.559010
H	1.348145	5.172917	-5.734402
C	-3.209949	9.110251	1.758457
C	-3.092901	9.032650	3.158085
C	-3.944048	10.191872	1.232689
C	-3.660138	9.999921	3.989877
H	-2.555261	8.199765	3.599205
C	-4.508964	11.158828	2.058903

H	-4.072208	10.257190	0.156216
C	-4.367953	11.072138	3.448918
H	-3.554559	9.904058	5.068933
H	-5.067053	11.982686	1.617405
H	-4.817599	11.821487	4.097516
C	-0.336727	8.546388	1.940663
C	0.217651	7.776669	2.974178
C	-0.067973	9.923946	1.925275
C	1.003340	8.366588	3.965012
H	0.039431	6.705636	2.994262
C	0.715893	10.517137	2.915900
H	-0.494818	10.532288	1.132683
C	1.255040	9.739793	3.942494
H	1.422699	7.746018	4.753175
H	0.904257	11.588772	2.884965
H	1.868037	10.198627	4.715918
C	-10.080551	5.901091	-1.598579
C	-9.961429	5.785795	-3.001770
C	-10.844750	6.990918	-1.121489
C	-10.590147	6.681183	-3.866733
H	-9.345023	4.996222	-3.413953
C	-11.473954	7.884169	-1.982939
H	-10.950289	7.110744	-0.047881
C	-11.356640	7.737494	-3.370335
H	-10.465602	6.556929	-4.941716
H	-12.064403	8.701046	-1.569047
H	-11.839159	8.440716	-4.047102
C	-10.346092	2.892813	-1.728067
C	-9.796878	2.484188	-2.952796
C	-11.695726	2.582843	-1.488561
C	-10.561603	1.798442	-3.901286
H	-8.752545	2.698116	-3.160336
C	-12.470485	1.915046	-2.437159
H	-12.130373	2.875761	-0.536698
C	-11.903433	1.515238	-3.650644
H	-10.100886	1.479562	-4.834121
H	-13.517230	1.700374	-2.226537
H	-12.500314	0.982094	-4.388838
C	-9.683797	2.727274	3.013688
C	-11.021718	2.707446	3.439663
C	-8.748604	1.990800	3.757960
C	-11.410218	1.992818	4.574282

H	-11.756773	3.270258	2.870491
C	-9.133429	1.264428	4.885597
H	-4.992304	1.244166	3.612832
C	-10.466652	1.265374	5.302992
H	-12.453272	2.001803	4.887549
H	-8.388269	0.688008	5.428554
H	-10.767295	0.701127	6.184177
C	-9.353788	5.749370	3.011561
C	-9.128594	5.369636	4.359373
C	-9.807391	7.080784	2.823693
C	-9.389023	6.230787	5.423911
H	-8.723319	4.391145	4.579292
C	-10.068595	7.938577	3.885827
H	-9.933013	7.436294	1.807051
C	-9.873242	7.522795	5.207634
H	-9.193457	5.885400	6.438754
H	-10.423799	8.947543	3.678049
H	-10.067020	8.195304	6.041533
C	-7.549448	-2.914657	-2.224941
C	-7.193680	-2.518231	-3.523125
C	-8.911820	-3.101392	-1.942038
C	-8.164328	-2.319415	-4.504893
H	-6.146510	-2.359594	-3.761772
C	-9.883405	-2.907884	-2.922240
H	-9.206603	-3.387123	-0.936542
C	-9.513913	-2.514948	-4.210391
H	-7.862572	-2.002687	-5.500740
H	-10.933029	-3.048553	-2.674525
H	-10.272240	-2.349895	-4.972312
C	-5.495956	-4.973338	-2.557932
C	-6.532683	-5.891373	-2.787154
C	-4.458168	-4.896402	-3.498365
C	-6.537808	-6.701661	-3.922655
H	-7.344996	-5.959886	-2.068906
C	-4.462975	-5.702629	-4.636525
H	-3.633819	-4.210181	-3.332462
C	-5.502568	-6.608550	-4.854159
H	-7.353345	-7.405142	-4.078387
H	-3.640320	-5.626384	-5.341641
H	-5.502843	-7.240139	-5.740170
C	-5.703858	-1.843613	2.347224
C	-6.076496	-2.471018	3.544293

C	-5.325608	-0.493351	2.392217
C	-6.057560	-1.776503	4.754339
H	-6.368083	-3.517638	3.525405
C	-5.298205	0.201261	3.600981
H	-5.055200	0.015351	1.472261
C	-5.660058	-0.439349	4.788984
H	-6.345714	-2.285173	5.671915
H	-7.712169	1.978027	3.435682
H	-5.631703	0.101679	5.731943
C	-3.694856	-3.899723	2.010857
C	-2.754502	-2.959318	2.457598
C	-3.732066	-5.150620	2.644431
C	-1.881262	-3.258278	3.502719
H	-2.708568	-1.985057	1.980935
C	-2.866866	-5.449035	3.697131
H	-4.446187	-5.894837	2.302444
C	-1.934939	-4.503981	4.130233
H	-1.155238	-2.515955	3.824464
H	-2.921652	-6.422863	4.178577
H	-1.253598	-4.737106	4.944899
C	0.744080	-5.756932	-3.460070
C	0.922245	-4.393257	-3.744902
C	-0.092452	-6.497387	-4.311406
C	0.301090	-3.797622	-4.842816
H	1.561909	-3.796479	-3.102870
C	-0.706162	-5.909259	-5.417352
H	-0.250671	-7.551385	-4.099002
C	-0.513183	-4.552161	-5.689262
H	0.456110	-2.738755	-5.036617
H	-1.332967	-6.514248	-6.069941
H	-0.991753	-4.088176	-6.549061
C	3.603686	-5.930435	-3.358533
C	3.636227	-6.422267	-4.670946
C	4.308152	-4.750066	-3.075008
C	4.342788	-5.753486	-5.672861
H	3.094500	-7.334810	-4.905960
C	5.014601	-4.079882	-4.072160
H	4.313108	-4.370351	-2.058076
C	5.034373	-4.578462	-5.377499
H	4.350720	-6.152378	-6.685713
H	5.557026	-3.170359	-3.826079
H	5.588357	-4.056423	-6.154803

C	3.155889	-9.273884	0.394103
C	2.970485	-9.440009	1.778517
C	3.906922	-10.258782	-0.277594
C	3.488218	-10.547077	2.453493
H	2.417437	-8.688811	2.332732
C	4.422006	-11.364915	0.391679
H	4.087606	-10.136651	-1.341518
C	4.212916	-11.520231	1.767092
H	3.330022	-10.639602	3.526410
H	4.994230	-12.108190	-0.160800
H	4.623179	-12.379023	2.294856
C	0.279919	-8.710392	0.533539
C	-0.313945	-8.121636	1.659774
C	-0.001655	-10.059254	0.266837
C	-1.150383	-8.859925	2.497937
H	-0.124990	-7.073713	1.873099
C	-0.835755	-10.800997	1.104442
H	0.454165	-10.528464	-0.600655
C	-1.414108	-10.203864	2.226035
H	-1.599360	-8.377886	3.362982
H	-1.033064	-11.847609	0.879649
H	-2.066360	-10.779029	2.880409
C	10.200369	-5.570526	-1.992022
C	10.103875	-5.279428	-3.370217
C	11.018687	-6.666843	-1.636718
C	10.803974	-6.016929	-4.325706
H	9.446618	-4.481560	-3.693807
C	11.720205	-7.402011	-2.587495
H	11.109405	-6.918919	-0.584662
C	11.622809	-7.082010	-3.947192
H	10.694569	-5.761534	-5.378897
H	12.351720	-8.229556	-2.265845
H	12.162375	-7.661170	-4.694736
C	10.425966	-2.580659	-1.611462
C	11.720705	-2.217636	-1.202326
C	9.965729	-2.061896	-2.831243
C	12.530301	-1.395475	-1.985855
H	12.082388	-2.592141	-0.248580
C	10.765170	-1.224923	-3.615369
H	8.959892	-2.304896	-3.161494
C	12.053345	-0.891805	-3.199640
H	13.533597	-1.142352	-1.646126

H	10.372288	-0.826416	-4.548725
H	12.676730	-0.239379	-3.808867
C	9.227771	-6.196484	2.519446
C	8.919306	-6.060072	3.897076
C	9.725774	-7.467626	2.131349
C	9.141832	-7.089381	4.810217
H	8.477836	-5.141028	4.258380
C	9.949170	-8.492913	3.042954
H	9.919069	-7.638021	1.077899
C	9.669760	-8.316485	4.402938
H	8.881278	-6.929005	5.856093
H	10.341546	-9.444623	2.685588
H	9.833747	-9.120832	5.118031
C	9.481854	-3.214246	3.055232
C	10.788696	-3.237330	3.568239
C	8.476430	-2.638446	3.848194
C	11.080011	-2.717659	4.830986
H	11.577458	-3.677551	2.963967
C	8.764233	-2.106183	5.105520
H	7.462648	-2.595330	3.462508
C	10.067976	-2.146975	5.605796
H	12.100833	-2.755857	5.208672
H	7.967288	-1.648368	5.686485
H	10.293021	-1.734499	6.587855
C	7.662099	3.235413	-1.293178
C	7.383748	3.103859	-2.662386
C	9.007149	3.315623	-0.898581
C	8.411958	3.055356	-3.603690
H	6.350976	3.032923	-2.989413
C	10.036290	3.270443	-1.837114
H	9.242674	3.396849	0.158350
C	9.743521	3.139420	-3.196329
H	8.168838	2.942502	-4.657853
H	11.070376	3.321013	-1.504158
H	10.546540	3.089210	-3.928070
C	5.642532	5.357543	-1.385717
C	6.701938	6.278598	-1.382982
C	4.663130	5.477189	-2.382896
C	6.787261	7.280287	-2.349973
H	7.468825	6.198643	-0.617540
C	4.747688	6.476258	-3.352626
H	3.821432	4.792167	-2.393341

C	5.810150	7.381866	-3.341419
H	7.619284	7.981339	-2.327057
H	3.968396	6.548362	-4.106023
H	5.872599	8.163201	-4.096104
C	5.501495	1.397320	2.881378
C	5.781845	1.781636	4.200084
C	5.116722	0.069413	2.641854
C	5.664498	0.872583	5.252322
H	6.078504	2.807302	4.402018
C	4.991671	-0.839316	3.691813
H	4.918596	-0.252282	1.624277
C	5.260669	-0.439756	5.003530
H	5.881643	1.194315	6.268709
H	4.683128	-1.860440	3.483257
H	5.155996	-1.147560	5.822526
C	3.536901	3.522443	2.791983
C	3.539746	4.640088	3.639450
C	2.560059	2.537130	2.999380
C	2.605386	4.764759	4.667835
H	4.282199	5.418362	3.484760
C	1.617473	2.664372	4.019017
H	2.540152	1.663684	2.354969
C	1.636997	3.777780	4.860660
H	2.634587	5.636069	5.318337
H	0.864497	1.891942	4.153358
H	0.901646	3.876966	5.655433

Structure 3b: Atom numer: 362

Total energy (Hartree): -8318.89599044

C	2.955815	5.292128	0.969356
C	1.753982	5.991900	0.850373
C	0.554406	5.336066	0.523090
C	0.622416	3.942702	0.328005
C	1.814887	3.244777	0.454642
C	3.015278	3.901231	0.778509
H	3.863928	5.838056	1.210593
H	1.748385	7.065039	1.017868
H	-0.285839	3.399170	0.087025
H	1.819635	2.169500	0.301846
C	-0.786386	5.987086	0.401737
C	-1.524300	5.825617	-0.801850
C	-1.412272	6.622977	1.501648

C	-2.904439	6.107659	-0.841458
C	-2.789202	6.930198	1.455532
C	-3.573870	6.580682	0.317204
C	-5.055637	6.534770	0.402639
C	-5.948150	7.098065	-0.532671
C	-5.644253	5.729752	1.404212
C	-7.301929	6.781565	-0.547430
H	-5.569438	7.786404	-1.284196
C	-6.982555	5.388622	1.372835
H	-5.010405	5.286769	2.167755
C	-7.863724	5.822694	0.340013
H	-7.927966	7.259885	-1.289181
H	-7.347470	4.674082	2.101119
C	-9.135064	5.110610	0.193307
C	-9.697264	4.702423	-1.069971
C	-9.740743	4.498892	1.368350
C	-10.305217	3.447885	-1.161877
C	-10.304593	3.230528	1.278557
C	-10.278819	2.557774	-0.005759
C	-9.875725	1.164194	-0.165203
C	-9.926662	0.165948	0.851120
C	-9.141520	0.777158	-1.327469
C	-9.113893	-0.957669	0.821514
H	-10.562577	0.307152	1.714021
C	-8.360548	-0.363473	-1.366683
H	-9.090866	1.455754	-2.169677
C	-8.239932	-1.222928	-0.252339
H	-9.125768	-1.629625	1.676875
H	-7.742473	-0.541762	-2.243432
C	-7.093519	-2.165243	-0.177709
C	-6.099487	-1.969828	0.817846
C	-6.881131	-3.167443	-1.163371
C	-4.882073	-2.676826	0.760336
C	-5.658268	-3.873503	-1.221451
C	-4.625086	-3.590781	-0.294906
C	4.271987	3.093771	0.874519
C	5.341439	3.304476	-0.029332
C	4.360825	2.036523	1.818068
C	6.432645	2.408624	-0.059800
C	5.467101	1.159617	1.805580
C	6.482067	1.313996	0.835336
C	7.564028	0.285924	0.702725

C	8.624864	0.172268	1.613268
C	7.531143	-0.619370	-0.367448
C	9.616578	-0.792817	1.446558
H	8.679689	0.853780	2.458353
C	8.504058	-1.605772	-0.515881
H	6.724417	-0.554132	-1.093156
C	9.571879	-1.712568	0.385297
H	10.437286	-0.831922	2.157076
H	8.435036	-2.295637	-1.350118
C	-3.237712	-4.122935	-0.471188
C	-2.193431	-3.202246	-0.679470
C	-2.890892	-5.484663	-0.446881
C	-0.878055	-3.615414	-0.835041
H	-2.423285	-2.142048	-0.718804
C	-1.567360	-5.901243	-0.597921
H	-3.666991	-6.231099	-0.303077
C	-0.525952	-4.976621	-0.788854
H	-0.102347	-2.870032	-0.983303
H	-1.339330	-6.962489	-0.554423
C	0.922277	-5.335872	-0.896986
C	1.598866	-5.968194	0.174613
C	1.667045	-4.926998	-2.036464
C	3.006630	-6.067167	0.167094
C	3.073684	-5.041457	-2.049478
C	3.754992	-5.562025	-0.925795
C	5.249551	-5.527013	-0.853823
C	5.881277	-4.686096	0.076490
C	6.074345	-6.304710	-1.682184
C	7.268647	-4.619660	0.176914
H	5.270322	-4.072113	0.732609
C	7.462292	-6.255177	-1.567474
H	5.622680	-6.964023	-2.418475
C	8.091580	-5.407818	-0.640467
H	7.713549	-3.955633	0.909748
H	8.068796	-6.885983	-2.211762
C	10.691614	-2.765526	0.217239
C	10.833610	-3.280365	-1.213259
C	10.575582	-3.895474	1.244925
C	10.280065	-4.471848	-1.565506
C	10.125522	-5.123901	0.893016
C	9.631101	-5.387829	-0.525454
C	-3.659570	5.807774	-2.104643

C	-4.470151	4.666862	-2.200435
C	-3.520982	6.624450	-3.235199
C	-5.102640	4.338623	-3.398918
H	-4.605598	4.036579	-1.326510
C	-4.159854	6.303182	-4.434301
H	-2.894124	7.510110	-3.175951
C	-4.946898	5.154600	-4.523173
H	-5.715715	3.442510	-3.454164
H	-4.033971	6.947634	-5.301456
H	-5.435092	4.894439	-5.459428
C	-0.811566	5.386654	-2.047937
C	0.120613	6.248970	-2.645211
C	-1.061651	4.153073	-2.669549
C	0.761071	5.904479	-3.835517
H	0.332003	7.204477	-2.172742
C	-0.412503	3.799369	-3.852071
H	-1.779966	3.470403	-2.226698
C	0.497999	4.675627	-4.445305
H	1.460329	6.602273	-4.290547
H	-0.624486	2.837840	-4.313246
H	0.995138	4.404667	-5.373755
C	-3.448202	7.651309	2.591862
C	-3.477769	7.136061	3.897238
C	-4.056819	8.896899	2.362778
C	-4.085571	7.841800	4.935897
H	-3.024651	6.170351	4.098322
C	-4.660691	9.606040	3.399081
H	-4.058136	9.303874	1.355557
C	-4.677650	9.081417	4.693802
H	-4.100676	7.415687	5.936386
H	-5.125648	10.567547	3.193500
H	-5.156141	9.629150	5.502193
C	-0.603608	6.983130	2.713705
C	-0.118146	6.006185	3.594974
C	-0.311633	8.328337	2.985849
C	0.625030	6.365560	4.719544
H	-0.318152	4.957928	3.393496
C	0.434551	8.689228	4.108273
H	-0.685659	9.095673	2.313779
C	0.904717	7.707933	4.982080
H	0.987240	5.591130	5.390787
H	0.645458	9.738868	4.300351

H	1.484855	7.986055	5.858974
C	-9.707391	5.660198	-2.227708
C	-8.683840	5.692223	-3.186872
C	-10.759416	6.579219	-2.358140
C	-8.709223	6.607396	-4.239639
H	-7.843643	5.012616	-3.084806
C	-10.796626	7.488970	-3.416966
H	-11.557624	6.569752	-1.620534
C	-9.769314	7.508066	-4.362332
H	-7.891412	6.619687	-4.956373
H	-11.628650	8.185278	-3.500967
H	-9.792545	8.220736	-5.184029
C	-11.102548	3.002950	-2.345472
C	-10.673616	3.088704	-3.685356
C	-12.376204	2.436512	-2.123565
C	-11.480514	2.658990	-4.739088
H	-9.689040	3.482333	-3.906471
C	-13.183981	2.007657	-3.173368
H	-12.723036	2.336602	-1.099512
C	-12.743619	2.119021	-4.495239
H	-11.109310	2.738189	-5.759097
H	-14.163295	1.584274	-2.957797
H	-13.369247	1.780429	-5.318294
C	-11.058807	2.624464	2.426877
C	-12.463265	2.619133	2.387318
C	-10.434761	2.039475	3.541004
C	-13.216910	2.076875	3.428713
H	-12.961985	3.059890	1.528212
C	-11.184617	1.486408	4.580514
H	-6.284891	2.456712	2.287952
C	-12.579314	1.507753	4.533653
H	-14.303648	2.098589	3.376567
H	-10.673667	1.030930	5.426085
H	-13.163383	1.079009	5.345215
C	-9.897314	5.381815	2.571447
C	-9.434191	5.066110	3.860457
C	-10.540402	6.624667	2.408598
C	-9.626654	5.934236	4.936672
H	-8.899349	4.137385	4.020414
C	-10.737335	7.493464	3.479636
H	-10.886777	6.899510	1.416413
C	-10.283156	7.151096	4.756369

H	-9.248668	5.658860	5.919151
H	-11.246542	8.441469	3.316841
H	-10.429022	7.828755	5.594850
C	-7.979852	-3.463272	-2.138464
C	-7.807896	-3.305395	-3.522343
C	-9.227095	-3.914875	-1.676457
C	-8.841401	-3.596726	-4.413429
H	-6.855538	-2.948019	-3.901797
C	-10.259286	-4.211569	-2.564399
H	-9.384216	-4.024948	-0.607410
C	-10.071059	-4.053973	-3.939883
H	-8.684019	-3.460012	-5.480857
H	-11.215737	-4.559046	-2.180565
H	-10.878210	-4.277337	-4.633602
C	-5.453526	-4.931575	-2.265409
C	-6.191355	-6.124331	-2.215825
C	-4.529931	-4.765810	-3.308109
C	-6.019299	-7.116815	-3.181208
H	-6.912961	-6.265716	-1.416155
C	-4.359516	-5.754836	-4.276637
H	-3.936264	-3.857869	-3.355861
C	-5.103157	-6.935049	-4.218149
H	-6.604435	-8.031877	-3.122856
H	-3.639551	-5.599867	-5.075963
H	-4.968448	-7.706418	-4.973001
C	-6.280993	-0.931181	1.888100
C	-6.458978	-1.296079	3.229635
C	-6.226575	0.433796	1.569804
C	-6.566732	-0.325555	4.226939
H	-6.502352	-2.349491	3.492683
C	-6.331232	1.406397	2.562712
H	-6.116309	0.733907	0.532187
C	-6.496861	1.029096	3.898374
H	-6.699072	-0.630399	5.262759
H	-9.351435	1.975023	3.565834
H	-6.569434	1.786568	4.675425
C	-3.857630	-2.465779	1.838443
C	-3.152763	-1.258828	1.964232
C	-3.618588	-3.473186	2.784551
C	-2.246841	-1.059186	3.005747
H	-3.329989	-0.463163	1.247090
C	-2.725465	-3.271057	3.837321

H	-4.151996	-4.416126	2.698901
C	-2.033997	-2.062992	3.952337
H	-1.713790	-0.114780	3.081687
H	-2.582997	-4.053207	4.579327
H	-1.342401	-1.903433	4.776365
C	0.949667	-4.402572	-3.243656
C	1.131889	-3.087896	-3.700692
C	0.082504	-5.239409	-3.962975
C	0.474347	-2.626873	-4.841114
H	1.796225	-2.422690	-3.157291
C	-0.567484	-4.784081	-5.109462
H	-0.080492	-6.256153	-3.616674
C	-0.375470	-3.473700	-5.553884
H	0.627328	-1.602532	-5.171665
H	-1.223085	-5.456405	-5.657553
H	-0.886056	-3.115729	-6.444421
C	3.855613	-4.602841	-3.253142
C	3.783476	-5.327326	-4.451698
C	4.677509	-3.467224	-3.210029
C	4.508164	-4.927614	-5.575149
H	3.146941	-6.206273	-4.502378
C	5.401509	-3.064580	-4.332225
H	4.753216	-2.900217	-2.286724
C	5.319124	-3.793591	-5.520100
H	4.436672	-5.503757	-6.494687
H	6.030571	-2.179543	-4.277157
H	5.884011	-3.480516	-6.394725
C	3.722135	-6.702718	1.321341
C	3.692973	-6.126309	2.601562
C	4.439396	-7.896382	1.149640
C	4.350854	-6.727836	3.674896
H	3.144331	-5.201962	2.755467
C	5.094018	-8.502455	2.222064
H	4.479991	-8.352360	0.164494
C	5.052484	-7.920137	3.491426
H	4.311767	-6.263102	4.657098
H	5.633630	-9.433837	2.065701
H	5.564384	-8.389687	4.327703
C	0.829925	-6.556107	1.321163
C	0.166823	-5.754058	2.259825
C	0.771613	-7.949948	1.473723
C	-0.520249	-6.330507	3.328198

H	0.178320	-4.673906	2.148777
C	0.079010	-8.527798	2.538060
H	1.280560	-8.582798	0.751848
C	-0.567136	-7.718041	3.472671
H	-1.024708	-5.688034	4.043226
H	0.047211	-9.610472	2.636462
H	-1.106430	-8.163871	4.305114
C	10.246063	-4.977359	-2.966995
C	9.723471	-4.189620	-4.009208
C	10.708126	-6.267435	-3.294933
C	9.667239	-4.665530	-5.316823
H	9.353029	-3.195237	-3.780844
C	10.659964	-6.742441	-4.605223
H	11.130045	-6.899977	-2.517741
C	10.136596	-5.945377	-5.625330
H	9.244622	-4.037307	-6.097073
H	11.033637	-7.739349	-4.828812
H	10.090065	-6.317694	-6.645662
C	11.677655	-2.449125	-2.113764
C	12.680602	-3.042810	-2.910885
C	11.583935	-1.040765	-2.139814
C	13.529566	-2.275572	-3.702512
H	12.796337	-4.121503	-2.894610
C	12.432848	-0.274317	-2.937521
H	10.826280	-0.539393	-1.548002
C	13.411127	-0.881872	-3.726639
H	14.293753	-2.768821	-4.299606
H	12.316316	0.806970	-2.943802
H	14.071949	-0.282162	-4.348024
C	10.175293	-6.315121	1.789085
C	9.072134	-7.175907	1.956355
C	11.378867	-6.665377	2.433847
C	9.169886	-8.323519	2.743484
H	8.125547	-6.937648	1.484388
C	11.475009	-7.812834	3.216577
H	12.247615	-6.026805	2.307768
C	10.368347	-8.651775	3.379108
H	8.294514	-8.957314	2.863514
H	12.420112	-8.055628	3.697616
H	10.441068	-9.547306	3.991733
C	10.985501	-3.550344	2.637047
C	12.250584	-2.995166	2.908238

C	10.108494	-3.741567	3.719191
C	12.626768	-2.656505	4.207937
H	12.954314	-2.842201	2.093590
C	10.479972	-3.396266	5.017041
H	9.122858	-4.155147	3.531020
C	11.742140	-2.852954	5.270262
H	13.614158	-2.237953	4.389932
H	9.775745	-3.542873	5.832180
H	12.030517	-2.581076	6.282684
C	7.543833	2.598758	-1.049271
C	7.320939	2.462347	-2.428099
C	8.840944	2.912839	-0.616036
C	8.358454	2.639371	-3.343302
H	6.323917	2.219185	-2.783198
C	9.879535	3.095395	-1.528955
H	9.033150	3.014950	0.448140
C	9.642214	2.959260	-2.898638
H	8.162032	2.526746	-4.406709
H	10.874966	3.344732	-1.168892
H	10.449804	3.101335	-3.612639
C	5.325364	4.473113	-0.969337
C	6.261760	5.507279	-0.818568
C	4.399080	4.560621	-2.017952
C	6.278885	6.592909	-1.694479
H	6.985347	5.453420	-0.009949
C	4.417956	5.642520	-2.897891
H	3.651393	3.782693	-2.139251
C	5.358248	6.662524	-2.740719
H	7.013435	7.383376	-1.558459
H	3.686400	5.687315	-3.699009
H	5.369645	7.507233	-3.425536
C	5.579516	0.067866	2.830176
C	5.830059	0.387448	4.172733
C	5.450568	-1.284479	2.481081
C	5.947027	-0.613240	5.137885
H	5.926535	1.430872	4.459605
C	5.564408	-2.287622	3.444221
H	5.263716	-1.551339	1.445099
C	5.813899	-1.954501	4.777112
H	6.140855	-0.342509	6.173130
H	5.456489	-3.330150	3.154646
H	5.902771	-2.735708	5.528064

C	3.278008	1.843856	2.837248
C	3.050886	2.806393	3.832074
C	2.478685	0.690394	2.834153
C	2.066973	2.613848	4.801771
H	3.650448	3.712316	3.842122
C	1.485807	0.501003	3.795491
H	2.637321	-0.064722	2.069936
C	1.278450	1.460992	4.787099
H	1.918262	3.365381	5.573284
H	0.874825	-0.397646	3.765430
H	0.507674	1.314445	5.539516
H	9.946876	-6.409503	-0.774127
H	11.622196	-2.231474	0.449349

6. NMR Spectra

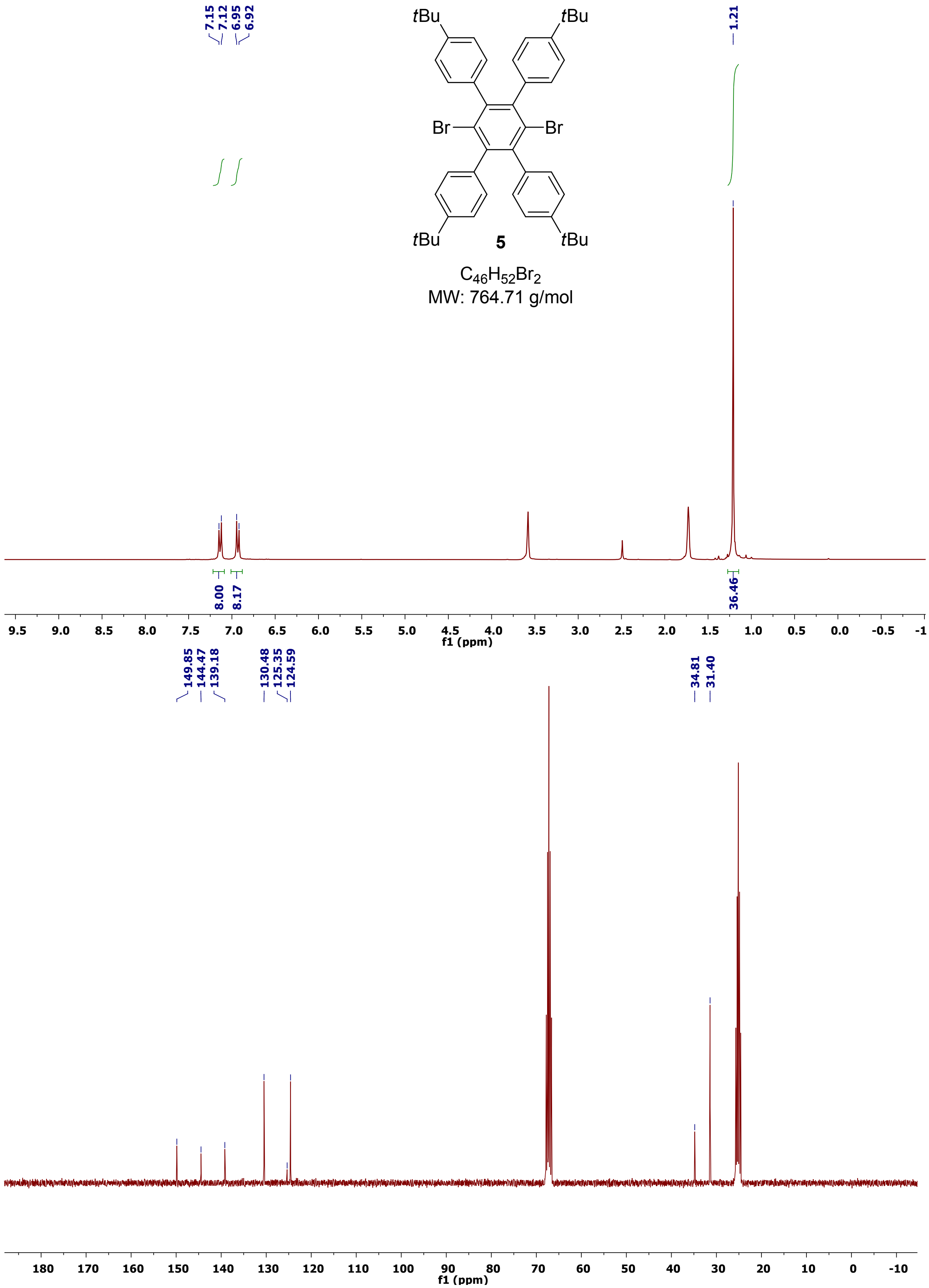

Figure S17: ^{1}H- (300 MHz) and ^{13}C-NMR (75 MHz) spectra of **5** recorded at 298 K in THF-d_8.

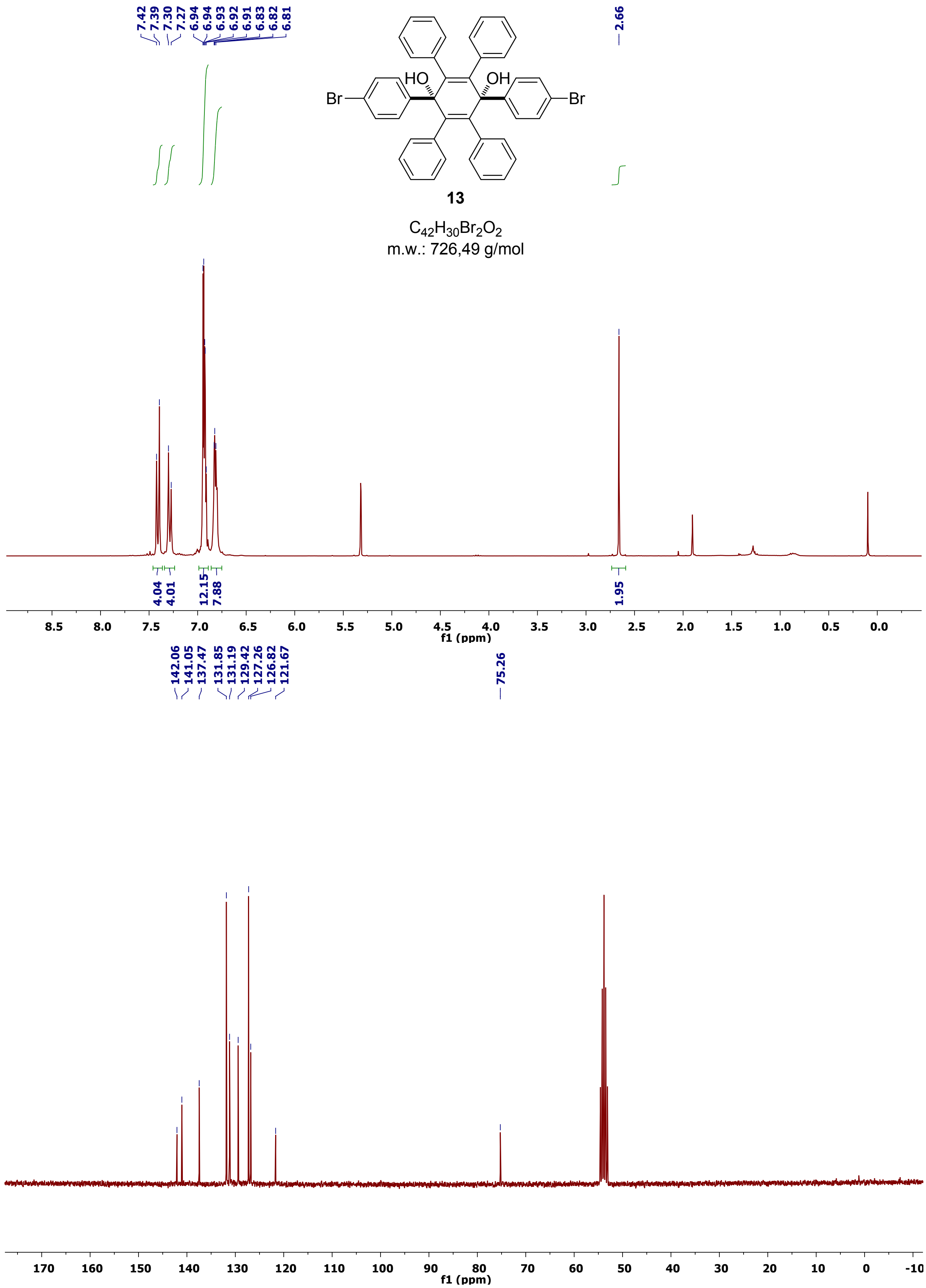

Figure S18: ^{1}H-(300 MHz) and ^{13}C-NMR (75 MHz) spectra of **13** recorded at 298 K in CD_2Cl_2.

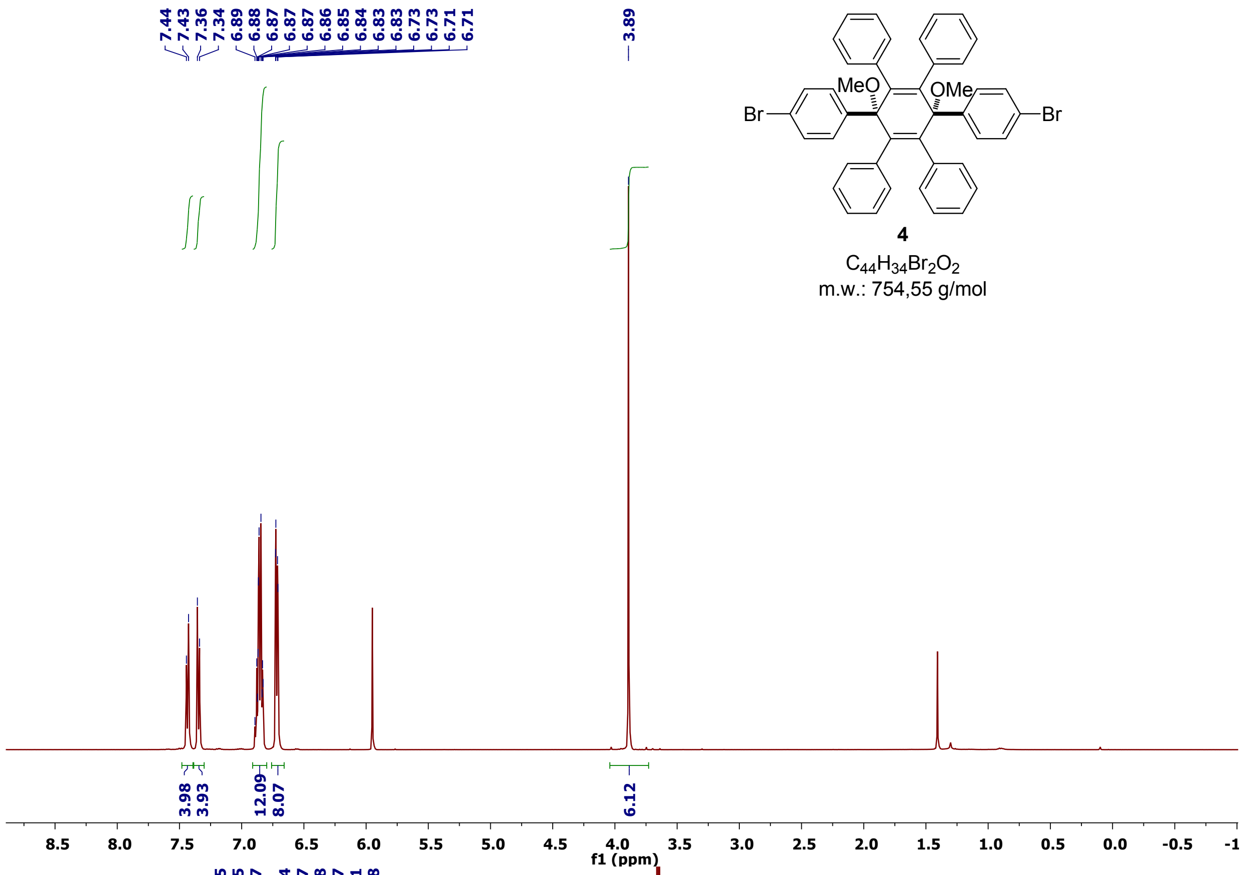

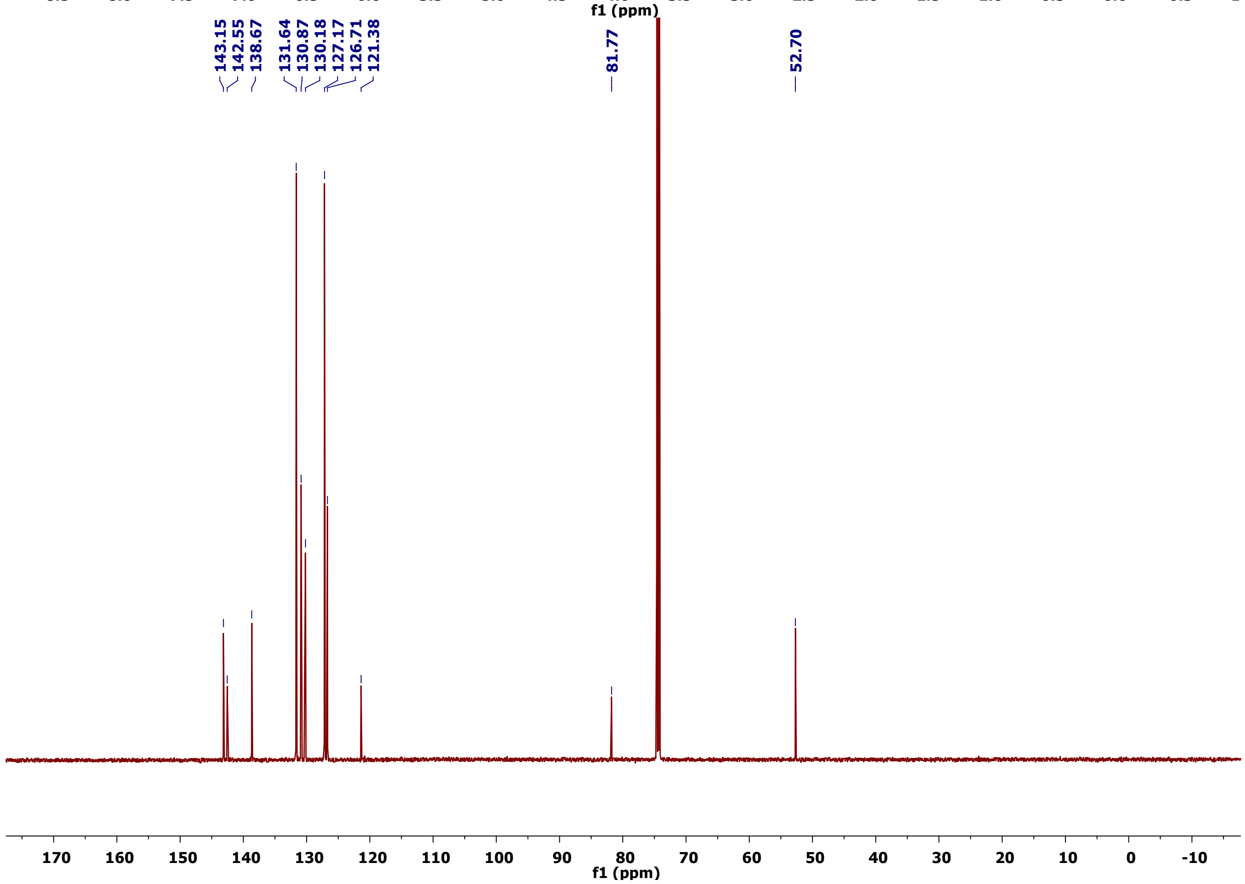

Figure S19: ^{1}H-(500 MHz) and ^{13}C-NMR (126 MHz) spectra of **4** recorded at 373 K in $C_2D_4Cl_2$.

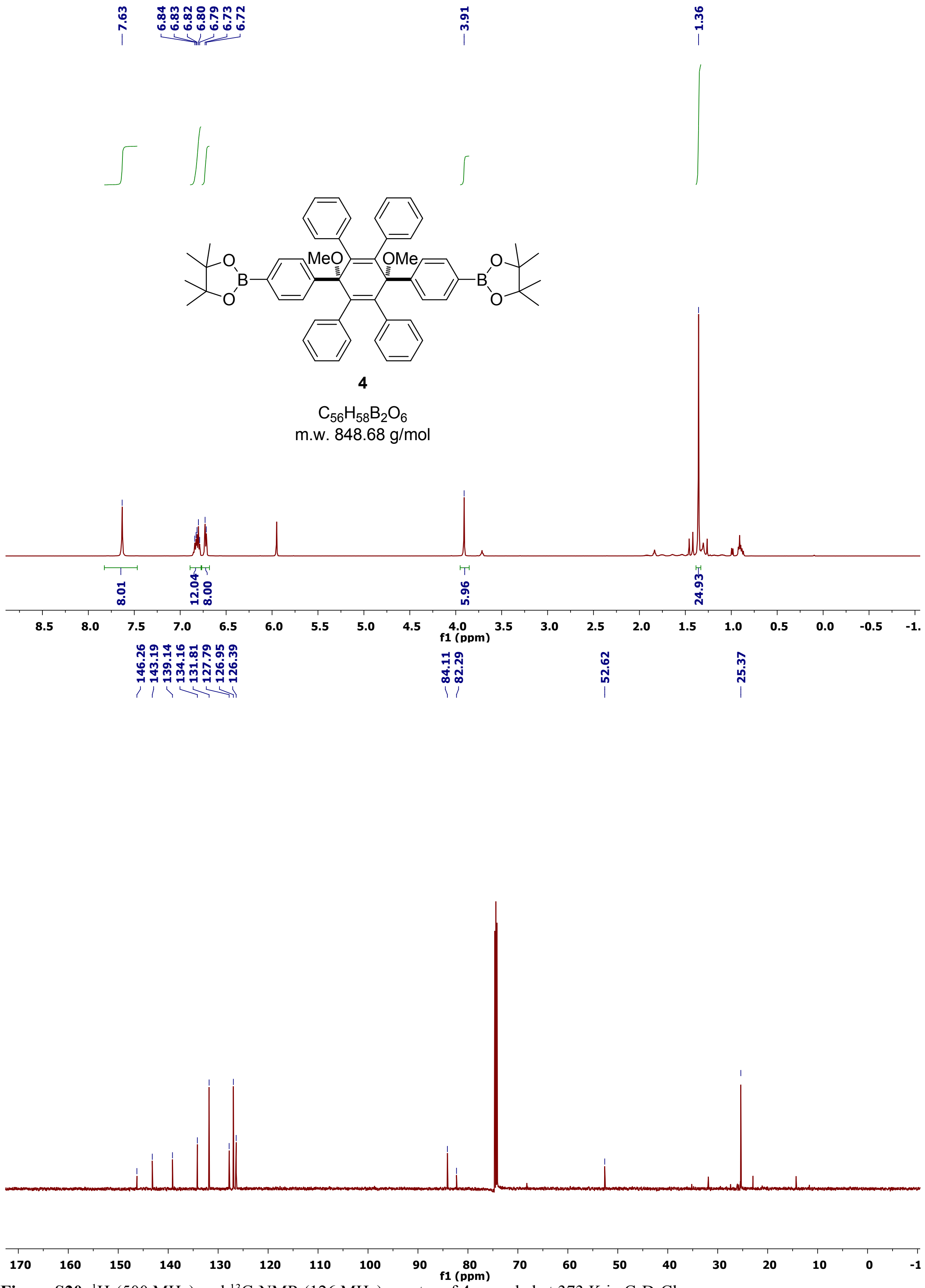

Figure S20: ^{1}H-(500 MHz) and ^{13}C-NMR (126 MHz) spectra of **4** recorded at 373 K in $C_2D_4Cl_2$.

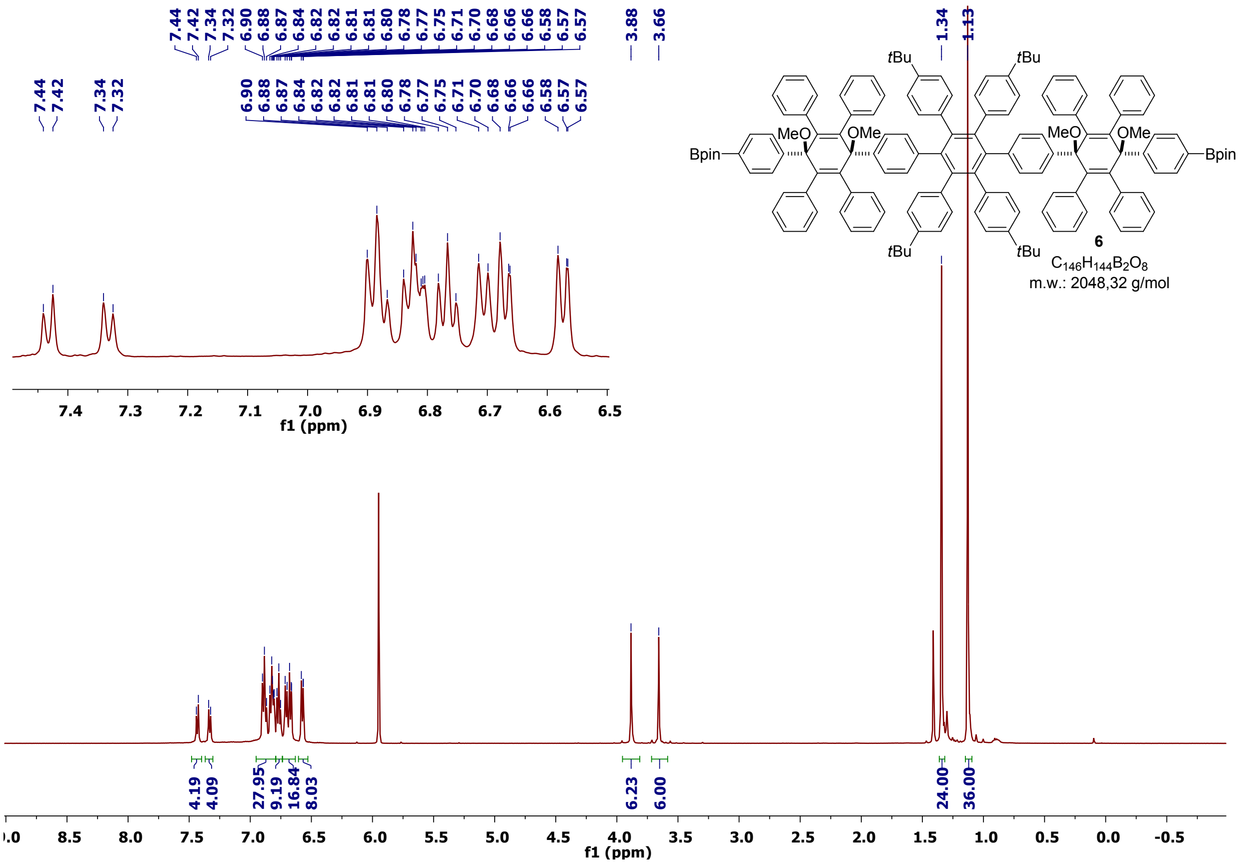

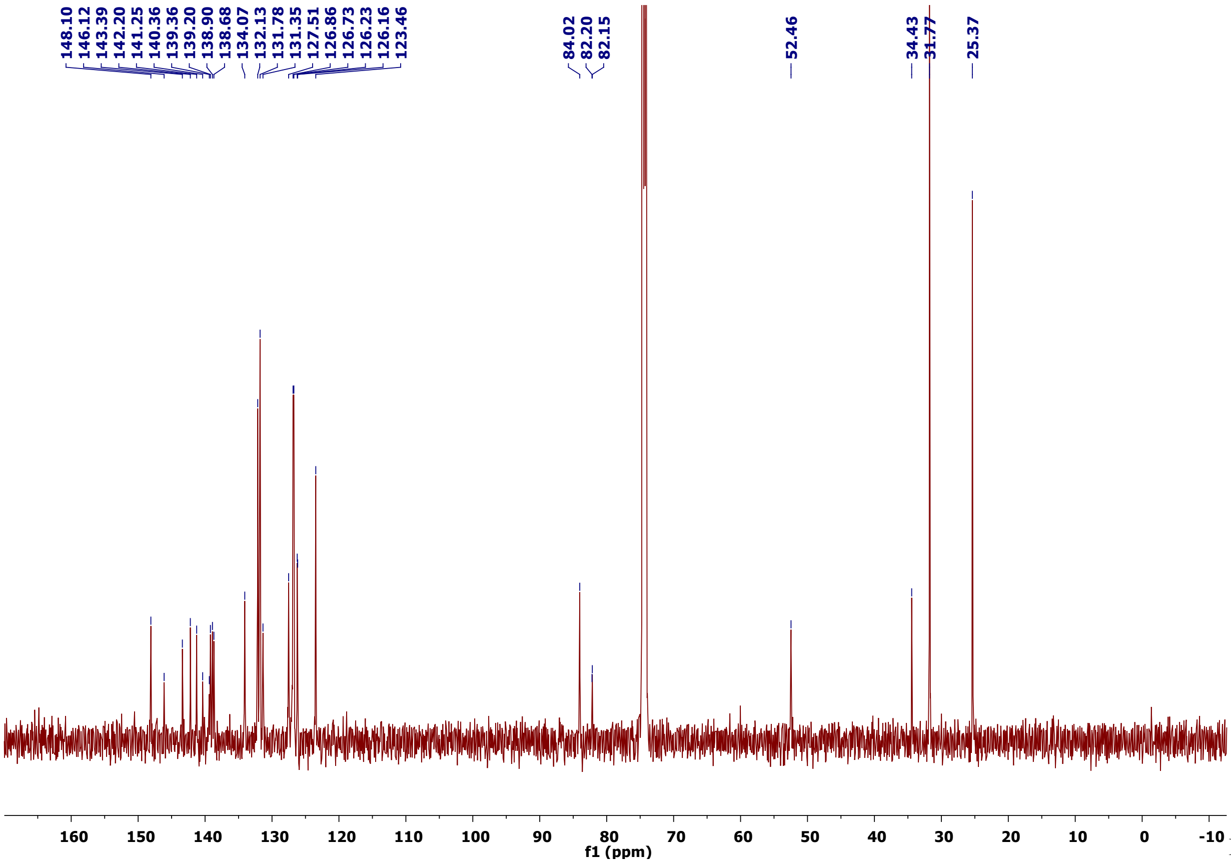

Figure S21: ^{1}H-(500 MHz) and ^{13}C-NMR (126 MHz) spectra of **6** recorded at 373 K in $C_2D_4Cl_2$.

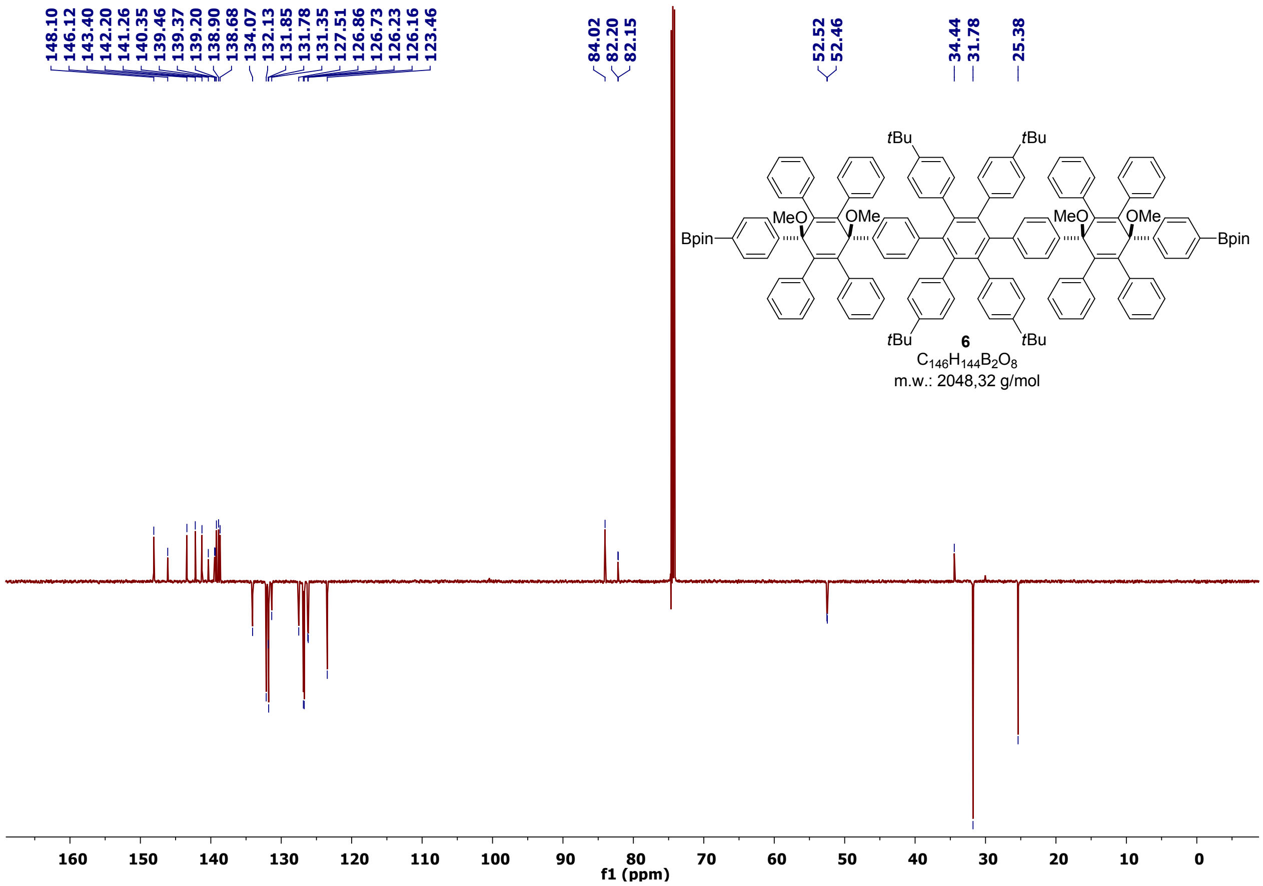

Figure S22: APT-^{13}C-NMR (126 MHz) spectra of **6** recorded at 366 K in $C_2D_4Cl_2$.

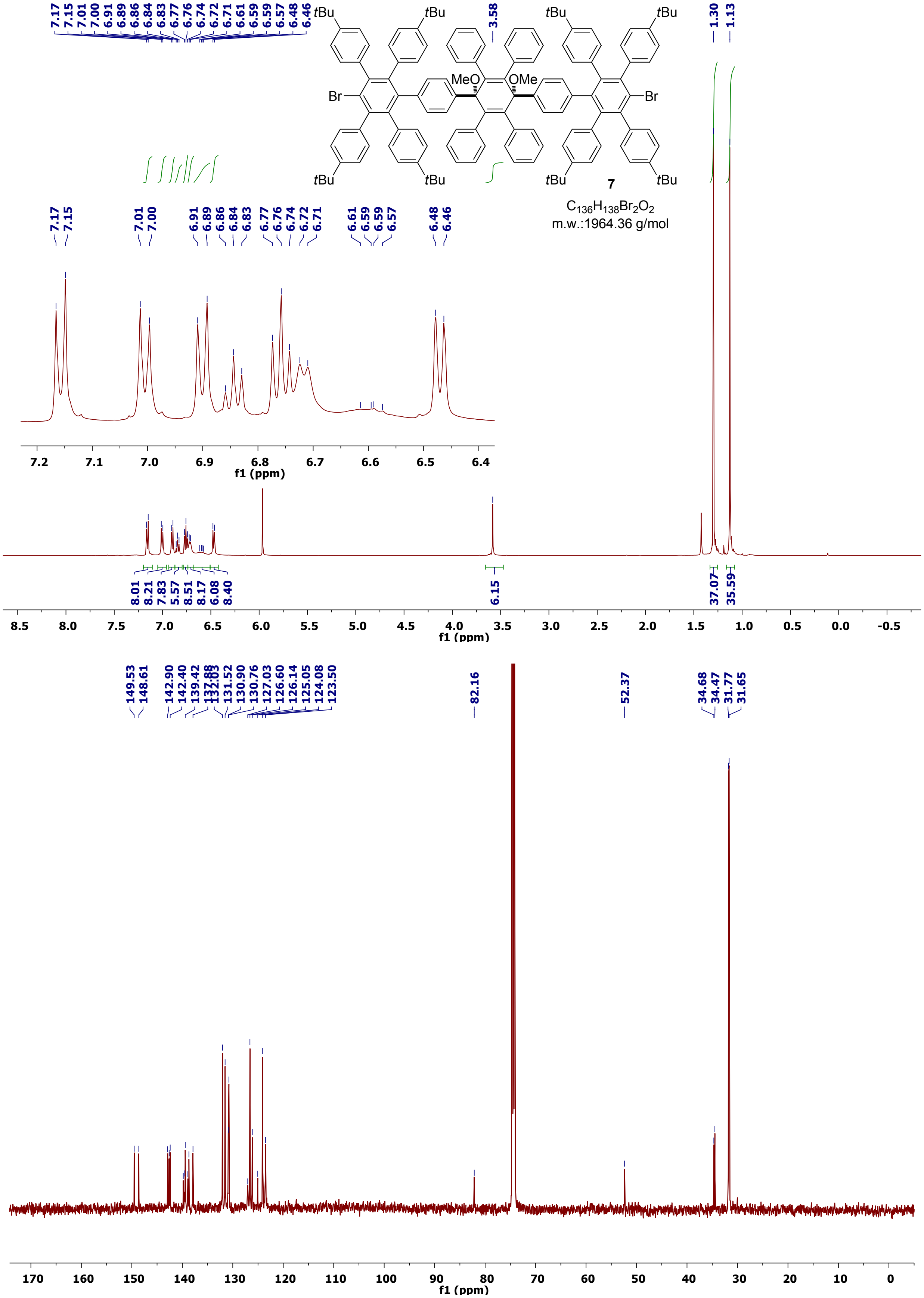

Figure S23: ^{1}H-(500 MHz) and ^{13}C-NMR (126 MHz) spectra of **7** recorded at 373 K in $C_2D_4Cl_2$.

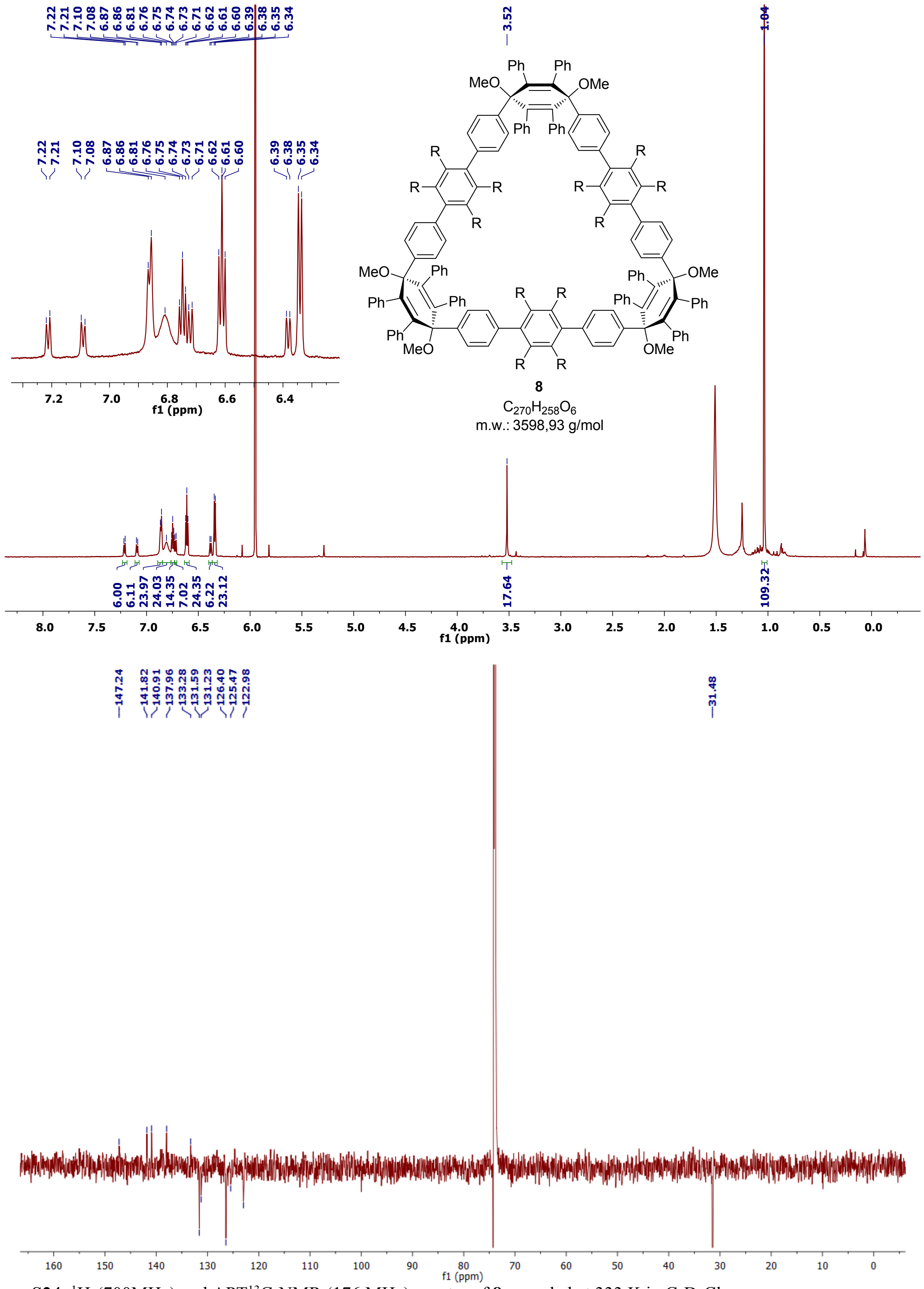

Figure S24: ^{1}H-(700MHz) and APT^{13}C-NMR (176 MHz) spectra of **8** recorded at 333 K in $C_2D_4Cl_2$.

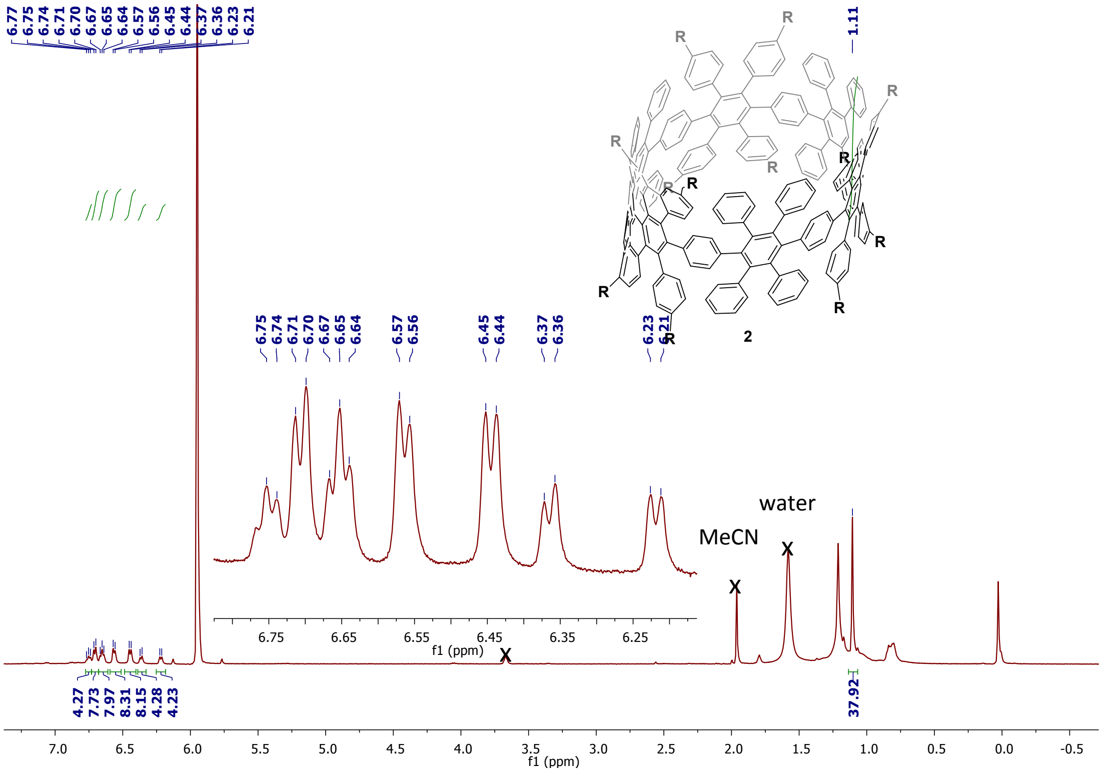

Figure S25: ^{1}**H-NMR** spectrum of compound **2** recorded at r.t. in $C_2D_2Cl_4$**.** Despite prolonged drying at elevated temperatures, MeCN is was still present within the cavities of the crystals. Also aliphatic peaks detected at 0.8 ppm and 1.2. ppm that could not be removed.

For ^{13}*C-NMR* of compound **2**: see p. 17 and p.18 (HSQC and HMBC).

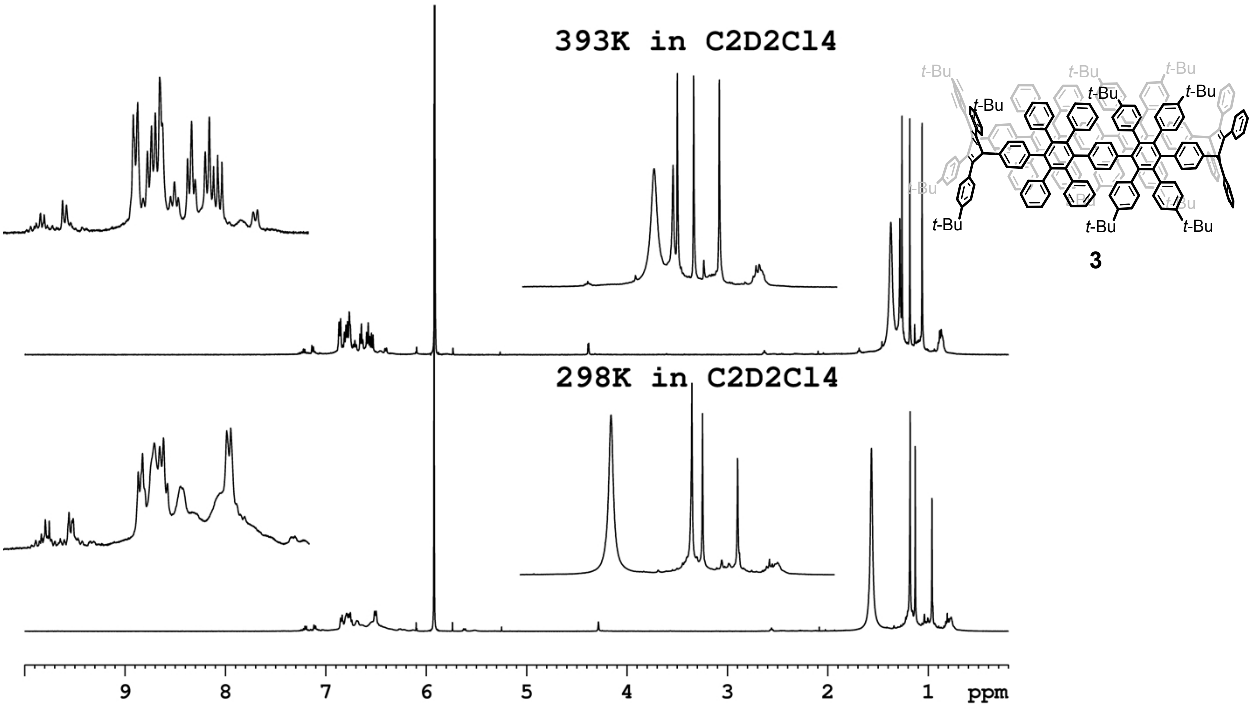

Figure S26: ^{1}*H-NMR* sepctrum of compound **3** recorded at 500 MHz at r.t. and 100 °C. Due to the high unsymmetry of the molecule, only a fingerprint spectrum can be resolved. For structural analysis, see crystal structure on p. S14.

7. MALDI-MS spectra

*7.1 Spectrum of **2** after oxidative cyclodehyrogenation*

Oxidative cyclodehydrogenation of **2** for 1 d yielded a product mixture. Thus, the crude product was separated by preparative TLC to investigate the product distribution, in detail.

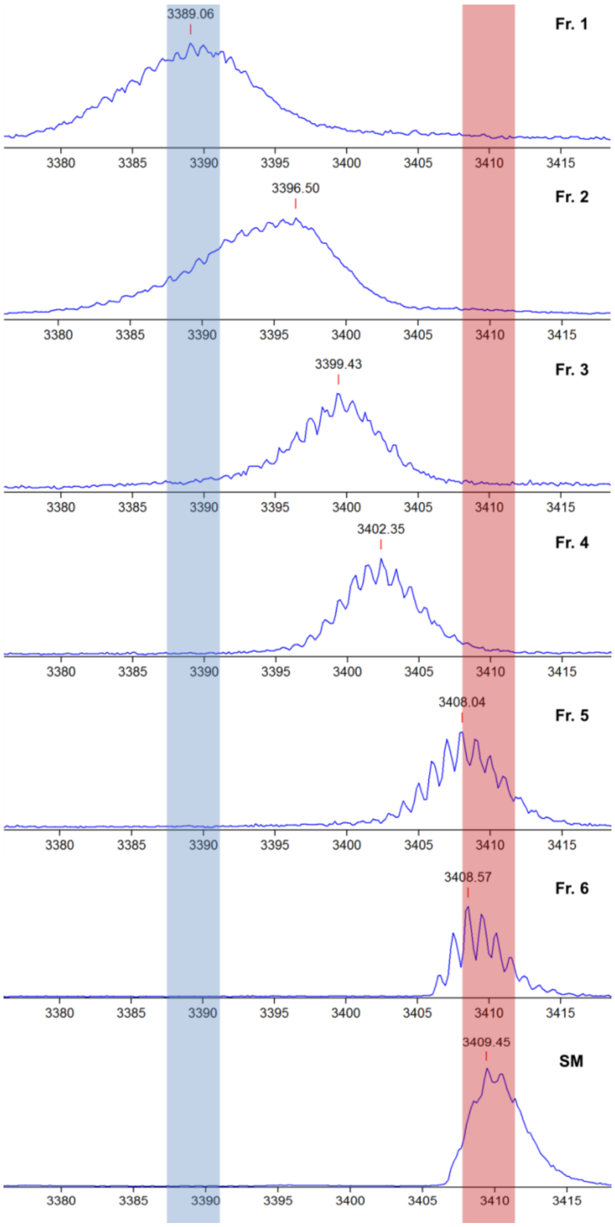

Figure S26: oxidative cyclodehydrogenation of **2**. Upper left (Fr.1) first fraction; bottom: starting material **2**.

In *Figure S26*, the mass spectrum of compound **2** after dehydrogenation is shown. The separation afforded various products ranging from 3389 m/z, as the most unpolar product, to 3408 m/z, as the most polar product. The lightest fraction was subjected to $FeCl_3$ again. No further decrease in mass could be observed. Room temperature and high temperature approaches for cyclodehydrogenation with other oxidizing agents, such as DDQ, $Sc(OTf)_3$ and/or TfOH, led to

decomposition of the starting material and gave product mixtures that did not yield masses in the expected product range.

7.2 C_2-symmetric Congener

The C_2-symmetric compound **3** was oxidatively cyclodehydrogenated with $FeCl_3$. As can be seen from the crystal structure, the two pentaphenylenes that are bridged by two cyclohexa-2,5-dienes are only slightly bent. Thus, compound **3** is expected to undergo oxidative cyclodehydrogenation. In addition, cyclohexadienes are known to form phenylenes in the presence of oxidizing agents.[4]

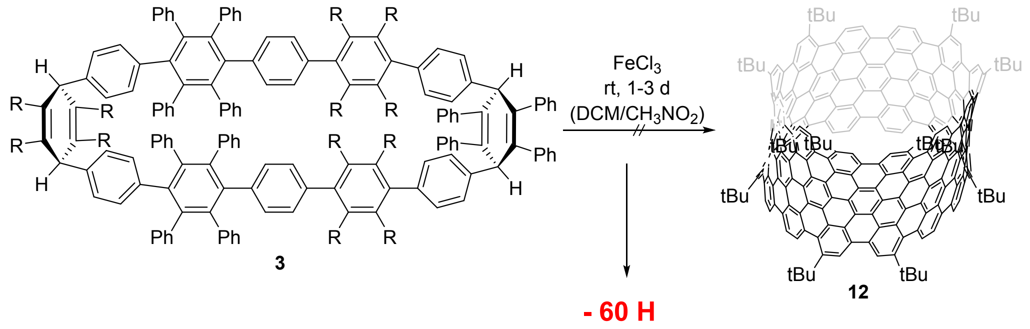

Scheme S3: Oxidative cyclodehydrogenation of **108** with $FeCl_3$

The C_2-symmetric compound **3** was subjected to $FeCl_3$. The reaction progress was monitored by mass spectrometry. Spectra were recorded after 1 d and 3 d. In *Figure S27*, the mass spectrum of **3** after cyclodehydrogenation for 1 d is shown. Four peaks are observed at 3370 m/z, 3355 m/z, 3317 m/z and 3299 m/z. The mass of the starting material is 3415 m/z.

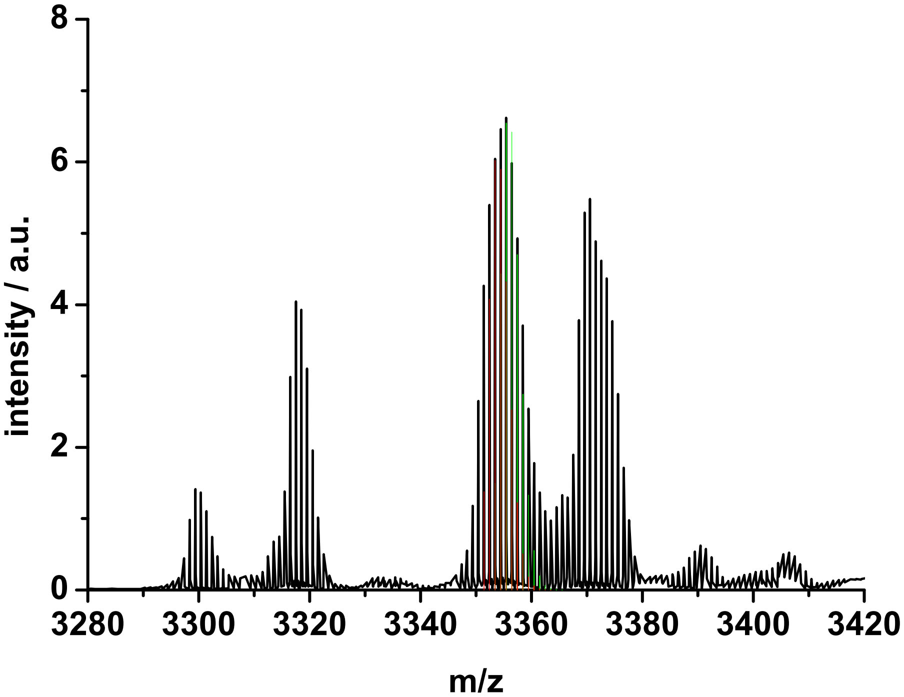

Figure S27: HR-MS (MALDI) spectrum of compound **3** after cyclohydrogenation (1 d). Red isotopic pattern for a cyclodehydrogenated compound with a sum formula of $C_{264}H_{182}$ and its two hydrogen atoms heavier congener: $C_{264}H_{184}$. Reaction conditions: $FeCl_3$ rt, 24 h, filtration trhough a short bad of silica with DCM/THF and subsequent separation by preparative GPC column.

To understand the observed masses, first, a closer look is taken at the intermediate mass of 3355 m/z. The lower masses will be discussed in the following spectra (*vide infra*).

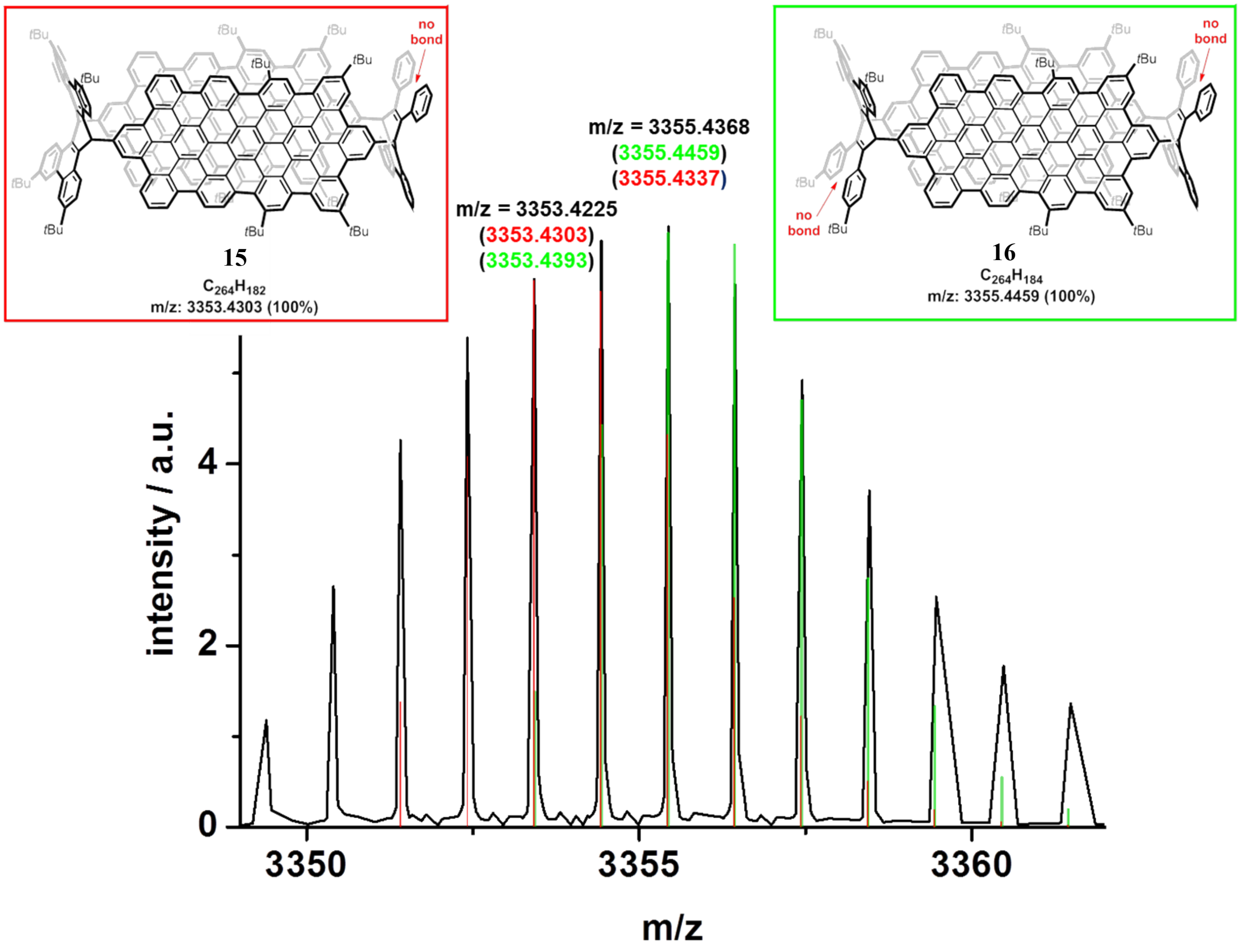

Figure S28: section of the mass spectrum of compound **3** after cyclodehydrogenation for 1 d with $FeCl_3$ at r.t..

In *Figure S28*, a small section of the mass spectrum between 3350 m/z and 3360 m/z is shown. The sum formulas for two interemediates (red, left, **13**; green, right, **11**) are shown. These structures can be proposed based on the structure of the starting material, as the crystal structure hinted at a slightly bent pentaphenylene that should smoothly undergo cyclodehydrogenation to give a short nanoribbon segment. The sum formula of **11** the theoretical mass (green) deviates by 0.01 g/mol; such an error range has been observed for molecules with similar molecular weight, of which the structure had been confirmed by X-ray crystallography before MS measurements. Therefore, the experimental findings may very well be in agreement with the outlined sum formula. One can conclude that the above given sum formula matches with the experimental findings.

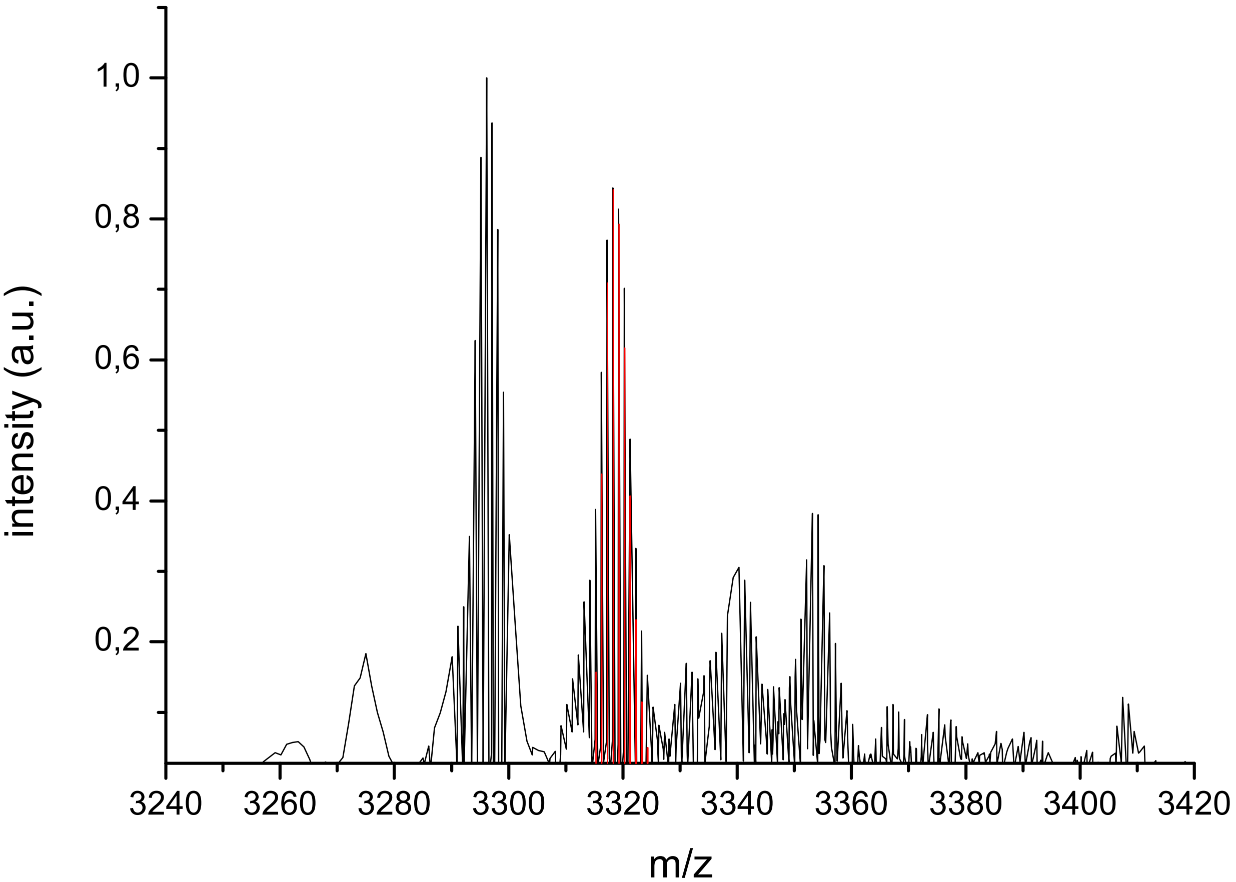

Figure S29: mass spectrum of comound **3** after cyclodehydrogenation for 3d at r.t..

After 3 d, the spectrum shows an increase in intensity for the signals at 3318 m/z and 3396 m/z, whereas the peaks around 3353 m/z, assigned to the cyclodehydrogenated dicyclohexadiene-bridged bisribbon **11**, decreased in intensity and were additionally shifted by 2 m/z to lower masses; the peaks at 3370 m/z disappeared. In the range from 3340 m/z to 3260 m/z five different peaks can be observed. The mass difference between each of them equals 22 m/z. These findings are a first indicator that a side reaction has occurred, namely a chloro-*de*-t-butylation, as the mass difference between a chlorine atom and a *t*-butyl group equals 22.1 m/z. With this having said, the peaks around 3318 m/z were more closely investigated (see *Figure S30*), as this mass could have also hinted at a bottom-up synthesis of an ultrashort CNT.

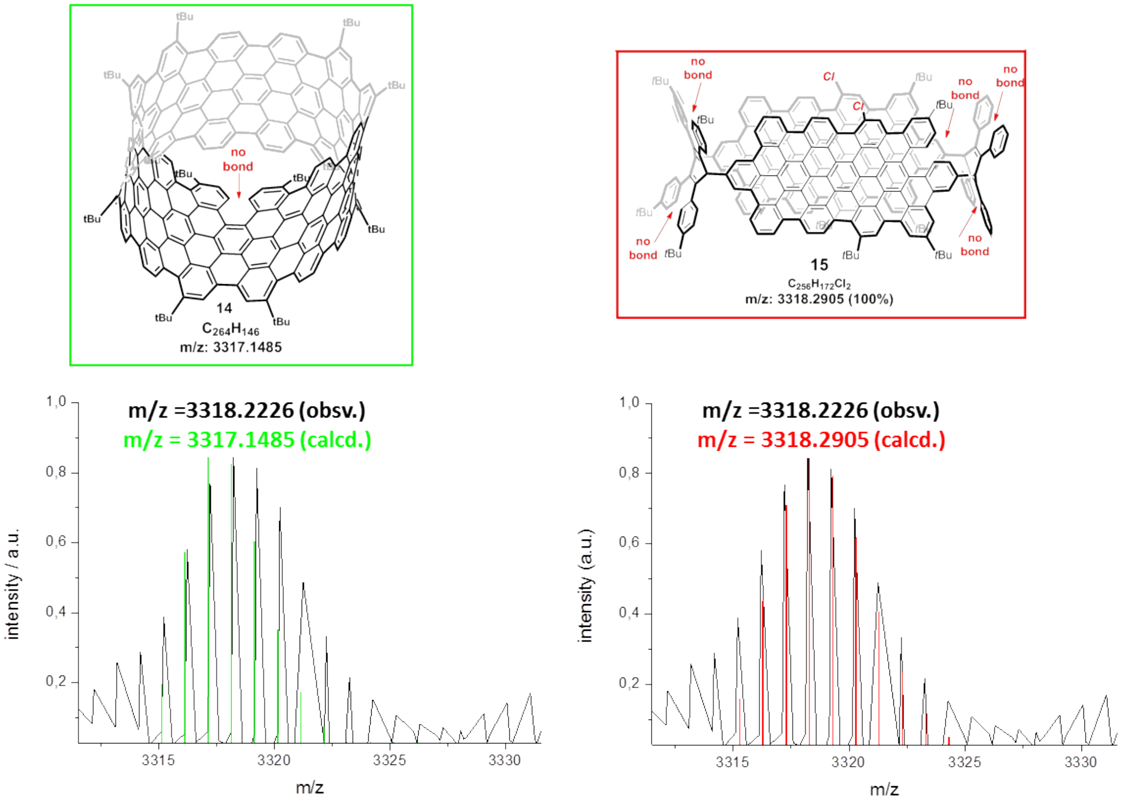

Figure S30: HR-MS (MALDI) spectrum of **108** after cyclodehydrogenation for 3 d with $FeCl_3$.

In *Figure S30*, sections of two mass spectra of **3** after cyclodehydrogenation for 3d at r.t. are shown. In the left spectrum, the hypothetical structure of an almost fully fused CNT is depicted. The calculated mass spectrum of this structure is shown in green. The difference between the observed and calculated mass is 1 m/z; the deviation is thus beyond the error limit. From these and the above described findings, one can conclude that during oxidative cyclodehyrogeation CNT **14** has not been formed. The mass spectrum for the simulated structure of **15** hints strongly at the formation of a *bis*(*de*-*t*butylated) and concomittantly dichlorinated compound, as the mass and the corresponding isotopic pattern are in good agreement. One has to state, however, that only the sum formula can be concluded and a structural hypothesis, as shown here, is entirely based on chemical intuition.

7.3 *Spectrum of triangular cycle* **8**

FT05107a Florian Golling/Müllen - FEG-424 - DCTB

ETH
Eidgenössische Technische Hochschule Zürich
Swiss Federal Institute of Technology Zurich

Acquisition Parameter

Method:	High_mass_MALDI_Witt new2			Acquisition Date:	13.06.2014 12:08:50
File Name:	D:\Data\ESI-MALDI Daten\FT051xx\FT05107a_0_F6_0_F16.d			Operator:	Rolf Häfliger
Source	Dual (MALDI/ESI)	Polarity	Positive	Nebulizer Gas	1.3 bar
Broadband Low Mass	303.2 m/z	Laser Shot Frequency	0.005 sec	Drying Gas Flow Rate	3.7 L/min
Broadband High Mass	8000.0 m/z	Laser Power	16.0 lp	Capillary	4500.0 V
No. of Cell Fills	1	No. of Laser Shots	100	Drying Gas Temperature	200.0 °C
Apodization	Sine-Bell Multiplication	Flight Time to Acq. Cell	0.002 sec		

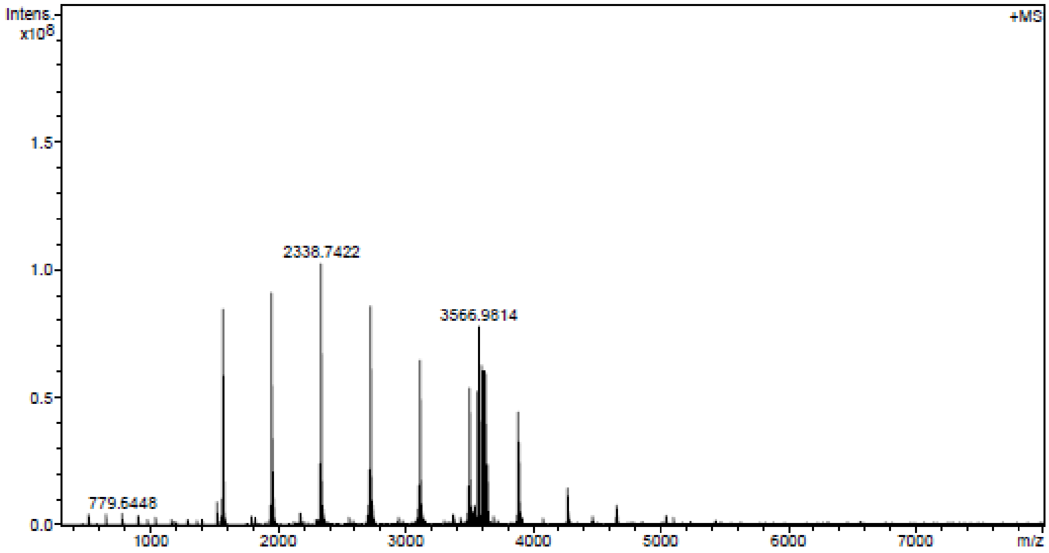

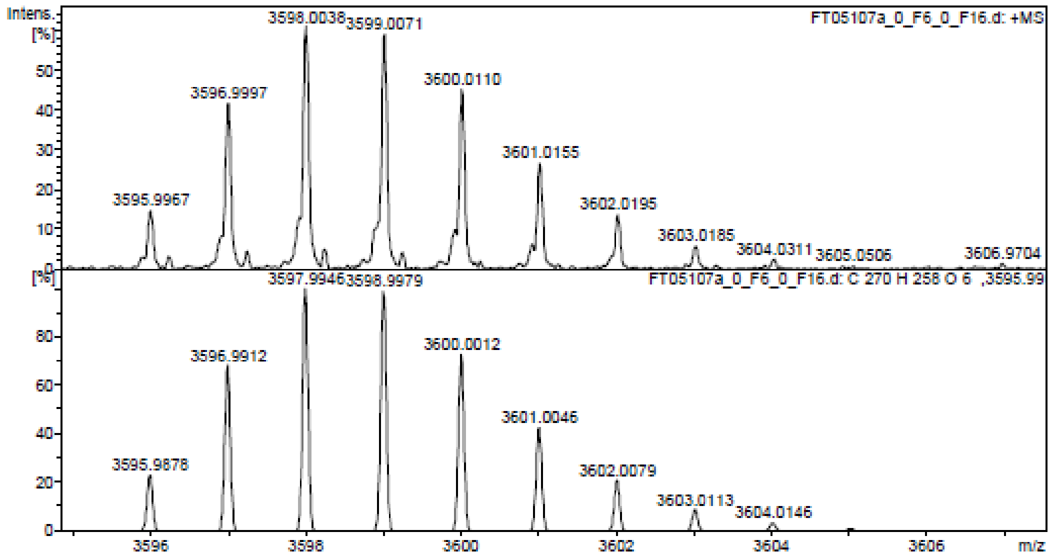

Bruker Daltonics SOLARIX   ETH - MS-Service LOC - D-CHAB   Page 1 of 3

Figure S31: MALDI-TOF-MS measurement of compound **8** (with internal calibration standard).

FT05107a Florian Golling/Müllen - FEG-424 - DCTB

ETH
Eidgenössische Technische Hochschule Zürich
Swiss Federal Institute of Technology Zurich

Evaluation Spectra / Validation Formula:

#	Formula	m/z	Meas. m/z	z	mSigma	N-Rule	err [mDa]	err [ppm]
1	C 270 H 258 O 6	3595.9878	3595.9967	1+	12.3	ok	-8.9	-2.5
1	C 270 H 258 Na O 6	3618.9776	3618.9870		6.3	ok	-9.5	-2.6
1	C 270 H 258 K O 6	3634.9515	3634.9589		17.5	ok	-7.4	-2.0

Calibration Info:

Internal calibration
Date: 16.06.2014 11:23:42
Polarity: Positive
Calibration spectrum: +MS: Scan
Reference mass list: ESI: Na-PFHA
Calibration mode: Quadratic

Calibration Table

Reference m/z	Resulting m/z	Intensity	Error [ppm]
2338.7423	2338.7422	103280984	-0.024
2724.7011	2724.7015	86036512	0.151
3110.6599	3110.6589	64624000	-0.336
3496.6188	3496.6199	54264928	0.316
3882.5776	3882.5772	44366648	-0.107

Standard deviation: 0.374

Mass List:

#	m/z	Res.	S/N	I %	FWHM
1	1566.8201	114515	1012.7	82.0	0.0137
2	1567.8235	118775	303.4	24.7	0.0132
3	1946.7640	95848	180.5	17.1	0.0203
4	1952.7807	89837	942.0	88.2	0.0217
5	1953.7833	94492	351.5	33.0	0.0207
6	2332.7258	80704	218.6	23.5	0.0289
7	2338.7422	71879	934.7	100.0	0.0325
8	2339.7443	77534	403.6	43.3	0.0302
9	2718.6823	68359	176.2	21.3	0.0398
10	2724.7015	62927	694.0	83.3	0.0433
11	2725.7019	63651	336.4	40.5	0.0428
12	3104.6404	54398	106.8	15.4	0.0571
13	3110.6589	48258	438.1	62.6	0.0645
14	3111.6609	48051	241.4	34.6	0.0648
15	3490.6043	53596	90.3	15.2	0.0651
16	3496.6199	51767	316.8	52.5	0.0675
17	3497.6234	52795	216.6	36.0	0.0662
18	3564.9714	47911	128.8	18.8	0.0744
19	3565.9776	49360	353.4	51.1	0.0722
20	3566.9814	48417	526.2	76.0	0.0737
21	3567.9844	49691	517.9	74.8	0.0718
22	3568.9866	48827	369.2	53.4	0.0731
23	3569.9904	49286	219.0	31.8	0.0724
24	3570.9958	50448	105.0	15.4	0.0708
25	3596.9997	45233	288.6	41.8	0.0795
26	3598.0038	45636	420.1	60.7	0.0788
27	3599.0071	46210	408.4	59.0	0.0779
28	3600.0110	47599	311.0	45.0	0.0756
29	3601.0155	47685	182.4	26.5	0.0755
30	3619.9890	45540	271.7	39.4	0.0795
31	3620.9924	46937	407.9	59.0	0.0771
32	3621.9964	47516	398.7	57.6	0.0762
33	3623.0011	47063	291.3	42.2	0.0770
34	3624.0022	47185	171.2	24.9	0.0768
35	3635.9622	49435	107.6	15.8	0.0736
36	3636.9659	46571	157.9	23.0	0.0781
37	3637.9678	47335	159.9	23.3	0.0769
38	3638.9724	49079	123.4	18.0	0.0741
39	3882.5772	47300	296.7	43.0	0.0821
40	3883.5805	47929	218.6	31.7	0.0810

#	m/z	Res.	S/N	I %	FWHM
1	3595.9878	45638		23.0	0.0788
2	3596.9912	45651		67.8	0.0788
3	3597.9946	45663		100.0	0.0788
4	3598.9979	45676		98.2	0.0788
5	3600.0012	45689		72.3	0.0788
6	3601.0046	45702		42.5	0.0788
7	3602.0079	45714		20.8	0.0788

Figure S32: mass list of compound **8**

7.4 Congested Cyclic Hexamer **2**

FT05783a Florian Golling/Müllen - FEG-452 - DCTB

ETH
Eidgenössische Technische Hochschule Zürich
Swiss Federal Institute of Technology Zurich

Acquisition Parameter

Method:	High_mass_MALDI_Witt new2			Acquisition Date:	18.09.2014 12:15:17
File Name:	D:\Data\ESI-MALDI Daten\FT057xx\FT05783a_0_B10_0_B12.d			Operator:	Rolf Häfliger
Source	Dual (MALDI/ESI)	Polarity	Positive	Nebulizer Gas	1.3 bar
Broadband Low Mass	303.2 m/z	Laser Shot Frequency	0.005 sec	Drying Gas Flow Rate	3.7 L/min
Broadband High Mass	8000.0 m/z	Laser Power	15.0 lp	Capillary	4500.0 V
No. of Cell Fills	1	No. of Laser Shots	100	Drying Gas Temperature	200.0 °C
Apodization	Sine-Bell Multiplication	Flight Time to Acq. Cell	0.002 sec		

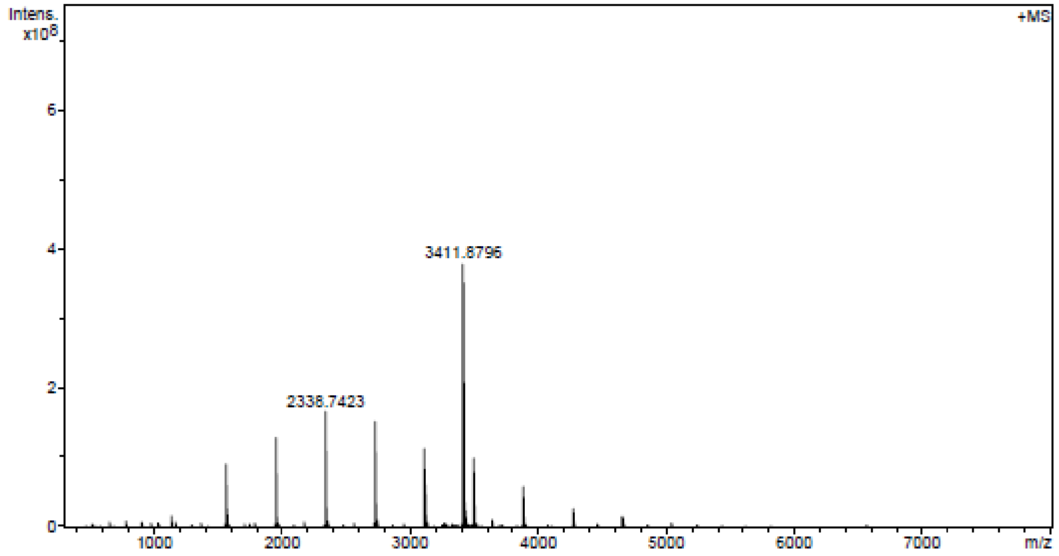

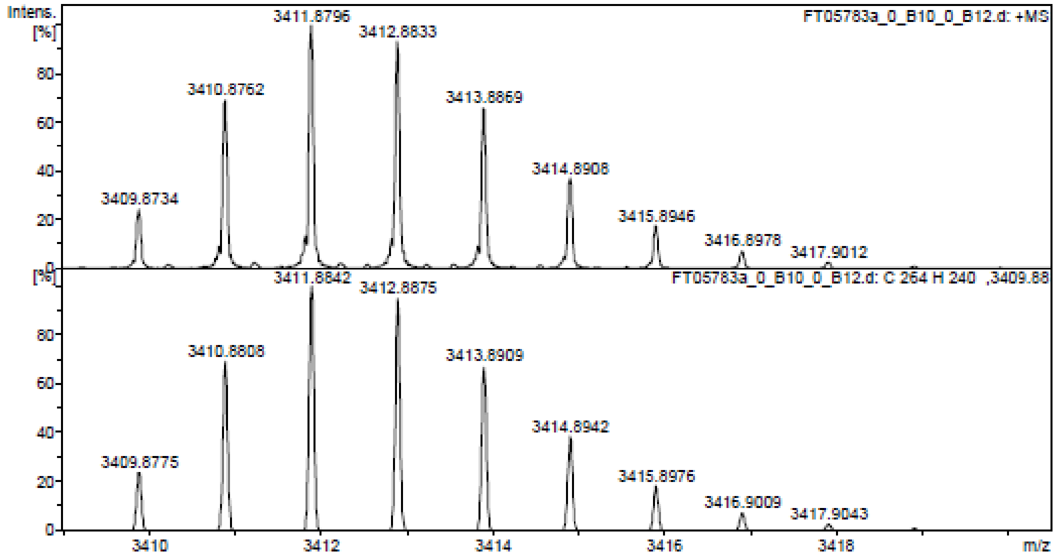

Bruker Daltonics SOLARIX — ETH - MS-Service LOC - D-CHAB — Page 1 of 3

Figure S33: MALDI-TOF-MS measurement of compound **2** (with internal calibration standard).

FT05783a Florian Golling/Müllen - FEG-452 - DCTB

ETH Eidgenössische Technische Hochschule Zürich Swiss Federal Institute of Technology Zurich

Evaluation Spectra / Validation Formula:

#	Formula	m/z	Meas. m/z	z	mSigma	N-Rule	err [mDa]	err [ppm]
1	C 264 H 240	3409.8775	3409.8734	1+	11.4	ok	4.1	1.2

Calibration Info:

Internal calibration
Date: 18.09.2014 16:37:56
Polarity: Positive
Calibration spectrum: +MS: Scan
Reference mass list: ESI: Na-PFHA
Calibration mode: Quadratic

Calibration Table

Reference m/z	Resulting m/z	Intensity	Error [ppm]
2338.7423	2338.7423	166277232	0.007
2724.7011	2724.7010	152838304	-0.035
3110.6599	3110.6601	114249680	0.052
3496.6188	3496.6187	100523160	-0.024

Standard deviation: 0.073

Mass List:

#	m/z	Res.	S/N	I %	FWHM
1	1566.8348	122195	1814.7	24.3	0.0128
2	1567.8382	126090	520.3	7.0	0.0124
3	1952.7880	97381	2191.2	34.1	0.0201
4	1953.7909	98656	755.1	11.8	0.0198
5	2338.7423	81213	2466.9	43.9	0.0288
6	2339.7454	80532	1035.6	18.4	0.0291
7	2724.7010	69402	1942.1	40.3	0.0393
8	2725.7024	65492	874.1	18.2	0.0416
9	2726.7038	67829	265.6	5.5	0.0402
10	3110.6601	58346	1185.7	30.2	0.0533
11	3111.6621	56470	635.6	16.2	0.0551
12	3112.6634	55707	223.8	5.7	0.0559
13	3409.8734	56929	810.2	24.1	0.0599
14	3410.8095	81161	309.2	9.2	0.0420
15	3410.8762	57598	2329.3	69.2	0.0592
16	3410.9416	94860	196.1	5.9	0.0360
17	3411.7788	66349	203.1	6.1	0.0514
18	3411.8124	85155	449.4	13.4	0.0401
19	3411.8796	57329	3364.7	100.0	0.0595
20	3411.9458	93167	269.4	8.0	0.0366
21	3412.8170	80208	416.8	12.4	0.0425
22	3412.8833	56792	3125.4	92.9	0.0601
23	3412.9473	80599	252.1	7.5	0.0423
24	3413.8198	45400	311.0	9.3	0.0752
25	3413.8869	57455	2226.0	66.2	0.0594
26	3413.9525	90877	186.4	5.6	0.0376
27	3414.8237	81036	178.1	5.3	0.0421
28	3414.8908	57052	1238.2	36.8	0.0599
29	3415.8946	57129	590.1	17.6	0.0598
30	3416.8978	56286	238.8	7.1	0.0607
31	3428.8847	56136	189.8	5.7	0.0611
32	3429.8900	55076	204.7	6.1	0.0623
33	3430.8950	55591	167.6	5.0	0.0617
34	3496.6187	54657	891.7	26.5	0.0640
35	3497.6215	54786	601.3	17.9	0.0638
36	3498.6242	55606	218.1	6.5	0.0629
37	3882.5818	49575	638.2	15.6	0.0783
38	3883.5844	48832	461.4	11.3	0.0795
39	4268.5509	44360	272.1	7.2	0.0962
40	4269.5550	44425	222.7	5.9	0.0961

#	m/z	Res.	S/N	I %	FWHM
1	3409.8775	56929		24.2	0.0599
2	3410.8808	56946		69.6	0.0599
3	3411.8842	56962		100.0	0.0599
4	3412.8875	56979		95.4	0.0599
5	3413.8909	56996		67.9	0.0599
6	3414.8942	57013		38.6	0.0599
7	3415.8976	57029		18.2	0.0599
8	3416.9009	57046		7.3	0.0599
9	3417.9043	57063		2.6	0.0599

Figure S34: mass list of compound **2**

7.5 *Mass spectrum of* **3**

FT05541d Florian Golling/Müllen - FEG-427 - DCTB

ETH
Eidgenössische Technische Hochschule Zürich
Swiss Federal Institute of Technology Zurich

Acquisition Parameter

Method:	High_mass_MALDI_Witt new2			Acquisition Date:	18.08.2014 13:06:01
File Name:	D:\Data\ESI-MALDI Daten\FT055xx\FT05541d_0_J2.d			Operator:	Rolf Häfliger
Source	Dual (MALDI/ESI)	Polarity	Positive	Nebulizer Gas	1.3 bar
Broadband Low Mass	303.2 m/z	Laser Shot Frequency	0.005 sec	Drying Gas Flow Rate	3.7 L/min
Broadband High Mass	8000.0 m/z	Laser Power	19.0 lp	Capillary	4500.0 V
No. of Cell Fills	1	No. of Laser Shots	100	Drying Gas Temperature	200.0 °C
Apodization	Sine-Bell Multiplication	Flight Time to Acq. Cell	0.002 sec		

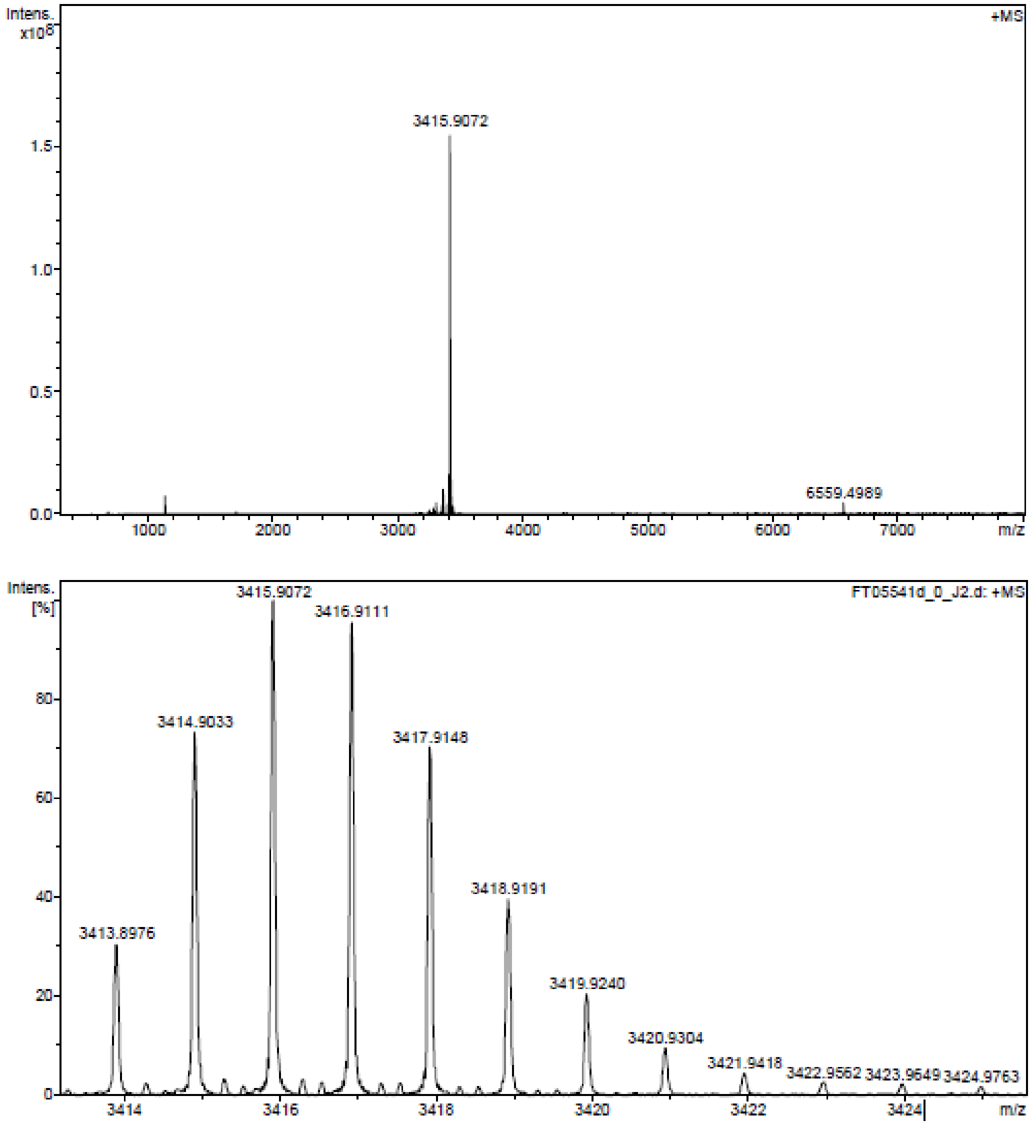

Bruker Daltonics SOLARIX ETH - MS-Service LOC - D-CHAB Page 1 of 2

Figure S35: MALDI-TOF-MS measurement of compound **3**

FT05541c Florian Golling/Müllen - FEG-427 - DCTB

ETH
Eidgenössische Technische Hochschule Zürich
Swiss Federal Institute of Technology Zurich

Acquisition Parameter

Method:	High_mass_MALDI_Witt new2			Acquisition Date:	18.08.2014 12:53:38
File Name:	D:\Data\ESI-MALDI Daten\FT055xx\FT05541c_0_I24.d			Operator:	Rolf Häfliger
Source	Dual (MALDI/ESI)	Polarity	Positive	Nebulizer Gas	1.3 bar
Broadband Low Mass	303.2 m/z	Laser Shot Frequency	0.005 sec	Drying Gas Flow Rate	3.7 L/min
Broadband High Mass	8000.0 m/z	Laser Power	19.0 lp	Capillary	4500.0 V
No. of Cell Fills	1	No. of Laser Shots	100	Drying Gas	200.0 °C
Apodization	Sine-Bell Multiplication	Flight Time to Acq. Cell	0.002 sec	Temperature	

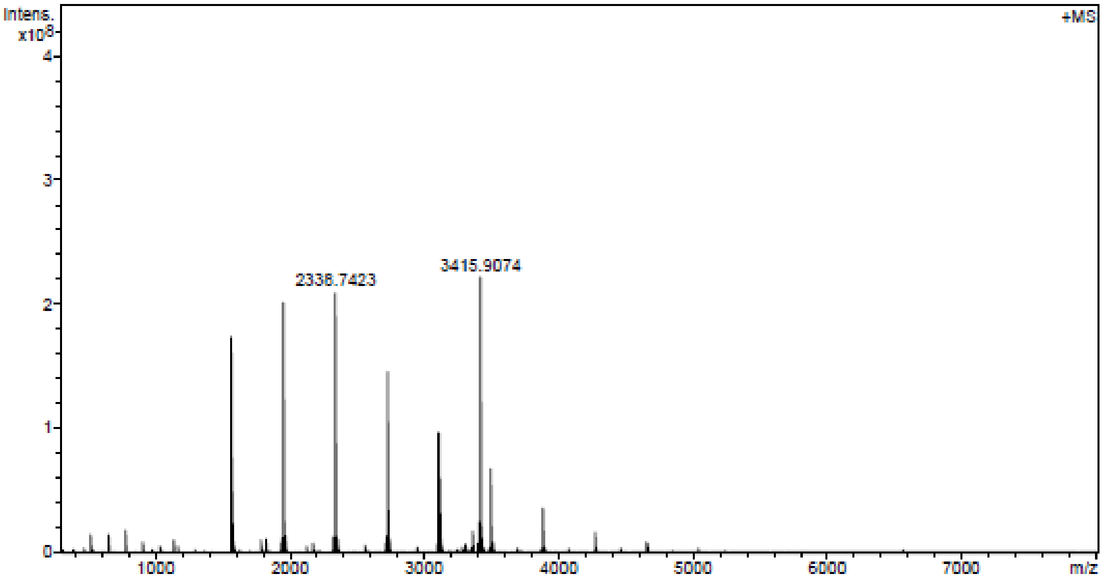

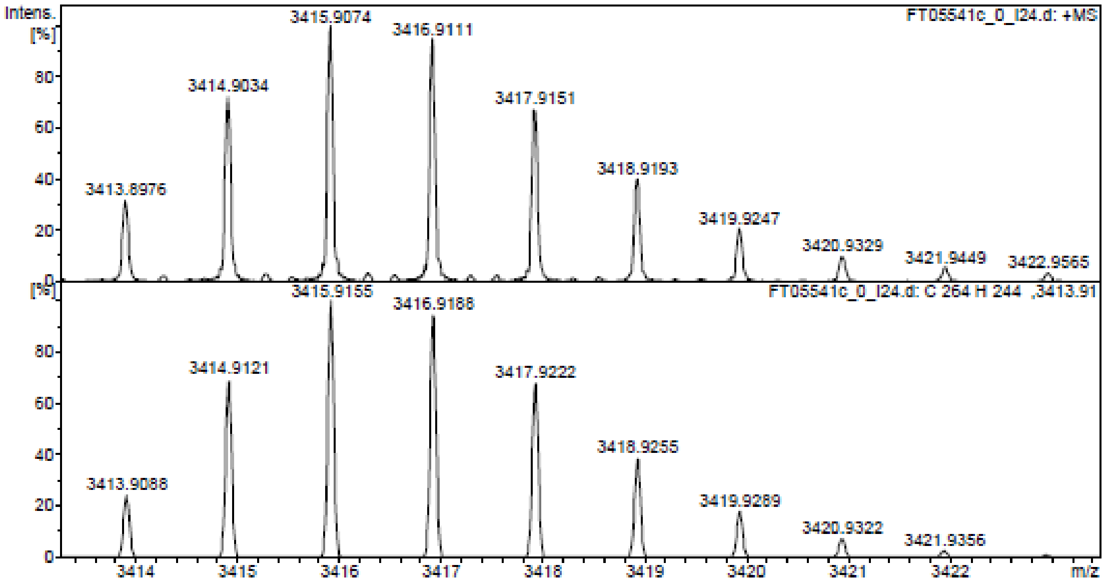

Figure S36: MALDI-TOF-MS measurement of compound **3 (**with internal calibration standard).

FT05541c Florian Golling/Müllen - FEG-427 - DCTB

ETH Eidgenössische Technische Hochschule Zürich Swiss Federal Institute of Technology Zurich

Evaluation Spectra / Validation Formula:

#	Formula	m/z	Meas. m/z	z	mSigma	N-Rule	err [mDa]	err [ppm]
1	C 264 H 244	3413.9088	3413.8976	1+	31.4	ok	11.2	3.3

Calibration Info:

Internal calibration
Date: 18.08.2014 13:00:04
Polarity: Positive
Calibration spectrum: +MS: Scan
Reference mass list: ESI: Na-PFHA
Calibration mode: Quadratic

Calibration Table

Reference m/z	Resulting m/z	Intensity	Error [ppm]
2338.7423	2338.7423	210892368	0.026
2724.7011	2724.7008	146402512	-0.109
3110.6599	3110.6603	98029864	0.122
3882.5776	3882.5775	36379624	-0.040

Standard deviation: 0.185

Mass List:

#	m/z	Res.	S/N	I %	FWHM
1	1566.8493	123066	2284.4	78.2	0.0127
2	1567.8526	122736	674.9	23.1	0.0128
3	1952.7717	214512	177.2	7.2	0.0091
4	1952.7952	94433	2268.5	91.5	0.0207
5	1953.7982	93924	836.2	33.8	0.0208
6	1954.8001	98449	188.1	7.7	0.0199
7	2338.7423	82081	2072.3	94.7	0.0285
8	2338.7753	206758	149.7	6.9	0.0113
9	2339.7458	80597	890.1	40.7	0.0290
10	2340.7481	81514	242.1	11.1	0.0287
11	2350.6993	81342	147.3	6.8	0.0289
12	2724.7008	65839	1284.6	65.7	0.0414
13	2725.7034	66717	680.9	34.9	0.0409
14	2726.7056	68744	209.9	10.8	0.0397
15	2736.6521	70915	132.6	6.9	0.0386
16	3110.6603	57712	772.6	44.0	0.0539
17	3111.6632	59095	474.1	27.0	0.0527
18	3112.6648	63470	167.2	9.6	0.0490
19	3358.8379	56238	122.1	7.7	0.0597
20	3359.8413	55819	114.7	7.2	0.0602
21	3410.8716	56166	123.0	7.8	0.0607
22	3411.8778	54804	181.8	11.4	0.0623
23	3412.8826	55093	224.2	14.1	0.0619
24	3413.8976	52881	511.4	31.9	0.0646
25	3414.9034	54948	1159.6	72.3	0.0621
26	3415.8379	112071	113.4	7.2	0.0305
27	3415.9074	55314	1604.5	100.0	0.0618
28	3416.8418	95143	106.7	6.7	0.0359
29	3416.9111	55034	1515.4	94.5	0.0621
30	3417.9151	55103	1081.8	67.5	0.0620
31	3418.9193	54366	645.2	40.3	0.0629
32	3419.9247	52883	330.2	20.7	0.0647
33	3420.9329	49436	156.4	9.8	0.0692
34	3496.6148	54345	488.1	30.5	0.0643
35	3497.6175	54866	331.2	20.7	0.0637
36	3498.6198	55364	114.6	7.2	0.0632
37	3882.5775	49460	277.0	16.3	0.0785
38	3883.5819	49740	207.1	12.2	0.0781
39	4268.5623	44246	119.8	7.6	0.0965
40	4269.5684	44179	113.1	7.2	0.0966

#	m/z	Res.	S/N	I %	FWHM
1	3413.9088	52882		24.1	0.0646
2	3414.9121	52897		69.6	0.0646
3	3415.9155	52913		100.0	0.0646
4	3416.9188	52928		95.4	0.0646
5	3417.9222	52944		68.0	0.0646
6	3418.9255	52959		38.6	0.0646
7	3419.9289	52975		18.2	0.0646
8	3420.9322	52990		7.3	0.0646
9	3421.9356	53006		2.6	0.0646

Figure S37: mass list of compound **3**.

8. Optical and Electronic Properties

8.1 UV-Vis spectra and Emission Spectrum

In this section, UV-Vis spectra and emission spectra are shown. For compound **2** and **3**, the absorption spectra are depicted. An emission spectrum can only be shown for **2**; for its C_2-symmetric congener **3**, no emission was observed.

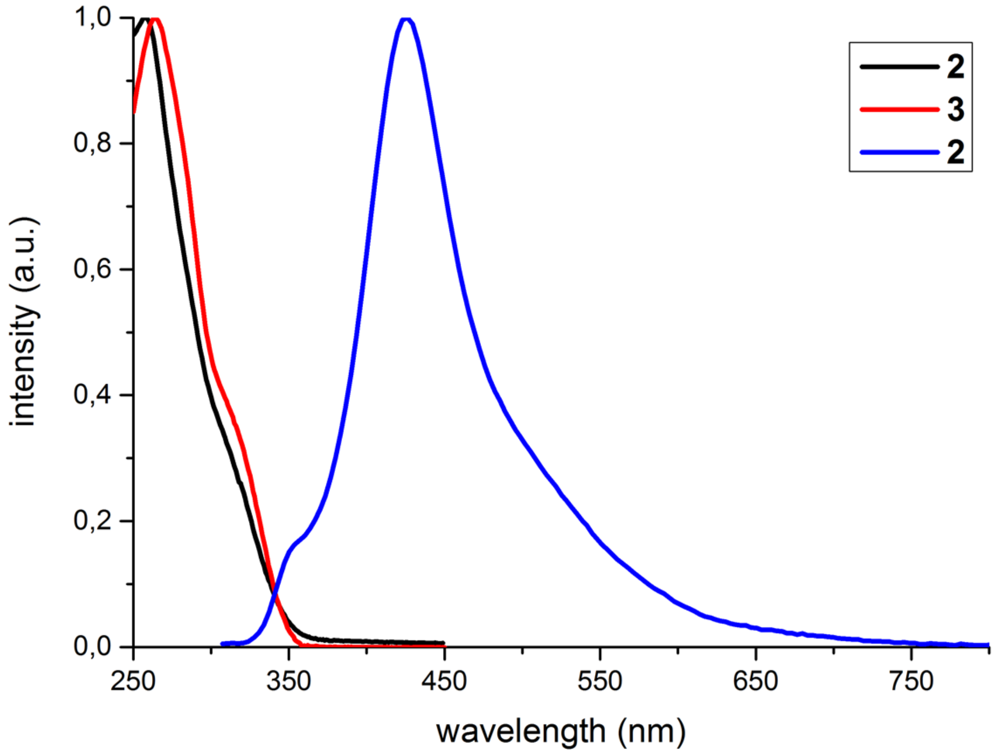

Figure S37: absorption spectra of the congested cyclohexamer (**2**, black) and its C_2-symmetric congener **3** **(**red) are shown. The emission spectrum of **2** is depicted; no emission was observed for **3**.

The absorption maxima at 258 nm and 263 nm for **2** and **3**, respectively, are in the same range as the values reported for polyphenylene cylinders, consisting of a [9]CPP or a [15]CPP ring (see *Angew. Chem. Int. Ed.* **2014**, *53*, 1525-1528). The ring size and, correspondingly, the ring strain merely influence the absorption maxima. These results are in line with size-dependent measurements, which show very small differences for the absorption maxima.[5] For "naked" CPP rings, however, the absorption maxima are bathochromically shifted (ca. 350 nm), due to higher degrees of conjugation.
The emission maximum of **2** is observed at 426 nm. This value is hypsochromically shifted in comparison to the [9]CPP based polyphenylene cylinder reported in *Angew. Chem. Int. Ed.* **2014**,

53, 1525-1528; in comparison to its higher homologue - the [15]CPP based polyphenylene cylinder - its value is bathchromically shifted ([12]CPP has its emission maxium at 450 nm) This observation is in line with the general trend observed for CPPs and the explanation, that upon excitation the large ring strain and the distortion of these CPP scaffolds is released by partial planarization of neighboring phenyl rings, which results in large Stokes shifts.

8.2 HOMO-LUMO energies

CV measurements of both compounds were not successful, since the materials degraded during CV measurement. Due to the apparent molecular properties, we could not even record an irreversible oxidation/reduction potential. This holds also true for the previously reported compounds in Angew. Chem. Int. Ed. 2014, 53, 1525–1528 and Angew. Chem. Int. Ed. 2015, DOI: 10/1002/anie.201500392.

	**2**	**3**	
**HOMO**	-5.58	-5.50	eV
**LUMO**	-0.94	-1.01	eV

Figure S38: HOMO/LUMO energies of compound **2** and **3** obtained by DFT calculations calculated at the B3LYP/6-31G(d) level of theory.

In Figure S38, the HOMO/LUMO energies of compound **2** and **3** are depicted. The values of the HOMO energies have similar values as for [12]CPP (- 5.26 eV).[6] However, the LUMO energies differ by 0.6 eV, with a higher lying LUMO of **2** and **3**. This may be attributed the increased ring strain, arising from twisted neighboring phenylene units (due to the high sterical demand exerted by phenyl substituents).

The calculated energy gaps between the HOMO and LUMO level energies of [n]CPPs are in the range of 2.5 eV (n = 4) to 3.7 eV (n = 20). For [12]CPP, the energy gap is 3.6 eV and thus significantly smaller than for **2** and **3** (4.6 eV and 4.5 eV, respectively). This can be rationalized by the computed structure of **2** and the X-ray crystal analysis of **3**. Both show that neighboring phenylene units align in an almost perpendicular manner. Hence, the degree of conjugation within these macrocycles is reduced in contrast to its parent compound [12]CPP. In addition, the high sterical demand of the neighboring groups may also explain, why the emission maximum of **2** is hypsochromically shifted in comparison to [12]CPP.

9. Literature

[1] a) K. Harada, H. Hart, C. J. Frank Du, *J. Org. Chem.* **1985**, *50*, 5524-5528; b) H. Hart, G. C. Nwokogu, *Tetrahedron Lett.* **1983**, *24*, 5721-5724; c) S. Shah, T. Concolino, A. L. Rheingold, J. D. Protasiewicz, *Inorg. Chem.* **2000**, *39*, 3860-3867.

[2] I. Ullah, R. A. Khera, M. Hussain, A. Villinger, P. Langer, *Tetrahedron Lett.* **2009**, *50*, 4651-4653.

[3] T. Ishiyama, M. Murata, N. Miyaura, *J. Org. Chem.* **1995**, *60*, 7508-7510.

[4] C. Huang, Y. Huang, N. G. Akhmedov, B. V. Popp, J. L. Petersen, K. K. Wang, *Org. Lett.* **2014**, *16*, 2672-2675.

[5] E. R. Darzi, R. Jasti, *Chem. Soc. Rev.* **2015**.

[6] T. Iwamoto, Y. Watanabe, Y. Sakamoto, T. Suzuki, S. Yamago, *J. Am. Chem. Soc.* **2011**, *133*, 8354-8361.
